# A Systematic Comparison of the Anti-Tumoural Activity and Toxicity of the Three Adv-TKs

**DOI:** 10.1371/journal.pone.0094050

**Published:** 2014-04-10

**Authors:** Qinglei Gao, Caihong Chen, Teng Ji, Peng Wu, Zhiqiang Han, Haiyan Fang, Fei Li, Yi Liu, Wencheng Hu, Danni Gong, Zeyu Zhang, Shixuan Wang, Jianfeng Zhou, Ding Ma

**Affiliations:** 1 Cancer Biology Research Center, Tongji Hospital, Tongji Medical College, Huazhong University of Science and Technology, Wuhan, Hubei, P. R. China; 2 Center of Reproductive Medicine, the First Affiliated Hospital of Zhengzhou University, Zhengzhou, China; University Hospital of Navarra, Spain

## Abstract

Adenovirus 5 vectors, known respectively as, the first generation, second generation and oncolytic adenovirus, have been studied extensively in preclinical and clinical trials. However, hitherto few systemic evaluations of the efficacy and toxicity of these adenoviral vectors that have reflected the vertical history of adenovirus based cancer gene therapy strategies have been undertaken. This study has chosen Adv-TK, the well-established adjuvant treatment in cancer, and compared its efficacy and safety with those of the two newly synthesized oncolytic adenovirus vectors encoding the HSV-TK gene, namely M7 and M8. The results obtained showed that systemic administration of 1×10^8^ pfu M7 had an anti-tumour efficacy similar to that of 3×10^10^ pfu Adv-TK whilst M8 performed even better. Furthermore, compared to Adv-TK, M7 and M8 reduced the incidence of metastases and substantially prolonged the survival time of the mice xenografted with human orthotopic gastric carcinomas with disseminated metastasis. Even more exciting, however, were the similar toxic and immune safety results obtained from the administration of high doses of M7 or M8 in comparison with Adv-TK in immunocompetent and permissive syrian hamster. The data here exhibit a comprehensive display of the efficacy and safety of the three mutants and provide evidence for the future preclinical use of the M7 and M8 viruses.

## Introduction

Recombinant adenovirus serotype 5 (Ad5) vectors have been studied extensively in preclinical models and in a range of clinical trials as a gene delivery vehicle for cancer gene therapy. Utilising first generation, second generation and conditionally replicative adenovirus vectors (CRAds), also known as oncolytic adenoviruses[1]–[10], these studies provide insight into the toxicity, pharmacology, and clinical indications of gene therapy strategies. Apart from these, hitherto few simultaneous comparisons of the efficacy and toxicity of these adenoviral vectors that carried one identical target gene and could reflect the vertical history of adenovirus based cancer gene therapy strategies have been undertaken.

Adenovirus-thymidine kinase (Adv-TK) mediated delivery of herpes simplex viruses into tumour cells is one of the best studied gene therapy approaches to facilitate the killing of tumours[11], [12]. Adv-TK, a replication-deficient adenovirus vector constructed in-house, contains an HSV-TK gene under the control of a Rous sarcoma virus long terminal repeat promoter in the region of the excised E1 adenoviral genes. This vector is now in phase III clinical trials. The phase I/II/III trials of Adv-TK reported promising results using Adv-TK alone, in combination with chemotherapeutic agents, or in combination with irradiation treatment of recurrent prostate cancers, hepatocellular carcinoma, and glioblastoma multiformes[13]. Despite these promising findings, some problems have still remained unsolved. First, Adv-TK is a replication-deficient adenovirus and is quickly prepared for elimination by the liver or rendered inactive by binding them to blood cells, neutralising antibodies, or complements, ensuring thereby that only a small proportion of the injected dose ever reaches the tumour. Because of the rapid elimination of the virus, the expression of TK is seldom sufficient for clinical cancer treatments. Second, although Adv-TK is generally well tolerated, the expression of the TK gene is not specific to tumours, as the promoter used in Adv-TK has no selectivity. Therefore, the use of adenovirus mutants that preferentially replicate in and lyse tumour cells, known as oncolytic adenoviruses, represents a promising new approach in the treatment of cancer[14].

We have previously reported two types of oncolytic adenoviral vector systems. The first was generated by replacing the 6.7K/gp19K region with a fragment of antisense complementary DNA (cDNA), specific for a target gene in an E1A CR2 region-deleted adenoviral mutant called Adv5/dE1A. The second was generated by replacing the adenovirus death protein (ADP) gene in the E3 region. Previous studies have demonstrated that in these systems, transgenes can be efficiently expressed by native E3 promoters at a level superior to that driven by the human cytomegalovirus major immediate-early promoter/enhancers (hCMV promoter). More importantly, expression of the transgenes is dependent on active viral DNA replication and mimics the expression kinetics of the E3 genes replaced by the transgenes [15]–[17].

Here two novel oncolytic adenoviral vectors have been developed, both of which share a 27-bp deletion in the E1A region of wild type Adv5 that endows them with oncolytic potentialities. In the M7 virus, HSV-TK cDNA replaced the 6.7K/gp19K of E3 region, whereas in the M8 virus, HSV-TK cDNA replaced the ADP of E3 region. This study shows administration of M7 and M8 at doses equivalent to only 0.33% of the dose of Adv-TK, combined with GCV, can eliminate disseminated metastasis in the abdominal cavity and prolong survival time in mouse models of human orthotopic gastric carcinoma. Moreover, the studies demonstrated that the safety profile and an immune profile of high doses of M7 and M8 was similar to the replication-defective adenovirus Adv-TK in Syrian hamsters, an immunocompetent, permissive animal for the replication of Ad5. Taken together, the present study demonstrates that M7 and M8 are safer and more efficacious than Adv-TK for clinical application in cancer gene therapy. To our knowledge, this study is the first to comprehensively evaluate and compare the efficacy and safety of the three mutants, and the data reported here provide evidence for future preclinical use of the M7 and M8 viruses.

## Results

### Construction and Characterization of M7 and M8

**Figure 1:**
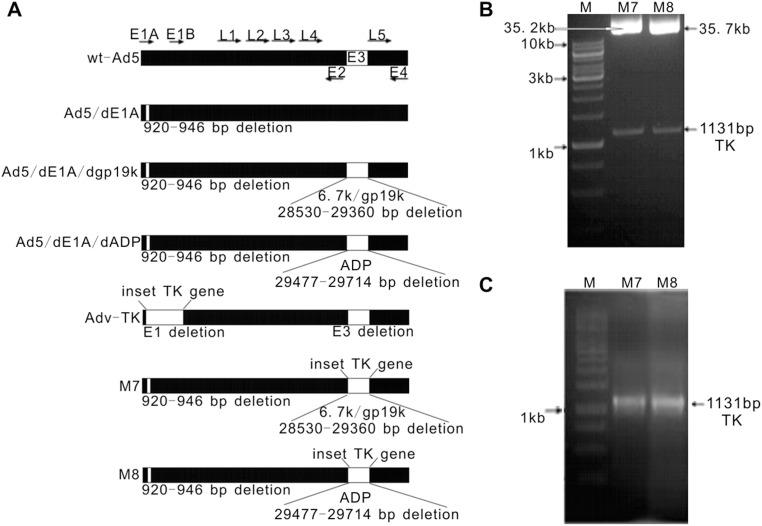
Analysis of M7 and M8 mutant adenoviruses. **A**) A 27-nucleotide sequence corresponding to Ad5 bases 920 to 946 and encoding the amino acid sequence of the E1A protein necessary for Rb protein binding was deleted to generate Ad5/dE1A. The E1 and E3 regions were deleted from Ad5 and replaced with HSV-TK gene under the control of a Rous sarcoma virus long terminal repeat promoter to generate Adv-TK. A single region corresponding to E3 6.7K and gp19K and spanning Ad5 bases 28530 to 29360 was excised from the Adv5/dE1A genome and replaced with the HSV-TK gene with a ClaI restriction site introduced at each end to generate M7. A single region corresponding to E3 ADP and spanning Adv5 bases 29477 to 29714 was excised from the Ad5/dE1A genome and replaced with a fragment of the HSV-TK gene with a ClaI restriction site introduced at each end to generate M8. **B**) Genomic structures of M7 and M8 were confirmed by ClaI digestion. Because the native ClaI restriction site (bp 917 to 922) was disrupted, the genome of M7 and M8 contained only two ClaI restriction sites located at the 5′ and 3′ ends of the HSV-TK gene cDNA. The appearance of an 1131-bp band verified the presence of the inserted HSV-TK gene and the deletion of the E1A region. **C**) Amplification of HSV-TK mRNA using the viral genomic DNA isolated from 293 cells infected with M7 or M8 as a template.

The M7 and M8 mutant adenoviruses (Figure 1A) were designed and engineered employing two genetic manipulations that were then combined to form one wt-Ad5 genome. First, a 27-bp sequence corresponding to wt-Ad5 bases 920 to 946 was deleted to generate Ad5/dE1A. This deletion induced the expression of a mutant E1A protein lacking the CR2 domain necessary for pRb binding. Second, the E3 transcription unit of the Ad5/dE1A genome, corresponding to wt-Ad5 bases 28530 to 29360, known to encode the E3 6.7K and gp19K proteins, was excised and replaced with a 1131-bp fragment of the HSV-TK gene cDNA with a ClaI restriction site introduced at each end to generate the M7 virus. Similarly, the M8 virus was generated by excising wt-Ad5 bases 29477 to 29714, known to encode the ADP protein, and replaced with HSV-TK cDNA. The genomic structures of M7 and M8 were verified by ClaI restriction and further confirmed by PCR (Figure 1B and 1C) and sequencing (data not shown).

**Figure 2:**
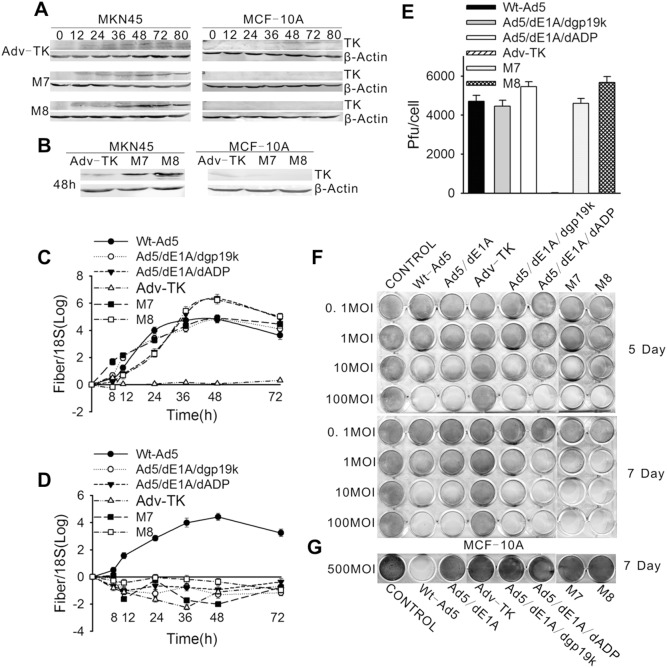
Transgene expression, viral replication and lytic activity of Adv-TK, M7 and M8 in tumour cells and normal cells. Tumour cells (MKN45) were infected with Adv-TK, M7, or M8 at an MOI of 10, and non-cancerous cells (MCF-10A) were infected at an MOI of 500. The cells were harvested at the indicated times. (**A**) and (**B**), Total protein was isolated from the cells, and TK protein was detected by western blot. (**C**) and (**D**), Total RNA was extracted and tested for levels of viral fibre mRNA by real-time quantitative PCR. The results were normalised to 18S RNA levels and are presented as the means values obtained in three independent experiments. **E**) Replication of viral mutants in tumour cells (MKN45) 48 hours after infection. **F**) Tumour cells (MKN45) were infected with mutant viruses at an MOI between 0.1 and 100 and cultured for the indicated time periods (days) before assessment of cytotoxic activity. **G**) Normal cells (MCF-10A) were infected with mutant viruses at an MOI of 500 and cultured for the indicated time periods prior to cytotoxicity assays. The results are presented as the means of values obtained in three independent experiments.

Subsequently, the characterizations, including gene expressions, replication and lytic activities of the three adenoviral mutants were compared *in vitro.* To monitor vector-mediated gene expressions, MKN45 cells were infected with the M7 and M8 viruses. At an MOI of 10 cells infected with Adv-TK expressed higher levels of TK protein at all the time points, with the highest expression of TK proteins observed, in M8-infected cells, 48 hours after infection. In contrast, only Adv-TK (neither M7- or M8) induced TK protein expression in non-proliferating, non-cancer MCF-10A cells, even at a 50-fold higher MOI (Figure 2A and 2B). Fibre transcripts, an indicator of active viral replications, were nearly undetectable in Adv-TK-infected MKN45 cells, but much higher in Ad5/dE1A/dADP and M8 infected cells 48 hours after infection (Figure 2C). Only wt-Ad5 displayed detectable replications in normal MCF-10A cells (Figure 2D). Similar results were found showing the highest viral titres being yielded in Ad5/dE1A/dADP and M8 infected MKN45 cells by viral production assays (Figure 2E). In CPE assays, although Adv-TK exerted no detectable cytopathic effects on MKN-45 cells, Ad5/dE1A/dgp19k, Ad5/dE1A/dADP, M7 and M8 showed dose-dependent lytic activities on day 5 after the infections. M8 and Ad5/dE1A/dADP displayed strongest lytic activities on day 7 after the infections, even at an MOI of 1 (Figure 2F). In contrast, in non-proliferating MCF-10A cells, the four adenoviral mutants displayed no lytic activity, even at an MOI of 500 (Figure 2G).

### M7 and M8 Viruses Displayed Superior *In vitro* Anti-tumour Effects When Compared to Adv-TK

To better assess the effect of the three adenovirus mutants *in vitro*, the dosages of GCV were optimized and the time point of the treatment in a panel of tumour cell lines varying in Rb status; metastatic potential and tissue of origin, including MKN45 (gastric carcinoma cell line with a high frequency of metastasis and wild type Rb); A549 (lung carcinoma cell line with mutant Rb); HepG2 (hepatic carcinoma cell line with mutant Rb) and A2780 (ovarian cancer cell line with mutant Rb) assayed. *In vitro* GCV sensitivity assay showed the highest non-toxic GCV treatment dose to be 100 μg/ml (Figure S1A–D).

The data for the optimized adenoviral mutant dosages showed that M7 and M8 displayed anti-tumoural activity superior to that of Adv-TK at relatively low MOI (0.1–5), but all of them displayed similar anti-tumoural activities at a high MOI (10–20). (Figure S1E–L). When the time of the treatment and the dosage of GCV were further optimised, MTT assays showed that the effects of the three viral mutants were time- and GCV dose-dependent (Figure S2, S3, S4, S5 for MKN45, A549, HepG2 and A2780 respectively).

**Figure 3:**
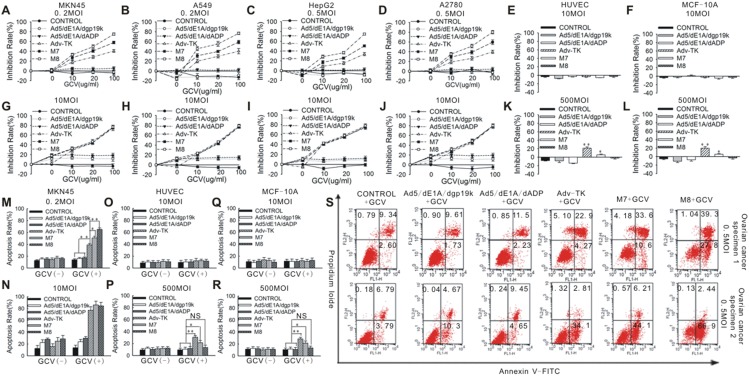
Effects of Adv-TK, M7 and M8 in cancer and non-cancerous cells *in vitro*. Cancer cells (A549, MKN45, HepG2 and A2780) were infected with various viral mutants at a MOI of 0.2 or 10 and non-cancer cells MCF-10A and HUVEC were infected with various viral mutants at a MOI of 10 or 500 in the presence of increasing concentrations of GCV (0–100 μg/ml). (**A–D**) and (**G–J**) Cell proliferation was assessed by MTT assay 72 hours after infection in cancer cells. (**E–F**) and (**K–L**) Cell proliferation was assessed by MTT assay 72 hours after infection in Non-cancerous cells. (**M–N**) Apoptosis were assessed by flow cytometry 72 hours after infection in MKN45 cancer cells. (**O–R**) Apoptosis were assessed by flow cytometry 72 hours after infection in MCF-10A and HUVEC non-cancerous cells. (**S**) Representative flow cytometric analysis of the effects of Adv-TK, M7 and M8 administered in combination with 100 μg/ml GCV in ovarian cancer cells isolated from ovarian cancer. All the results are presented as the means of values obtained in three independent experiments.

When the anti-tumour activity was compared, the data at low doses of M7 and M8 showed both had anti-tumour activity superior to that of Adv-TK in combination with any dose of GCV, whilst M8 showed the most potent effect of all the mutants (Figure 3A–D). This difference, however, disappeared at high MOIs (Figure 3G–J). In contrast, in normal cells (MCF-10A and HUVEC), the three viral mutants showed no effect at an MOI of 10 (Figure 3E–F), but at a high MOI of 500, only M8 did not affect the proliferation. M7 and Adv-TK inhibited proliferation by less than 10% and 35%, respectively (Figure 3K–L). Similar results were obtained by apoptosis assay in MKN45, HUVEC cells (Figure 3M–R), and further confirmed in A549, HepG2 and A2780 cell lines and 5 clinical ovarian cancer specimens (Figure 3S, Figure S6). These results demonstrate that M8 is the safest viral vector and exhibits the best therapeutic window of the viruses studied.

### M7 and M8 Displayed Potent Anti-tumoural Activity *In vivo*

**Figure 4:**
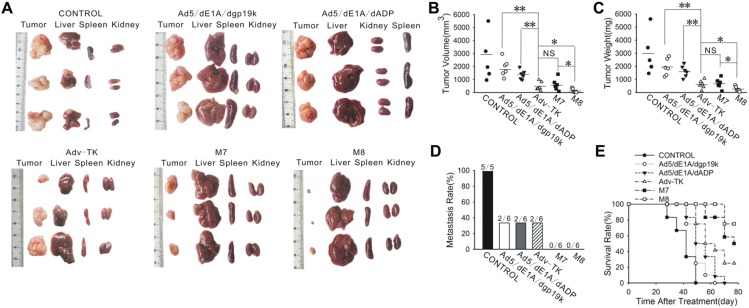
Anti-tumour effects of Adv-TK, M7 and M8 on the MKN45 orthotopic human gastric carcinoma model. Orthotopic human gastric cancer xenografted mice were randomly divided into five groups. The mice were injected with M7, M8, Ad5/dE1A/dgp19k or Ad5/dE1A/dADP vectors at a total dose of 1×10^8^ pfu or Adv-TK at a total dose of 3×10^10^ pfu or with PBS were on five consecutive days. **A**) At the end of the study, the major organs and orthotopic tumours were macroscopically observed. The findings in the liver, spleen, kidney and orthotopic tumour are shown. (**B**) and (**C**) The volume and weight of orthotopic tumours in mice treated with the various mutant viruses were evaluated at the time of study termination. **D**) The numbers of mice with metastases in each group at the time of study termination. **E**) Kaplan-Meier survival curves following the administration of Ad5/dE1A/dgp19k, Ad5/dE1A/dADP, Adv-TK, M7, M8 or PBS.

Further evaluations of the anti-tumour activities of the three adenovirus mutants in human orthotopic gastric carcinomas, also corroborated the above results, with the high frequency of metastasis characteristic of xenograft mice models[18]. In a pilot experiment, mice treated with 1×10^8^ pfu or 1×10^9^ pfu M7 or M8 resulted in similar anti-tumour efficacies, whereas at 1×10^7^ pfu dosages both M7 and M8 were less effective. Injection of 3×10^10^ pfu Adv-TK resulted in greater anti-tumour efficacy than did lower doses of Adv-TK (Figure S7). Therefore, 3×10^10^ pfu Adv-TK or 1×10^8^ pfu M7 or M8 were used in the formal experiments. As shown in Figure 4A–C, 1×10^8^ pfu M7 suppressed tumour growth to an extent similar to that achieved by administration of 3×10^10^ pfu Adv-TK. Moreover, the effect of 1×10^8^ pfu M8 was superior to that of 3×10^10^ pfu Adv-TK. All mice treated with GCV alone died before day 50 after treatment and were found to have metastases within the liver, lymph nodes and peritoneum, whereas only 2 of 6 treated with Adv-TK, and none of the M7 and M8 treated mice developed metastases (Figure 4D and Figure S8 and Table 1). Tumour-bearing mice treated with M8 exhibited survival superior to Adv-TK or M7 (Figure 4E).

**Table 1:** Metastasis observed in orthotopic human gastric carcinoma models in each group.

	Celiac lymph nodes metastasis	Liver metastasis	Spleen metastasis	Perirenal tissue metastasis	Ascites metastasis	Diaphragma metastasis
CONTROL	4/5	5/5	4/5	4/5	2/5	4/5
Ad5/dE1A/dgp19k	1/6	0/6	1/6	0/6	0/6	0/6
Ad5/dE1A/dADP	1/6	1/6	0/6	0/6	0/6	0/6
Adv-TK	2/6	2/6	1/6	0/6	0/6	0/6
M7	0/6	0/6	0/6	0/6	0/6	0/6
M8	0/6	0/6	0/6	0/6	0/6	0/6

Table 1, Data is shown as the number of mice in which metastases were observed/number of mice evaluable.

**Figure 5:**
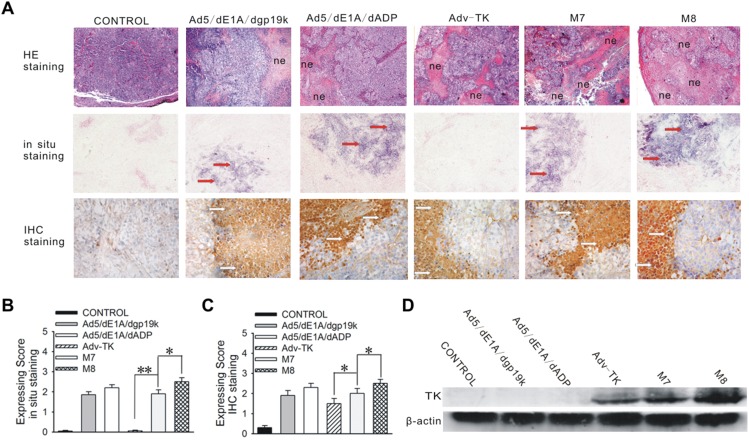
TK protein expression and viral titres of Adv-TK, M7 and M8 in orthotopic tumours. Orthotopic tumours were harvested at the time of study termination for hematoxylin and eosin (H&E) staining, western blot analysis, and immunohistochemical (IHC) and in situ hybridisation assays. **A**) Orthotopic tumours were formalin-fixed and embedded in paraffin, and 5-μm sections were prepared and stained with H&E. Viral replication was detected by *in situ* hybridisation with a digoxin-labelled viral fibre oligonucleotide probe complementary to the fibre coding region. Fibre production was determined by immunohistochemical staining using an anti-adenovirus mouse monoclonal antibody. Cells containing replicative virions were stained dark blue (red arrows), and those containing viral particles were stained brown (white arrows). **B**) Horizontal bar graph showing the *in situ* hybridisation expression scores for viral fibre in tumours. **C**) Horizontal bar graph showing immunohistochemical scores in tumours. **D**) Protein was isolated from orthotopic tumours, and 40 μg of total protein isolated from tumour tissues was subjected to western blot analysis.

While mild tumour necrosis was seen in Ad5/dE1A/dgp19k, Ad5/dE1A/dADP and Adv-TK-infected mice, increased tumour necrosis which decreased tumour cell density was detected in M7 infected mice. Perhaps the most remarkable changes were observed in M8 infected mice, which exhibited severe diffuse necrosis throughout the entire sections examined (Figure 5A). Fibre transcripts were detected preferentially in local tumour sites after i.v. injection of Ad5/dE1A/dgp19k, Ad5/dE1A/dADP, M7 and M8 but not Adv-TK, with the most active replication detected in M8 infected mice (Figure 5A and 5B). Similarly, the strongest viral particles and TK protein expressions were readily detected in M8 infected mice (Figure 5C and 5D).

### M7 and M8 Exhibited Safety Profiles Similar to that of Adv-TK

**Figure 6:**
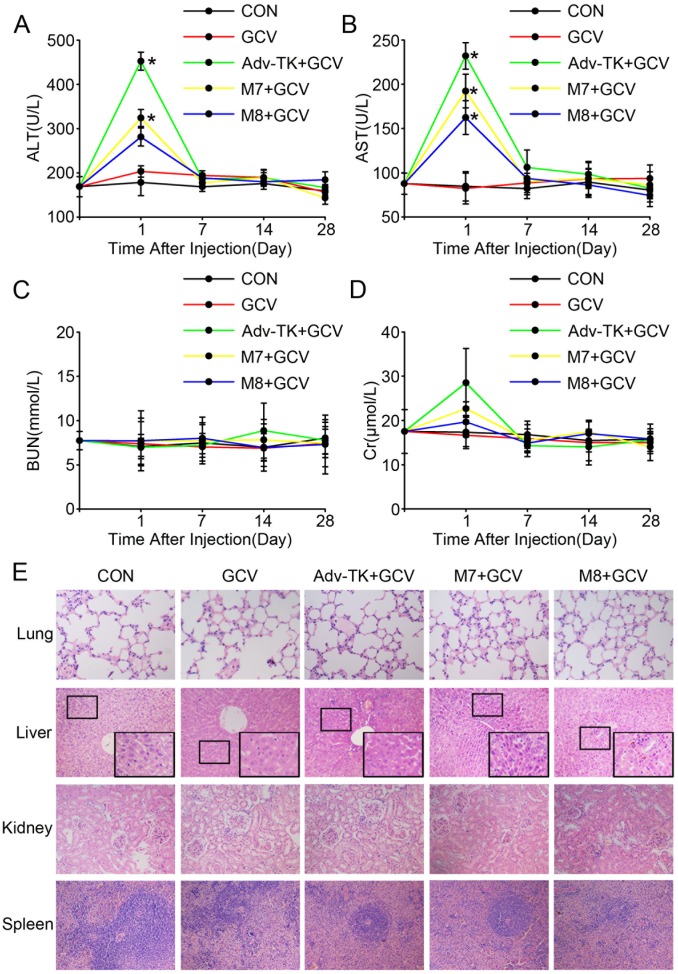
Evaluation of the safety profiles of Adv-TK, M7 and M8 in immunocompetent and permissive Syrian hamsters. Hamsters were randomly assigned into five groups and injected with saline, GCV, Adv-TK, M7 or M8 (1.0×10^12^ vp/kg) once daily for five consecutive days. 12 hours later, the hamsters were injected i.p. once daily for five consecutive days with GCV (50 mg/kg/d) in 100 μL of saline. The hamsters were sacrificed at the indicated time. Blood and tissues were harvested for the detection of haematological indices and histopathological analyses. **A**) Alanine aminotransferase (ALT). **B**) Aspartate aminotransferase (AST). **C**) Blood urea nitrogen (BUN). **D**) Creatinine (Cr). (*P<0.05). **E**) The main tissues and organs (lung, liver, kidney and spleen) were harvested for hematoxylin and eosin (H&E) staining. Representative histological images of tissues 1 days after virus injection are shown.

Immunocompetent, permissive Syrian hamsters were used to further evaluate the safety profile of the three adenovirus mutants *in vivo.* No hamsters died during the injection and following 4-week observation period. No mouse was observed to have any signs of cyanosis, spasm, or any other abnormalities in behaviour, fur, neurological response, breathing, or defecation within 2 hours after high doses (1.0×10^12^ vp/kg/d) of adenovirus injections. Body weight and food consumption (Table 2 and 3) didn’t show any difference among the five groups. All virus-injected mice became slightly lethargic approximately 10 min after virus injection; the symptoms resolved in about 30 min. The blood biochemical indicators ALT, AST, BUN, and Cr were analyzed to identify possible vector-induced toxicity. Although all three viruses caused a significant elevation of serum ALT and AST at day 1, especially for Adv-TK injected group (*P*<0.05) (Figures 6A and B), they all recovered by day 7. No significant changes were observed in BUN levels until the end of the observation period (Figure 6C). While one hamsters injected with the Adv-TK displayed obviously increased Cr on day 1 after the injection, others in Adv-Tk group had the mild increased Cr as same as M7 and M8 groups, they all recovered by day 7 (Figure 6D).

**Table 2:** Influence of the three virus mutant on mouse body weight in toxicity test (g).

Group	Day0	Day1	Day7	Day14	Day28
CON	118.5±5.65	120.33±2.17	124.33±3.29	128.72±6.74	130.85±8.42
GCV	118.75±2.4	121.89±4.32	114.44±2.68	122.85±4.55	130.03±5.55
Adv-TK+GCV	116.75±4.57	116.5±5.31	96.53±3.48	108.88±3.28	118.44±3.56
M7+GCV	117.5±4.29	117.00±3.85	95.44±2.57	110.32±4.11	126.75±4.76
M8+GCV	121.33±4.51	118.5±3.54	102.3±2.66	118.65±5.31	128.74±4.78

Note. n = 6 in each group.

**Table 3:** Influence of the three virus mutant on food consumption in toxicity test (g/mouse/d).

Group	Day0	Day1	Day7	Day14	Day28
CON	10.42±1.75	10.83±1.78	11.45±1.30	12.26±1.11	13.89±2.13
GCV	11.55±1.44	10.03±0.89	11.86±1.33	12.78±1.06	13.67±3.12
Adv-TK+GCV	11.56±2.03	11.75±1.39	8.79±0.68	10.57±1.35	14.05±2.55
M7+GCV	12.04±5.31	12.05±2.75	8.81±0.59	9.96±2.11	13.77±2.26
M8+GCV	11.66±3.54	12.11±0.88	12.63±2.54	13.54±2.51	14.45±3.55

Note. n = 6 in each group.

Histopathological analyses of tissues that are sensitive to adenovirus, including lung, liver, kidney, and spleen, were also performed. While M7 and M8 infected hamsters showed mild discrete necrotic foci and inflammatory cell infiltration scattered throughout the parenchyma, preferentially localizing in periportal regions, Adv-TK showed moderate necrotic foci and inflammatory cell infiltration by histopathological examination of the liver at day 1 (Figure 6E). These lesions mostly recovered by day 7 post-injection. No treatment-related lesions were observed among the three mutants in the pathology of the lung, kidney and spleen.

#### Hematopoietic effects of the three Adv-TK mutants in syrian hamsters

**Figure 7:**
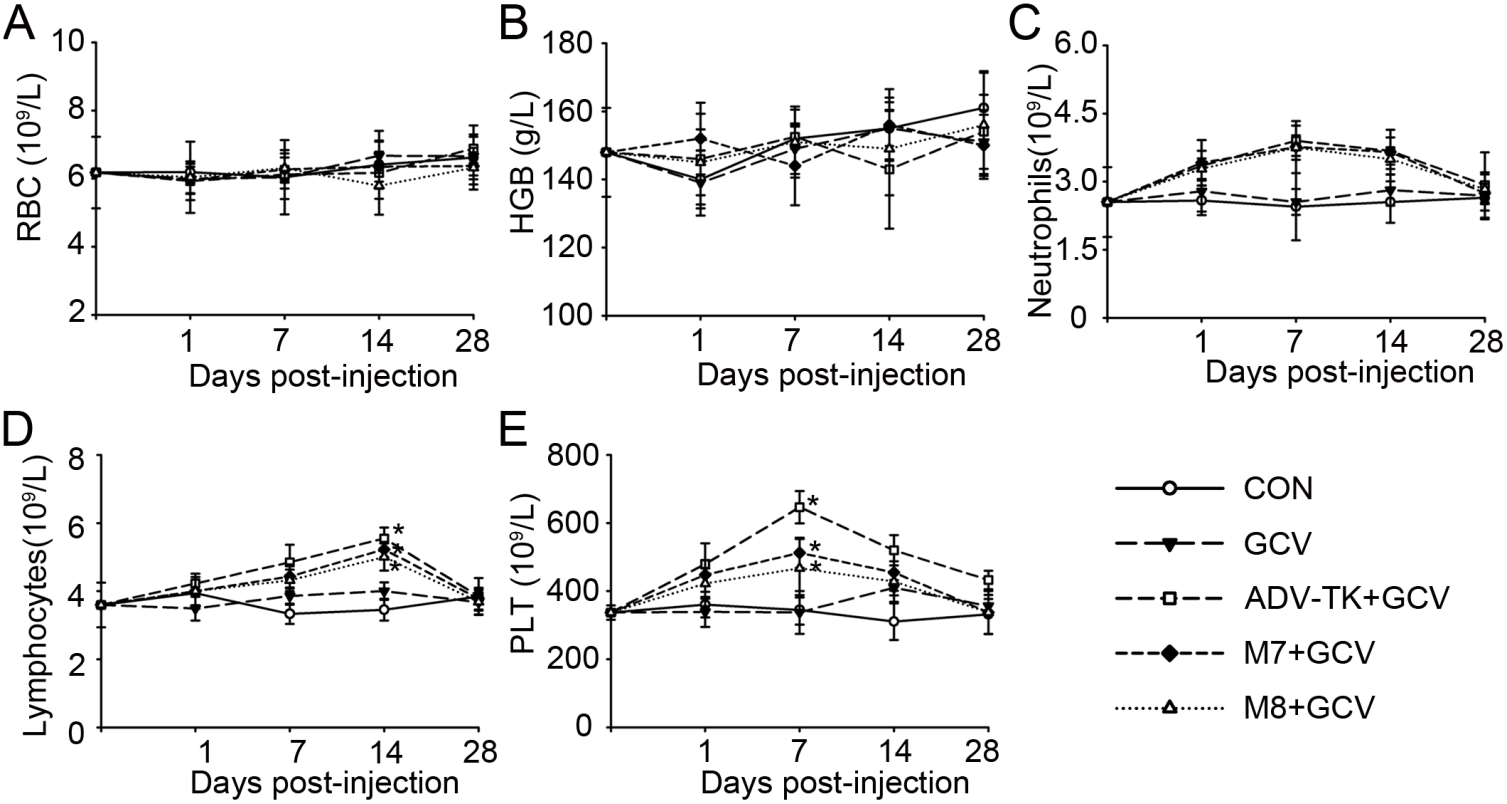
Hematopoietic effects of the three Adv-TK mutants in Syrian hamsters. Hamsters were randomly assigned into five groups and injected with saline, GCV, Adv-TK, M7 or M8 (1.0×1012 vp/kg) once daily for five consecutive days. 12 hours later, the hamsters were injected i.p. once daily for five consecutive days with GCV (50 mg/kg/d) in 100 μL of saline. The hamsters were sacrificed at the indicated time. Blood routine examination indicated as erythrocyte counts (A), hemoglobin concentration (B), neutrophils (C) and lymphocytes (D) and platelet counts (E) was performed to evaluate hematopoietic effects of the three Adv-TK mutants in Syrian hamsters. (*P<0.05).

The three Adv-TKs didn’t affect erythropoiesis in hamsters as evidenced by the number of circulating erythrocytes and hemoglobin (Figure 7A and B), but affected on white blood cells. As shown in Figure 7C, neutrophils were increased from all three experimental groups at similar levels on day 1, and elavated to the highest levels at day 7, then began to decrease and recovered at the end of the observation day. This tendency was different in lymphocyte, as increase in lymphocyte levels was significantly seen at day 14 in all three Adv-TKs injected groups (*P*<0.05) (Figure 7D). Similar to neutrophils, platelet counts were also significantly increased at day 7 in all three Adv-TKs injected groups (*P*<0.05), particular in Adv-TK group, but it remained higher than GCV controls even at day 28 (Figure 7E).

#### M7 and M8 stimulated primary immune responses similar to that induced by Adv-TK *in vivo*

**Figure 8:**
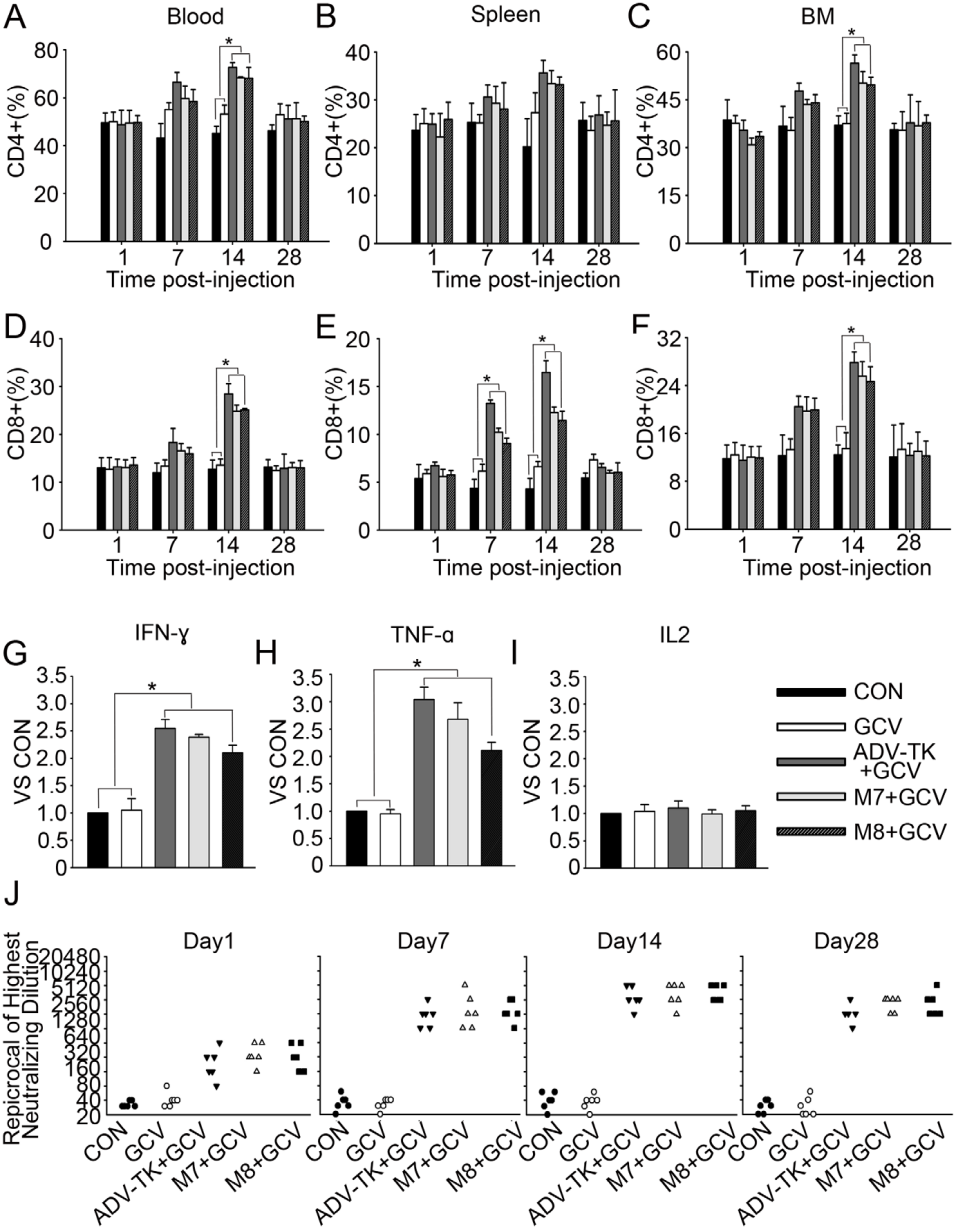
The primary immune responses stimulated by the three mutant viruses *in vivo*. Hamsters were injected with 1.0×10^12^ vp/kg of each adenovirus once daily for five consecutive days and with 50 mg/kg/d GCV in 100 μL of saline once daily for five consecutive days. The hamsters were sacrificed at the indicated time. (**A**)–(**F**) The influence of the three mutant viruses on the frequency of CD4+ T lymphocytes and CD8+ T lymphocytes in the peripheral blood, spleen and bone marrow were analysed by flow cytometry. **G**) Representative serum levels of IFN-γ 1 day post-injection. **H**) Representative serum levels of TNF-a 1 day post-injection. **I**) Representative serum levels of IL-2 1 day post-injection. **J**) Neutralising antibody to adenovirus was quantified in a cytopathic effect-based neutralisation assay. (*P<0.05).

To determine whether injection of the oncolytic adenoviruses M7 and M8, which induced tumour lysis, might elicit stronger immune responses than Adv-TK, primary immune responses were determined in immunocompetent and permissive Syrian hamster. Significant elavation of CD4+ T lymphocytes in all three viruses compared with matched controls was only seen in blood and bone marrow but not in spleen at day 14 (*P*<0.05) (Figure 8A–C). Although all three viruses caused a significantly increased CD8+ T lymphocytes seperated from peripheral blood, bone marrow and spleen at day 14 (*P*<0.05), they all recovered at 28 day (Figure 8D–F).

Transient elevations in serum levels of IFN-γ and TNF-a but not IL-2 were observed as early as 1 day after the injections (*P*<0.05) (Figure 8G–I), and recovered to normal levels by day 7(data not shown). Adenovirus-neutralising antibodies were consistently elevated and peaked on day 14 after injections (median of 2,560 in [i.e., 1∶2,560]), and remained consistently high to a median of 1280 [i.e., 1∶1280] in Adv-TK group and 2560 [i.e., 1∶2560] in M7 and M8 group at the end the experiment (28 day). There were no significant differences among each of the viral treated groups following primary immunisation (Figure 8J).

## Discussion

The present study was undertaken to compare the efficacy and safety of the first generation of replication-deficient adenovirus Adv-TK with those of the oncolytic adenoviruses M7 and M8. The gene expressions, replications, oncolysis, toxicity and immunity induction were evaluated after systemic delivery. These studies were necessary because although replication-deficient and oncolytic adenoviruses have both proven efficacy and relatively safety in clinical settings, clinical response rates to these viruses are inconsistent. Moreover, few studies have simultaneously comprehensive compared the efficacy and toxicity of these classes of adenoviruses which carried one identical target gene and used as a systemically administered single agent in one paper. We have developed a family of replicating Ad vectors for the treatment of cancer[15]–[20]. Since Adv-TK is one of the most representative and extensively studied approaches, it can mirror in miniature the evolution of the development of adenovirus based cancer gene therapy in the past thirty years. Therefore, in this study, two new oncolytic adenoviral vectors for the delivery of HSV-TK cDNA (M7 and M8) were designed and engineered and compared against vectors in classical first generation Adv-TK. Our findings may have important implications for clinical strategies and eventually, clinical outcomes.

The reasons why M8 exhibit superior anti-tumour effects relative to M7 need to be addressed. We have previously demonstrated that the expression of transgenes in this adenoviral system is dependent on active viral DNA replication and mimics the expression kinetics of the E3 genes that have been replaced by transgenes[15]–[17]. The 6.7K/gp19K region, which was replaced in the M7 virus, functions in the evasion of detection and eradication by Ad-specific cytotoxic T lymphocytes (CTLs)[21]–[23]. However, unlike other E3 genes, the ADP gene replaced in the M8 virus is expressed almost exclusively during the late phase of the viral infection[24] and is responsible for the efficient lysis and release of the progeny virus[25], [26]. Cells infected with an ADP-deleted Ad live longer and maintain protein synthesis over the extended course of the viral infection[25]–[27]. Consequently, a cell infected with an ADP-deleted virus should serve as a ‘factory’ for therapeutic protein synthesis, viral replication-derived genomic amplification and extended viral release, resulting, in turn, in increased lethality to tumour cells with a stronger “bystander effect”. Therefore were increased expressions of TK and enhanced viral replication in M8 injected orthotopic tumours observed, which resulted in dramatic reductions in metastases and enhanced survival in a refractory orthotopic carcinoma xenograft model associated with disseminated metastases. These findings are encouraging enough to warrant clinical evaluation.

Concerns about safety in immunised hosts limit the clinical utility of therapeutic adenovirus vectors. This is especially true for oncolytic adenoviruses. Although the overall safety of oncolytic adenoviruses have been demonstrated in a number of clinical trials[10], [28]–[33], viral gene expressions and/or replication in and lysis of tumour cells may promote the induction of cell-mediated immunity to uninfected tumour cells and elicit strong inflammatory responses[34]. As data shown in the safety profiles of the three Adv-TK mutants, the most impressive toxicity of the three viruses seemed to cause a defect in liver functions in hamsters as evidenced by histopathological lesions and anomalous serum ALT and AST parameters at day 1, which could recover to normal by day 7. As both M7 and M8 were tumor selective replication adenovirus, which could selectively replicated in tumor cells but not in primary normal hepatocytes[15], this phenomonon could be explained by the reason that liver was the primary target organ of Adv-TKs. Different from the data previously published by Lichtenstein et al[35], although the same hepatocellular necrosis observed in hamster, the toxicity results mainly happened at day 7 but not at day 1, indicating viral replication of INGN007 played a role in the extent of liver damage in hamsters. As 1000 times of M7 and M8 were used in safety assays compared to efficacy assays for consecutive five days, which was the highest doses used *in vivo* to our knowledge, the damage to liver and other organs were not too serious to organ failure, suggesting the large therapeutic windows for future clinical use.

Thus, by testing the efficacy in xenografted model of human orthotopic gastric carcinomas with disseminated metastasis and the safety in immunocompetent, permissive Syrian hamsters, this study suggests that the M7 and M8 viruses are safer and more efficacious than Adv-TK and suggests a potential clinical application for these viruses in cancer gene therapy. However, further modifications of M7 and M8 are required to overcome the native tropism of adenovirus and to retarget them to specific tumour sites to achieve full therapeutic efficacy. To our knowledge, this study is the first to comprehensively evaluate and compare the efficacy and safety of the three mutant viruses. The findings reported here provide evidence supporting the preclinical development of the M7 and M8 viruses in the future.

## Materials and Methods

### Viruses and Cells

All Adv5 mutants used in the present studies were constructed in in-house laboratories following protocols detailed elsewhere. Ad5/dE1A with deletion of amino acids 121–129 in E1A CR2 was previously constructed[36]. M7 and M8 were derived from Ad5/dE1A through replacement of the ADP and gp19k open reading frame in the E3 region by a fragment of HSV-TK cDNA, respectively. Ad5/dE1A/dgp19k and Ad5/dE1A/dADP were derived from Ad5/dE1A through deletion of the gp19k and ADP open reading frame and used as a vector control for M7 and M8, respectively. The replication-deficient adenovirus vector Adv-TK, containing HSV-TK gene under control of a Rous sarcoma virus long terminal repeat promoter in the region of the excised E1 adenoviral genes, was constructed in the in house laboratories as previously described[37]. Wt-Ad5 and the following cell lines A549, HepG2, MCF-10A (Non-transformed mammary epithelial cell lines) and 293 cell lines were purchased from the American Type Culture Collection (Manassas, VA), A2780, MKN-45 was obtained from the China Center for Type Culture Collection (Shanghai, P.R. China). Human umbilical cord endothelial cells (HUVEC) were purchased from Lonza (Allendale, NJ) and cultured following the manufacturer’s instructions.

### Verification of M7 and M8

M7 or M8-infected 293 cells were cultured for 3 days. Viral DNA was isolated and digested with ClaI for electrophoresis separation or for PCR verification. Primers used for PCR were as follows: TK P1 5′-ATCGATACCATGGCTTCGTACCCCG-3′, P2 5′-ATCGATTCAGTTAGCCTCCCCCAT-3′.

### Isolation and Culture of Ovarian Cancer Cells

Ovarian cancer specimens were collected from Tongji Hospital, affiliated to Tongji Medical College, HUST. Isolation and culture of ovarian cancer cells were done as described previously[38].

### Western Blot

Preparation of protein samples and Western blot were done as described previously[39]. Antibodies to TK and β-actin were purchased from Santa Cruz Biotechnology (Santa Cruz, CA).

### *In vitro* Cytotoxicity Assay

Seventy percent confluent MKN45 cells were infected with Ad5/dE1A/dgp19k, Ad5/dE1A/dADP, wt-Ad5, Adv-TK, M7, or M8 for 90 minutes at a range of MOI from 0.01 to 100 and then transferred to complete culture medium. Non-cancerous MCF-10A cells were infected with the same adenovirus as indicated above for 90 minutes at MOI of 500. For cytotoxicity assays, the culture plates were stained with 0.1% crystal violet solution (Sigma Chemical Co., St. Louis, MO) at various times.

### Real-time PCR

Quantitative PCR was performed using the SYBR Green Real Time PCR method. Each sample was tested in triplicate, and relative gene expression was analyzed using the 2^−ΔΔCT^ method, and results were expressed as fold induction compared with the untreated group. PCR was performed with a 5′ sense primer (5′- ACTATATGGACAACGTCAACCCATT −3′) and a 3′ antisense primer (5′- ACCTTCTGAGGCACCTGGATGT −3′) for fiber of Ad5. To amplify 18S RNA internal control, a 5′ sense primer (5′- AGT CCC TGC CCT TTG ACA CA −3′) and a 3′ antisense primer (5′- GAT CCG AGG GCC TCA CTA AAC −3′) were used.

### MTT Assay

Tumor cells (MKN45, A549, HepG2, A2780) and non-tumorigenic cells (MCF-10A, HUVEC) were seeded at a density of 5×10^3^ cells per well in 96-well plates. After 24 h, cells were infected with Ad5/dE1A, Adv-TK, M7 and M8 in 50 μL of culture medium per well at various MOIs, and 50 μL of complete medium containing various concentrations of GCV was co-administered. MTT (Promega Madison, Wisconsin, USA) assay was done at indicated times as described previously[40].

### Apoptosis Assay

Tumor cells (MKN45, A549, HepG2, A2780) were infected with various viral mutants at MOI of 0.2 or 0.5 or 10, MCF-10A and HUVEC were infected with various virus mutants at MOI of 10 or 500. 100 μg/ml of GCV was co-administered, and cells were collected at indicated time, stained and analyzed using a FACSort Flow Cytometer (Becton Dickinson, San Jose, CA) as described previously [15].

### *In situ* Hybridization and Immunohistochemistry

*In situ* hybridization and immunohistochemistry was performed on continuous formalin-fixed, paraffin-embedded tissue sections to detect adenoviral particles as described previously[17], [18].

### Mouse Tumor Models

All animal protocols were approved by the Institutional Animal Care and Use Committee of Tongji Medical College. Specific pathogen free athymic BALB/c nude mice were purchased from the Animal Experimental Center of Slaccas (Shanghai, China). Orthotopic human gastric cancer xenografts with disseminated metastasis mouse models were established as described previously[18]. After 10 days as indicated by Huang et al[18], 1×10^8^ pfu of Ad5/dE1A/dgp19k, Ad5/dE1A/dADP, M7 or M8 and 3×10^10^ pfu of Adv-TK was injected intravenously for five consecutive days (5–6 mice per group). Then GCV (60 mg/kg/d) in 100 μL of saline was injected intraperitoneally once daily for fourteen consecutive days. The mice were monitored twice a day, and they were sacrificed 28 days after treatment or when they became moribund. Tumours, spleens, livers, and lymph nodes (portal, mesenteric, inguinal, and retroperitoneal) were collected at necropsy and processed for histopathologic evaluation. An identical experiment was done in parallel to assess the survival rate in each group, and mice were sacrificed by inhalating CO2 when they were in the state of cachexia.

### Toxicology Assay

Male and female golden Syrian hamsters were purchased from Experiment Animal Center of Wuhan Institute of Biological Products (Wuhan, China), aged 5–6 weeks, weighing 112–126 g, with male and female each occupying one half, were assigned into five groups semirandomly by sex, and injected with 1.0×10^12^ vp/kg/d of Adv-TK, M7 and M8 once daily for five consecutive days in three experimental groups. The matched control group given saline or GCV (50 mg/kg/d) in 100 μL of saline was injected intraperitoneally once daily for five consecutive days. After finishing injections, general symptoms, body weight, food consumption were checked daily for clinical signs of toxicity for consecutive 4 weeks. Mice were euthanized at the indicated time points, whole blood and serum samples were collected through cardiac puncture of all animals for hematological and serum biochemistry examination. Necropsies were completed, select organs including lung, liver, spleen, kidney were weighed and harvested for viral quantification and hematoxylin and eosin (H&E) staining.

### Fluorescence Stained Cell Sorting Analysis

Syrian hamsters were sacrificed at the indicated time after adenovirus injections, lymphocytes were separated respectively from peripheral blood, bone marrow and spleen using the mouse lymphocyte separation medium (TBD) according to the protocols, and stained with anti-mouse CD4-allophycocyanin (clone GK1.5) (eBiosciences) and anti-rat CD8b-phycoerythrin (clone 341) (eBiosciences). The stained cells were incubated for 30 minutes at 4°C in the dark, then subjected to flow cytometry analysis.

### Measurement of Serum Cytokines

Syrian hamsters were sacrificed at the indicated time after injection and serums were harvested. Levels of IFN-γ, TNF-a, IL-2 in sera were evaluated using the corresponding ELISA kits (ebioscience American) as per the manufacturer’s recommendations.

### Adenovirus Neutralizing Antibody Titration

Syrian hamsters were sacrificed at the indicated time after injection and serums were harvested. Adenovirus neutralizing antibody titration was detected as described previously[41].

### Statistical Analysis

Statistical values are presented as mean ± SD. The significance of differences between groups was assessed by the two-tailed Student’s t-test. To compare tumor size between difference groups in mouse experiments, we performed a one-way ANOVA followed by Newman-Keuls. Statistical significance was defined as *P*<0.05. Survival was analyzed by the Kaplan–Meier method and differences were analyzed by a log-rank test. All P values were two-sided. SPSS version 11.5 software was used for statistical analysis.

## Supporting Information

Figure S1 **The effect of various virus mutants on tumor cells with and without GCV. (A–D).** tumor cells (MKN45, A549, HepG2, A2780) on 96-well plates were administered with wide dose range of GCV(0–800 ug/ml), and the cell proliferation inhibition rate were checked by MTT assay at indicated time. **(E–H).** tumor cells (MKN45, A549, HepG2, A2780) on 96-well plates were infected with various virus mutants with wide dose range (0.1–20 MOI), the cell proliferation inhibition were checked by MTT assay. **(I–L).** tumor cells (MKN45, A549, HepG2, A2780) on 96-well plates were infected with various virus mutants with wide dose range (0.1–20 MOI) and co-administered with 100 ug/ml GCV, the cell proliferation inhibition were checked by MTT. All the results were the means of three independent experiments.
(TIF)

Figure S2 **The three virus mutants showed dose and time dependent effect with GCV in MKN45 cells**. MKN45 cells were infected with various virus mutants at a MOI of 10 or 0.2 and co-administered with wide dose range of GCV(0–100 ug/ml), the cell proliferation inhibition were checked by MTT assay at indicated time. All the results were the means of three independent experiments.
(TIF)

Figure S3 **The three virus mutants showed dose and time dependent effect with GCV in A549 cells.** A549 cells were infected with various virus mutants at a MOI of 10 or 0.2 and co-administered with wide dose range of GCV(0–100 ug/ml), the cell proliferation inhibition were checked by MTT at indicated time. All the results were the means of three independent experiments.
(TIF)

Figure S4 **The three virus mutants showed dose and time dependent effect with GCV in HepG2 cells.** HepG2 cells were infected with various virus mutants at a MOI of 10 or 0.5 and co-administered with wide dose range of GCV (0–100 ug/ml), the cell proliferation inhibition were checked by MTT at indicated time. All the results were the means of three independent experiments.
(TIF)

Figure S5 **The three virus mutants showed dose and time dependent effect with GCV in A2780 cells.** A2780 cells were infected with various virus mutants at a MOI of 10 or 0.5 and co-administered with wide dose range of GCV (0–100 ug/ml), the cell proliferation inhibition were checked by MTT at indicated time. All the results were the means of three independent experiments.
(TIF)

Figure S6 **Flow cytometry detected the antitumoral effect of the three virus mutants on tumor cells (A549, HepG2, A2780).** Tumor cells (A549, HepG2, A2780) on 12-well plates were infected with various virus mutants at a MOI of 0.2 of A549 **(A)**, 0.5 MOI of HepG2, **(B)** and 0.5 MOI of A2780 **(C)** or at a MOI of 10 A549 **(D)**, HepG2 **(E)** and A2780 **(F)**, and co-administered with or without 100 ug/ml GCV. The cell apoptosis were checked by flow cytometry at indicated time. A representative flow cytometric analysis of the cell apoptosis rate of isolated primary ovarian cancer cells infected with various virus mutants at a MOI of 0.5 and co-administered with 100 ug/ml GCV was showed in **(G)**.
(TIF)

Figure S7 **The antitumor effect of variant doses of the three viral mutant in pilot experiment.** Variant doses of the three virus mutant were injected into the orthotopic gastric carcinoma xenograft model separately, the tumor volume in each group was measured at the time of study termination.
(TIF)

Figure S8 **Metastases observed in orthotopic human gastric carcinoma model in each group. (A–F)** showed representative histological images of gastric carcinoma in **(A)**. celiac lmph nodes, **(B).** peritoneum, **(C).** diaphragma, **(D).** adrenal glands and kidney ascites, **(E).** liver, **(F).** spleen.
(TIF)
