# Correction: A Systematic Comparison of the Anti-Tumoural Activity and Toxicity of the Three Adv-TKs

**DOI:** 10.1371/journal.pone.0153540

**Published:** 2016-04-07

**Authors:** Qinglei Gao, Caihong Chen, Teng Ji, Peng Wu, Zhiqiang Han, Haiyan Fang, Fei Li, Yi Liu, Wencheng Hu, Danni Gong, Zeyu Zhang, Shixuan Wang, Jianfeng Zhou, Ding Ma

The authors would like to correct Figs 5 and 6, as errors were introduced in the preparation of these figures for publication. In Fig 5, the stain for *in situ* Ad5/dE1A/dADP was derived from the same image as M7 and has an area of overlap. In Fig 6, the image for Kidney Adv-TK+GCV appears as a duplicate of Kidney GCV in panel E. The authors have provided corrected versions of Figs 5 and 6 here.

**Fig 5:**
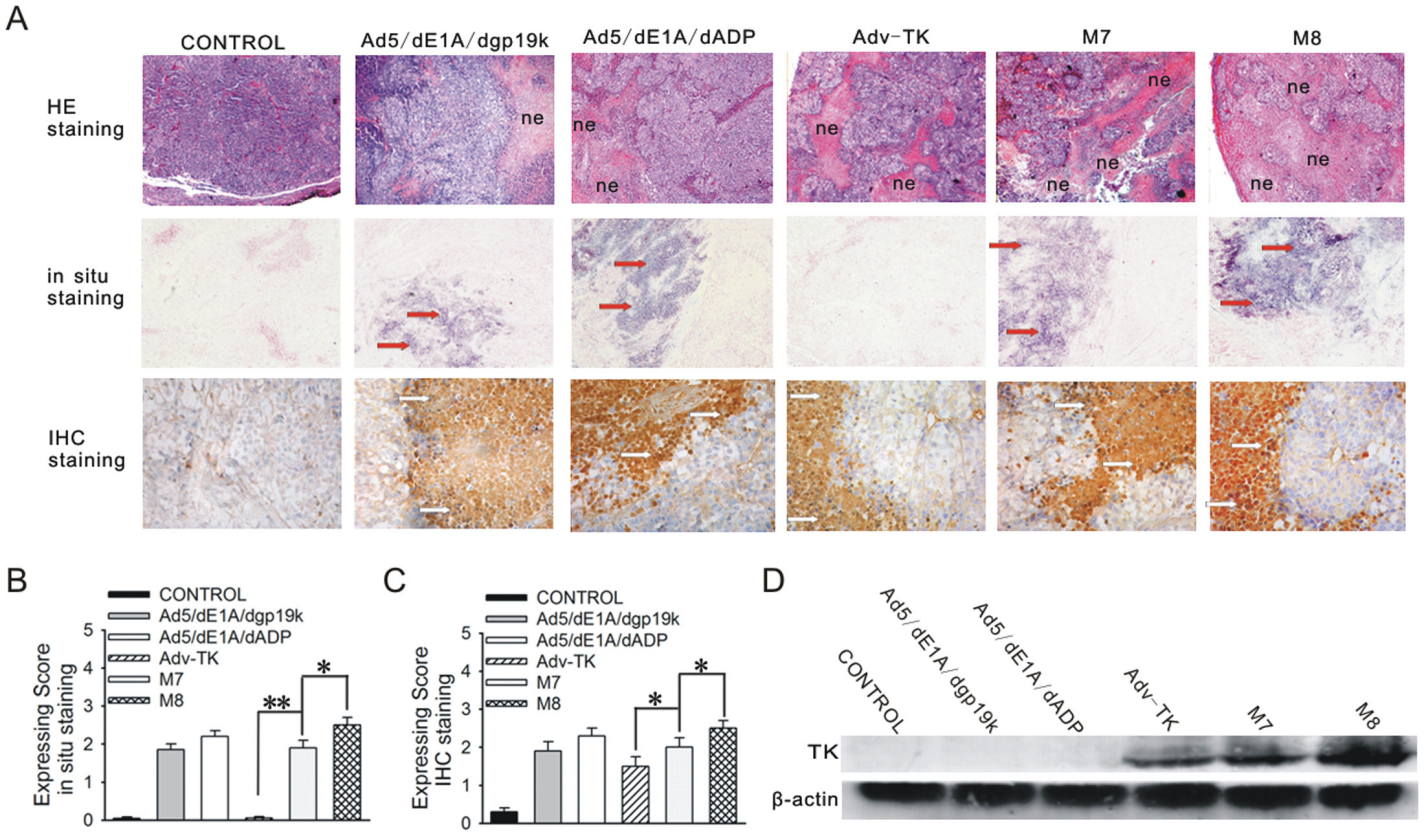
TK protein expression and viral titres of Adv-TK, M7 and M8 in orthotopic tumours. Orthotopic tumours were harvested at the time of study termination for hematoxylin and eosin (H&E) staining, western blot analysis, and immunohistochemical (IHC) and in situ hybridisation assays. **A**) Orthotopic tumours were formalin-fixed and embedded in paraffin, and 5-μm sections were prepared and stained with H&E. Viral replication was detected by *in situ* hybridisation with a digoxin-labelled viral fibre oligonucleotide probe complementary to the fibre coding region. Fibre production was determined by immunohistochemical staining using an anti-adenovirus mouse monoclonal antibody. Cells containing replicative virions were stained dark blue (red arrows), and those containing viral particles were stained brown (white arrows). **B**) Horizontal bar graph showing the *in situ* hybridisation expression scores for viral fibre in tumours. **C**) Horizontal bar graph showing immunohistochemical scores in tumours. **D**) Protein was isolated from orthotopic tumours, and 40 μg of total protein isolated from tumour tissues was subjected to western blot analysis.

**Fig 6:**
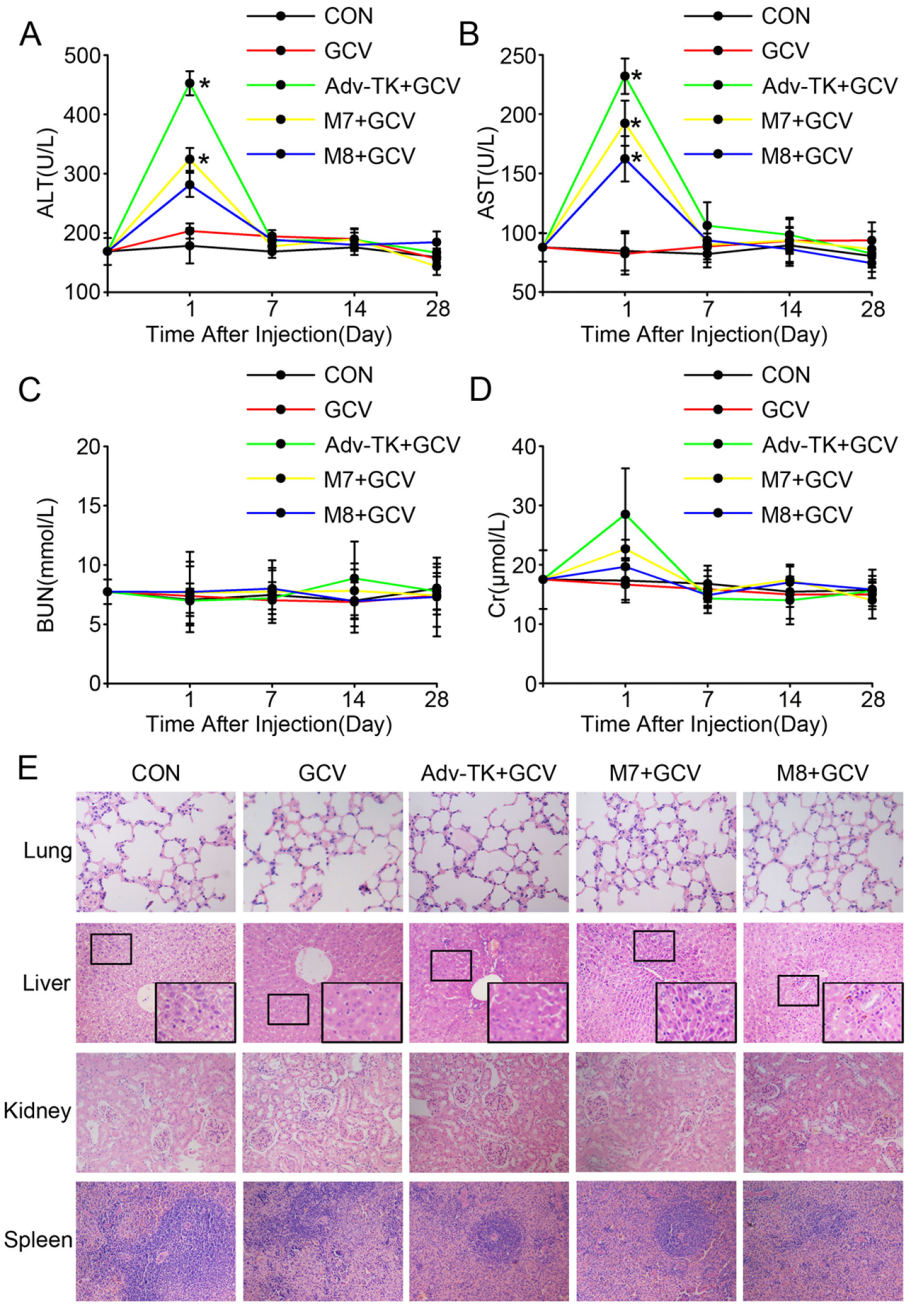
Evaluation of the safety profiles of Adv-TK, M7 and M8 in immunocompetent and permissive Syrian hamsters. Hamsters were randomly assigned into five groups and injected with saline, GCV, Adv-TK, M7 or M8 (1.0×10^12^ vp/kg) once daily for five consecutive days. 12 hours later, the hamsters were injected i.p. once daily for five consecutive days with GCV (50 mg/kg/d) in 100 μL of saline. The hamsters were sacrificed at the indicated time. Blood and tissues were harvested for the detection of haematological indices and histopathological analyses. **A**) Alanine aminotransferase (ALT). **B**) Aspartate aminotransferase (AST). **C**) Blood urea nitrogen (BUN). **D**) Creatinine (Cr). (*P<0.05). **E**) The main tissues and organs (lung, liver, kidney and spleen) were harvested for hematoxylin and eosin (H&E) staining. Representative histological images of tissues 1 days after virus injection are shown.

The authors confirm that these changes do not alter their findings. The authors have provided the underlying images for all figures in the original article as Supporting Information.

## Supporting Information

S1 File Underlying images for Figs 1–3 and a table listing the file names of underlying images for all figures. (ZIP)

S2 File Underlying images for Fig 4 and Fig 5. (ZIP)

S3 File Underlying images for Fig 6, S6 Fig and S8 Fig. (ZIP)
