# Supplementary figures and images for: A Systematic Comparison of the Anti-Tumoural Activity and Toxicity of the Three Adv-TKs
Source: PLoS One. 2014 Apr 10;9(4):e94050. doi: 10.1371/journal.pone.0094050 (PMC3983249; doi:10.1371/journal.pone.0094050)

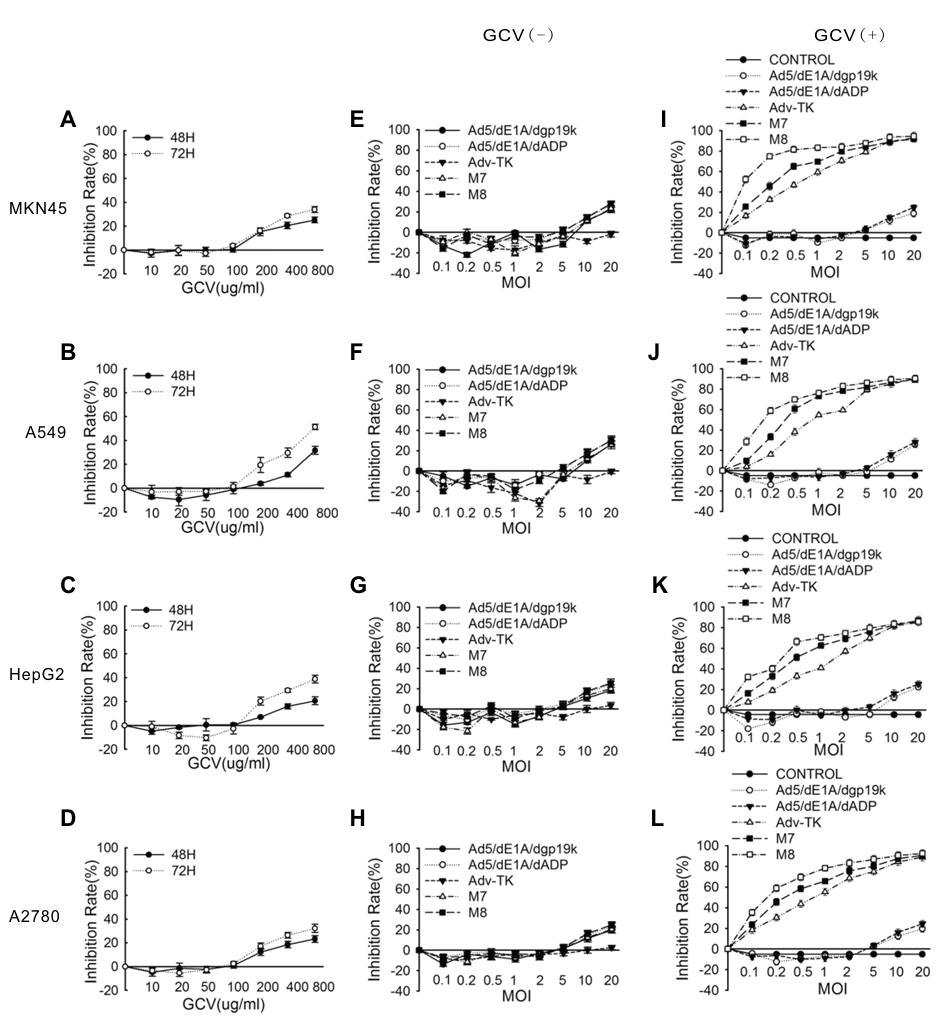

Supplement: Figure S1 — The effect of various virus mutants on tumor cells with and without GCV. (A–D). tumor cells (MKN45, A549, HepG2, A2780) on 96-well plates were administered with wide dose range of GCV(0–800 ug/ml), and the cell proliferation inhibition rate were checked by MTT assay at indicated time. (E–H). tumor cells (MKN45, A549, HepG2, A2780) on 96-well plates were infected with various virus mutants with wide dose range (0.1–20 MOI), the cell proliferation inhibition were checked by MTT assay. (I–L). tumor cells (MKN45, A549, HepG2, A2780) on 96-well plates were infected with various virus mutants with wide dose range (0.1–20 MOI) and co-administered with 100 ug/ml GCV, the cell proliferation inhibition were checked by MTT. All the results were the means of three independent experiments. (TIF) [file pone.0094050.s001.tif]

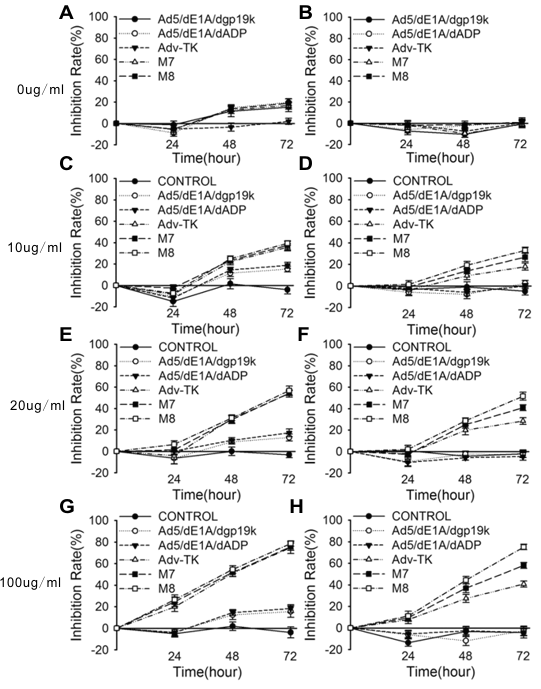

Supplement: Figure S2 — The three virus mutants showed dose and time dependent effect with GCV in MKN45 cells. MKN45 cells were infected with various virus mutants at a MOI of 10 or 0.2 and co-administered with wide dose range of GCV(0–100 ug/ml), the cell proliferation inhibition were checked by MTT assay at indicated time. All the results were the means of three independent experiments. (TIF) [file pone.0094050.s002.tif]

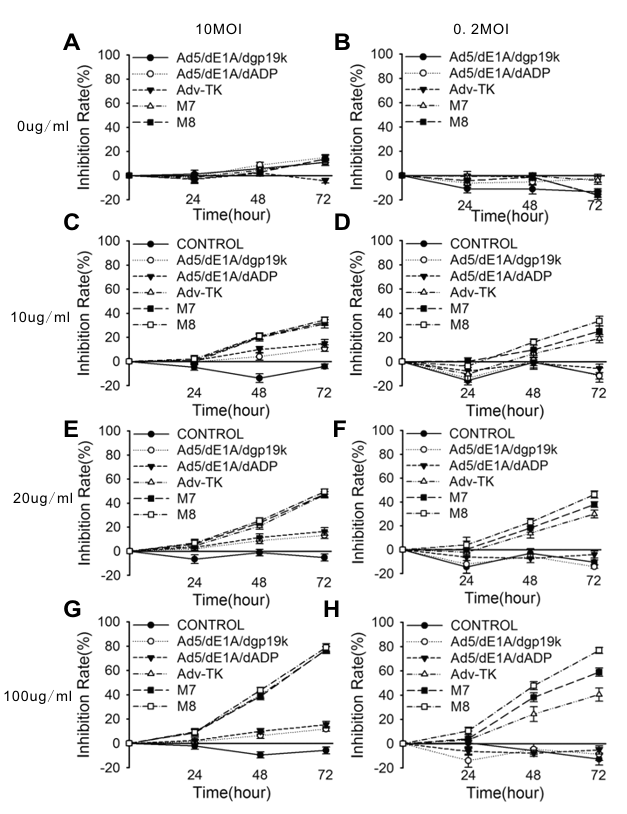

Supplement: Figure S3 — The three virus mutants showed dose and time dependent effect with GCV in A549 cells. A549 cells were infected with various virus mutants at a MOI of 10 or 0.2 and co-administered with wide dose range of GCV(0–100 ug/ml), the cell proliferation inhibition were checked by MTT at indicated time. All the results were the means of three independent experiments. (TIF) [file pone.0094050.s003.tif]

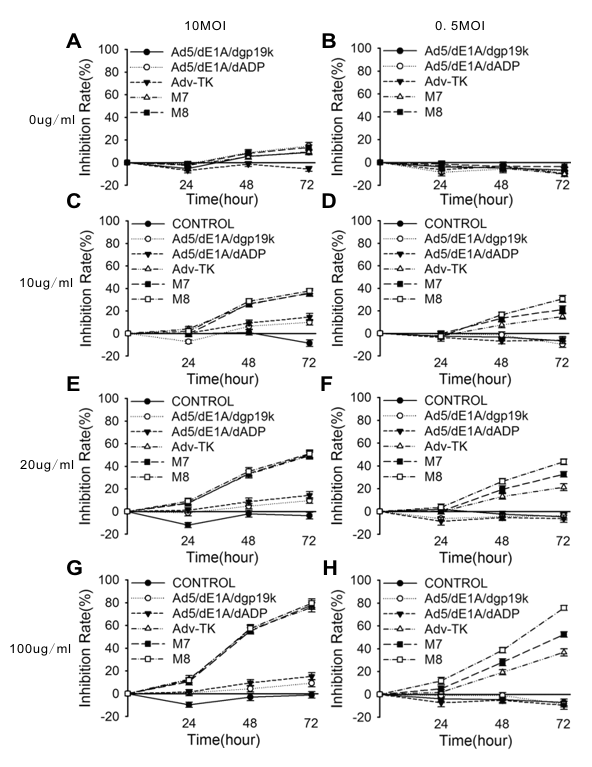

Supplement: Figure S4 — The three virus mutants showed dose and time dependent effect with GCV in HepG2 cells. HepG2 cells were infected with various virus mutants at a MOI of 10 or 0.5 and co-administered with wide dose range of GCV (0–100 ug/ml), the cell proliferation inhibition were checked by MTT at indicated time. All the results were the means of three independent experiments. (TIF) [file pone.0094050.s004.tif]

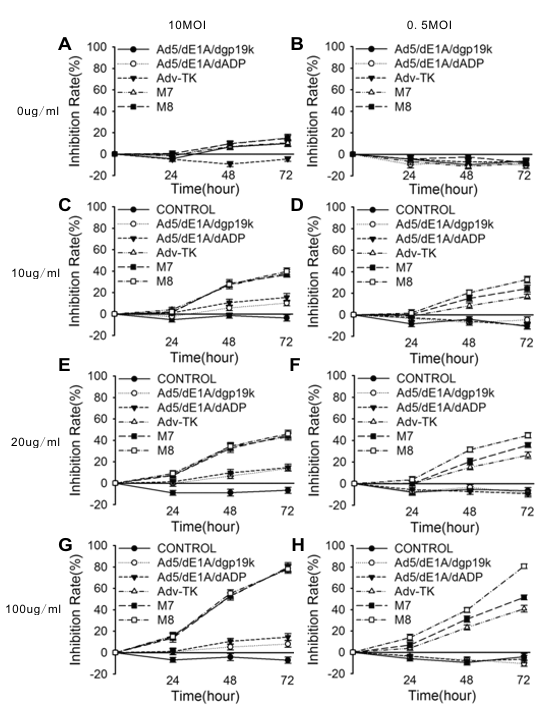

Supplement: Figure S5 — The three virus mutants showed dose and time dependent effect with GCV in A2780 cells. A2780 cells were infected with various virus mutants at a MOI of 10 or 0.5 and co-administered with wide dose range of GCV (0–100 ug/ml), the cell proliferation inhibition were checked by MTT at indicated time. All the results were the means of three independent experiments. (TIF) [file pone.0094050.s005.tif]

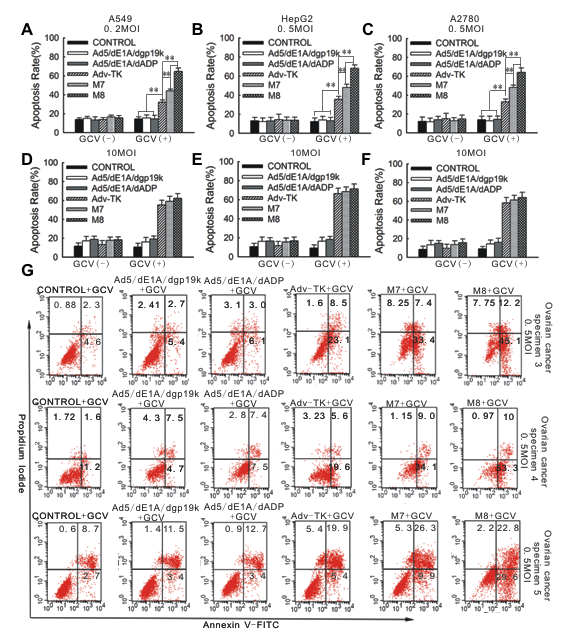

Supplement: Figure S6 — Flow cytometry detected the antitumoral effect of the three virus mutants on tumor cells (A549, HepG2, A2780). Tumor cells (A549, HepG2, A2780) on 12-well plates were infected with various virus mutants at a MOI of 0.2 of A549 (A), 0.5 MOI of HepG2, (B) and 0.5 MOI of A2780 (C) or at a MOI of 10 A549 (D), HepG2 (E) and A2780 (F), and co-administered with or without 100 ug/ml GCV. The cell apoptosis were checked by flow cytometry at indicated time. A representative flow cytometric analysis of the cell apoptosis rate of isolated primary ovarian cancer cells infected with various virus mutants at a MOI of 0.5 and co-administered with 100 ug/ml GCV was showed in (G). (TIF) [file pone.0094050.s006.tif]

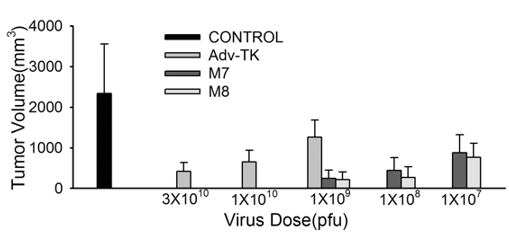

Supplement: Figure S7 — The antitumor effect of variant doses of the three viral mutant in pilot experiment. Variant doses of the three virus mutant were injected into the orthotopic gastric carcinoma xenograft model separately, the tumor volume in each group was measured at the time of study termination. (TIF) [file pone.0094050.s007.tif]

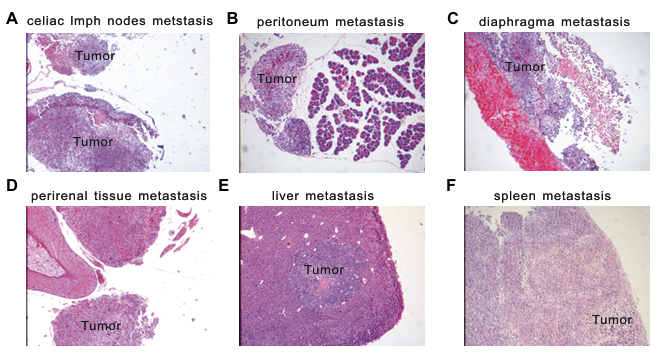

Supplement: Figure S8 — Metastases observed in orthotopic human gastric carcinoma model in each group. (A–F) showed representative histological images of gastric carcinoma in (A). celiac lmph nodes, (B). peritoneum, (C). diaphragma, (D). adrenal glands and kidney ascites, (E). liver, (F). spleen. (TIF) [file pone.0094050.s008.tif]
