# Supplementary material for: Correction: A Systematic Comparison of the Anti-Tumoural Activity and Toxicity of the Three Adv-TKs
Source: PLoS One. 2016 Apr 7;11(4):e0153540. doi: 10.1371/journal.pone.0153540 (PMC4824467; doi:10.1371/journal.pone.0153540)
Supplement: S1 File — (ZIP) [file pone.0153540.s001.zip › S1 File/table about the underlying images.docx]

| File name | Corresponding to the panels in the figure | Underlying image name |
| --- | --- | --- |
| Fig.1 | Fig.1B | Fig.1B |
|  | Fig.1C | Fig.1C (the underlying image was in the box) |
| Fig.2 | Fig.2A left panel | From top to bottom:MKN45 TK Adv-TK |
|  |  | MKN45 actin Adv-TK |
|  |  | MKN45 TK M7 |
|  |  | MKN45 actin M7 |
|  |  | MKN45 TK M8 |
|  |  | MKN45 actin M8 |
|  | Fig.2A right panel | From top to bottom: MCF10A TK Adv-TK |
|  |  | MCF10A actin Adv-TK |
|  |  | MCF10A TK M7 |
|  |  | MCF10A actin M7 |
|  |  | MCF10A TK M8 |
|  |  | MCF10A actin M8 |
|  | Fig.2B left panel | From top to bottom: MKN45 TK |
|  |  | MKN45 actin |
|  | Fig.2B right panel | From top to bottom: MCF10A TK |
|  |  | MCF10A actin |
|  | Fig.2F | 5 day-1 (from columns 1-6 ) |
|  |  | 5 day-2 (from columns 7-8 ) |
|  |  | 7 day-1 (from columns 1-6 ) |
|  |  | 7 day-2 (from columns 7-8 ) |
|  | Fig.2G | MCF 10A-1 (from columns 1-3 ) |
|  |  | MCF 10A-2 (from columns 4-6 ) |
|  |  | MCF 10A-3 (from columns 7-8 ) |
| Fig.3 | Fig.3S | Specimen 1 |
|  |  | Specimen 2 |
| Fig.4 | Fig.4A | CONTROL |
|  |  | Ad5dE1Adgp19K |
|  |  | Ad5dE1AdADP |
|  |  | Adv-TK |
|  |  | M7 |
|  |  | M8 |
| Fig.5 | Fig.5A upper panel | HE: CONTROL |
|  |  | Ad5dE1Adgp19K |
|  |  | Ad5dE1AdADP |
|  |  | Adv-TK |
|  |  | M7 |
|  |  | M8 |

|  | Fig.5A middle panel | In situ: CONTROL |
| --- | --- | --- |
|  |  | Ad5dE1Adgp19K |
|  |  | Ad5dE1AdADP |
|  |  | Adv-TK |
|  |  | M7 |
|  |  | M8 |
|  | Fig.5A lower panel | IHC: CONTROL |
|  |  | Ad5dE1Adgp19K |
|  |  | Ad5dE1AdADP |
|  |  | Adv-TK |
|  |  | M7 |
|  |  | M8 |
|  | Fig.5D | Upper: TK |
|  |  | Lower: actin |
| Fig.6 | Fig.6E | LUNG: CONTROL |
|  |  | GCV |
|  |  | Adv-TK+GCV |
|  |  | M7+GCV |
|  |  | M8+GCV |
|  |  | LIVER: CONTROL |
|  |  | GCV |
|  |  | Adv-TK+GCV |
|  |  | M7+GCV |
|  |  | M8+GCV |
|  |  | KINDEY: CONTROL |
|  |  | GCV |
|  |  | Adv-TK+GCV |
|  |  | M7+GCV |
|  |  | M8+GCV |
|  |  | SPLEEN: CONTROL |
|  |  | GCV |
|  |  | Adv-TK+GCV |
|  |  | M7+GCV |
|  |  | M8+GCV |
| Supplementary Fig.6 | Supplementary Fig.6G | Specimen 3 |
|  |  | Specimen 4 |
|  |  | Specimen 5 |
| Supplementary Fig.8 | Supplementary Fig.8A | S8A: Celiac lmph nodes metastasis |
|  | Supplementary Fig.8B | S8B: Peritoneum metastasis |
|  | Supplementary Fig.8C | S8C: diaphragm metastasis |

|  | Supplementary Fig.8D | S8D: perirenal tissue metastasis |
| --- | --- | --- |
|  | Supplementary Fig.8E | S8E: liver metastasis |
|  | Supplementary Fig.8F | S8F: spleen metastasis |
