# Supplementary figures and images for: Correction: A Systematic Comparison of the Anti-Tumoural Activity and Toxicity of the Three Adv-TKs
Source: PLoS One. 2016 Apr 7;11(4):e0153540. doi: 10.1371/journal.pone.0153540 (PMC4824467; doi:10.1371/journal.pone.0153540)

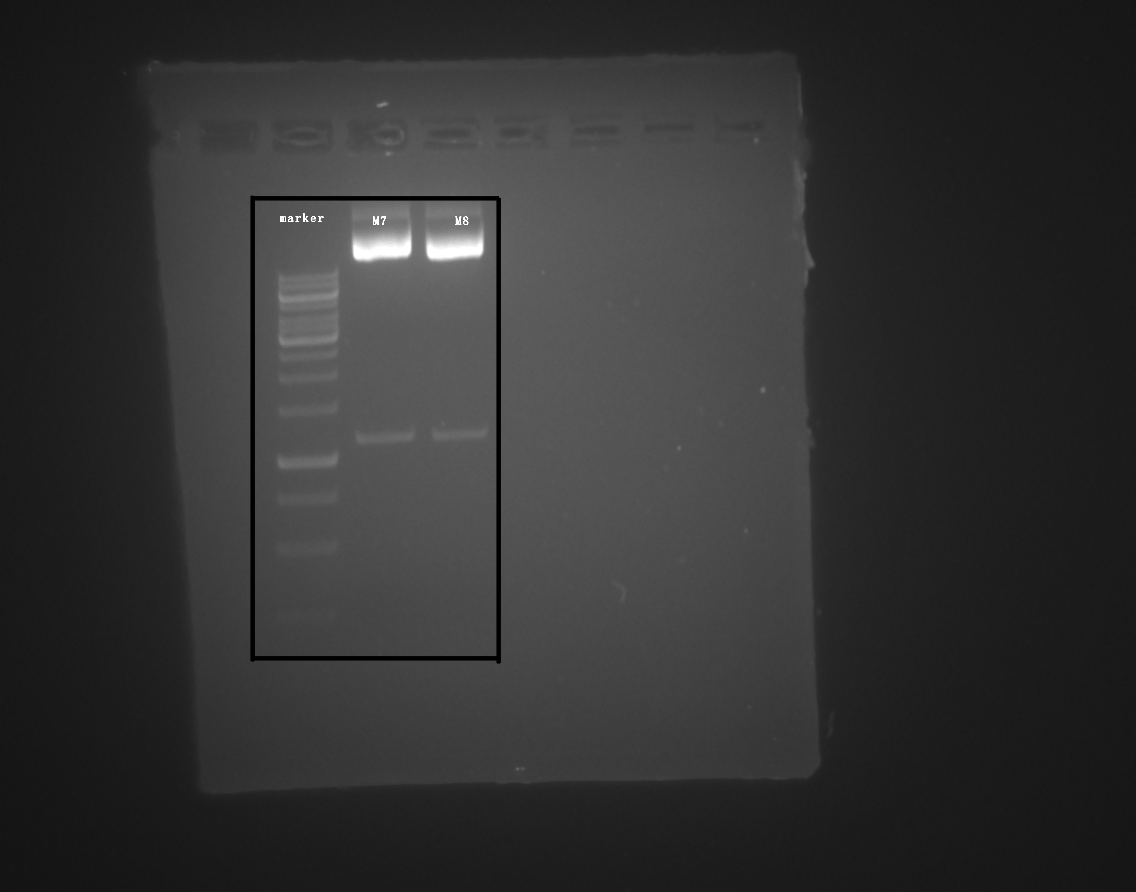

Supplement: S1 File — (ZIP) [file pone.0153540.s001.zip › S1 File/Fig.1/Fig 1B.tif]

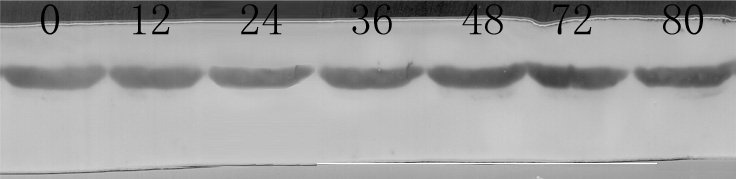

Supplement: S1 File — (ZIP) [file pone.0153540.s001.zip › S1 File/Fig.2/Fig.2A/Fig.2A left panel/MKN45 actin Adv-TK.tif]

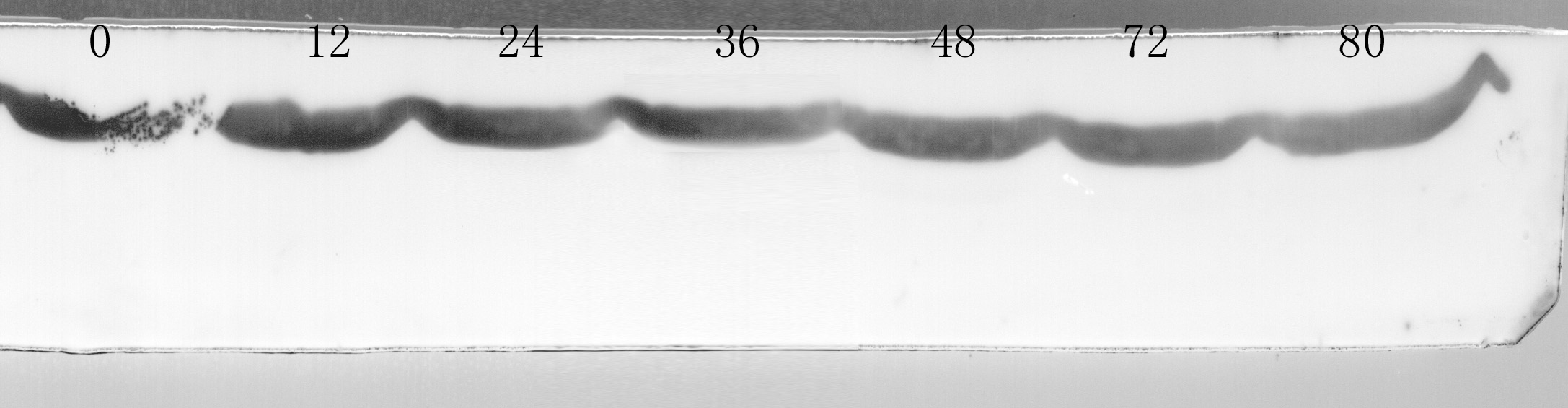

Supplement: S1 File — (ZIP) [file pone.0153540.s001.zip › S1 File/Fig.2/Fig.2A/Fig.2A left panel/MKN45 actin M7.tif]

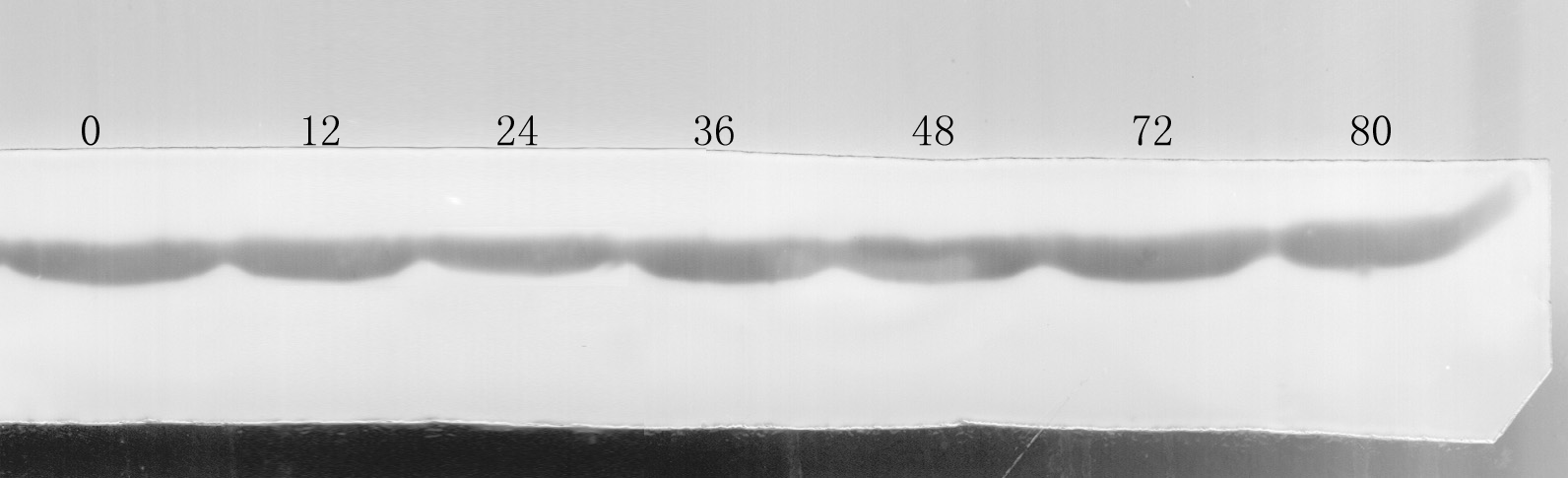

Supplement: S1 File — (ZIP) [file pone.0153540.s001.zip › S1 File/Fig.2/Fig.2A/Fig.2A left panel/MKN45 actin M8.tif]

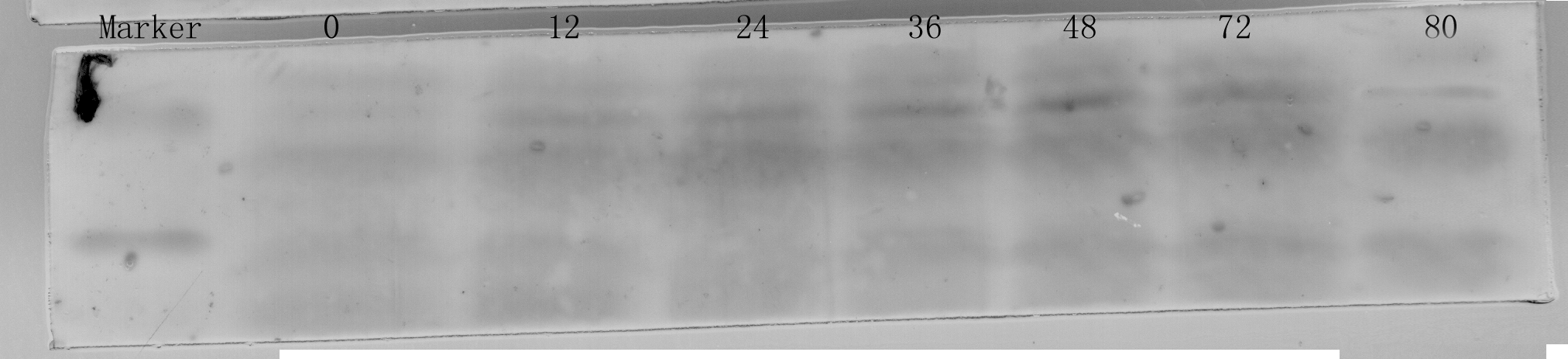

Supplement: S1 File — (ZIP) [file pone.0153540.s001.zip › S1 File/Fig.2/Fig.2A/Fig.2A left panel/MKN45 TK Adv-TK.tif]

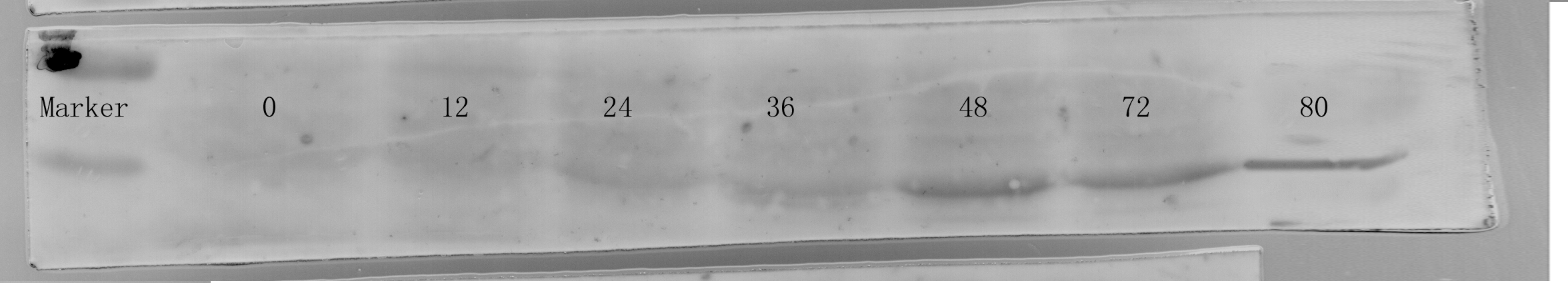

Supplement: S1 File — (ZIP) [file pone.0153540.s001.zip › S1 File/Fig.2/Fig.2A/Fig.2A left panel/MKN45 TK M7.tif]

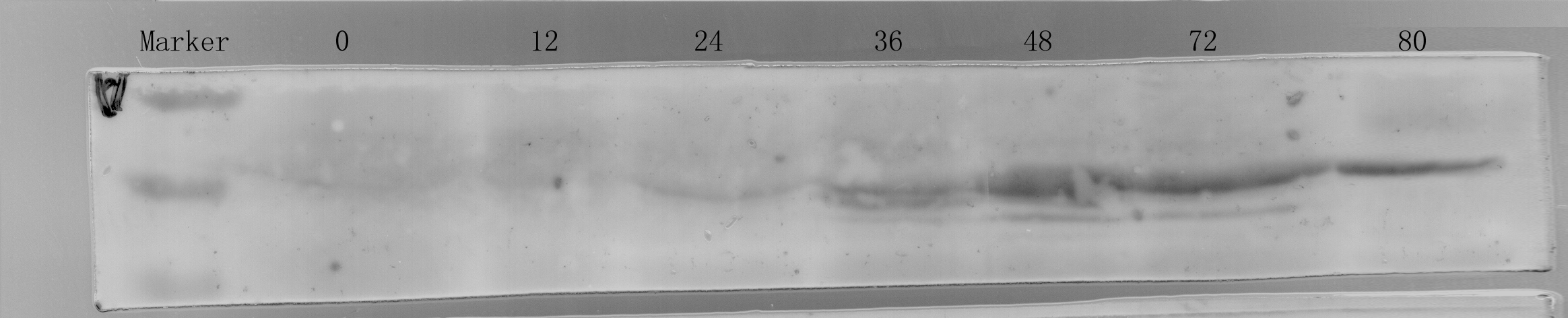

Supplement: S1 File — (ZIP) [file pone.0153540.s001.zip › S1 File/Fig.2/Fig.2A/Fig.2A left panel/MKN45 TK M8.tif]

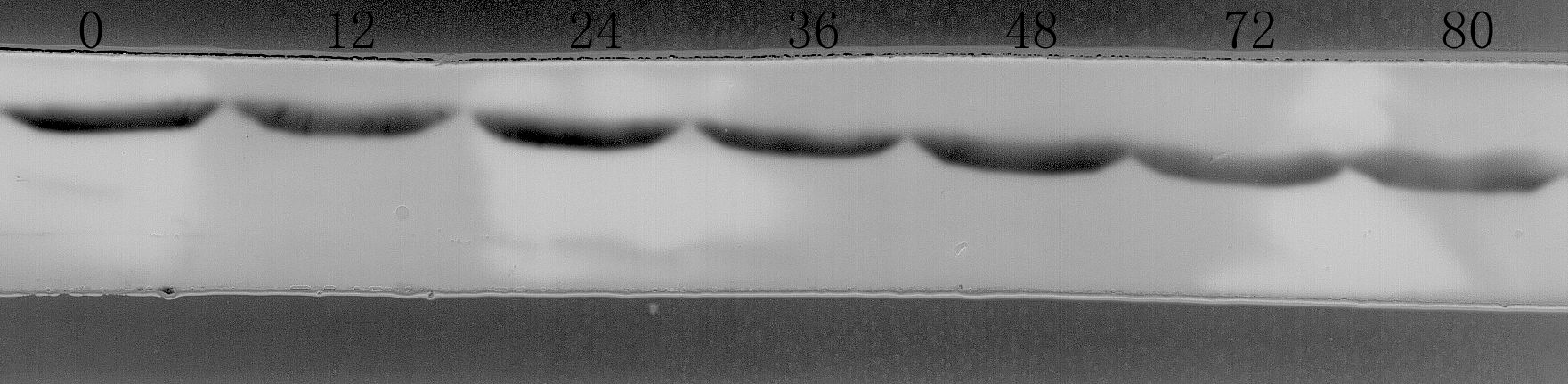

Supplement: S1 File — (ZIP) [file pone.0153540.s001.zip › S1 File/Fig.2/Fig.2A/Fig.2A right panel/MCF10A actin ADV-TK.tif]

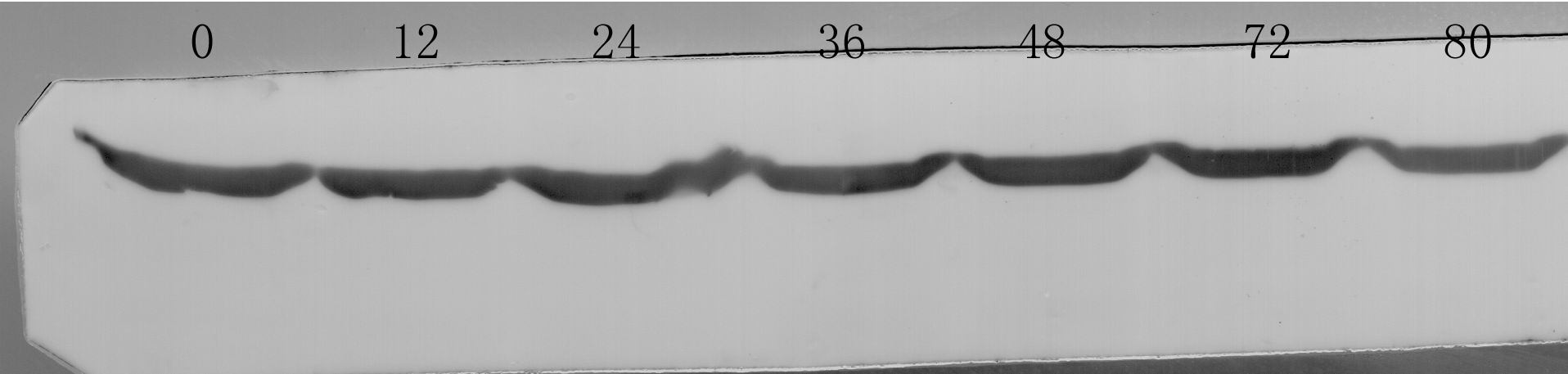

Supplement: S1 File — (ZIP) [file pone.0153540.s001.zip › S1 File/Fig.2/Fig.2A/Fig.2A right panel/MCF10A actin M7.tif]

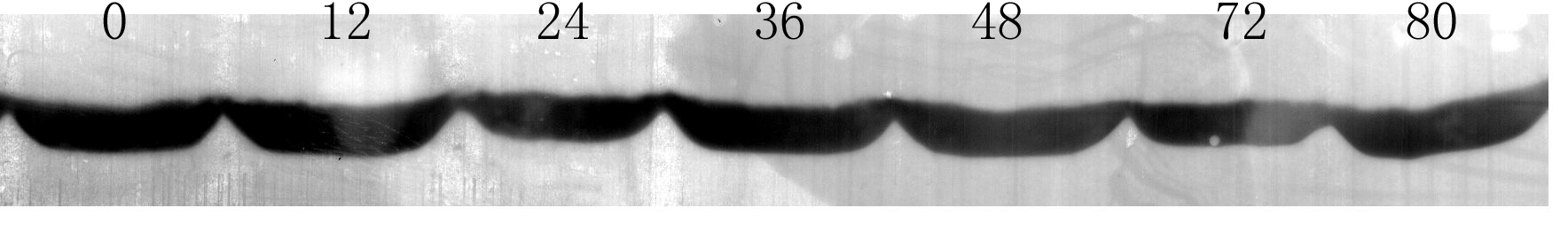

Supplement: S1 File — (ZIP) [file pone.0153540.s001.zip › S1 File/Fig.2/Fig.2A/Fig.2A right panel/MCF10A actin M8.tif]

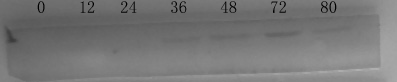

Supplement: S1 File — (ZIP) [file pone.0153540.s001.zip › S1 File/Fig.2/Fig.2A/Fig.2A right panel/MCF10A TK Adv-TK.tif]

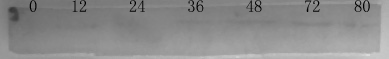

Supplement: S1 File — (ZIP) [file pone.0153540.s001.zip › S1 File/Fig.2/Fig.2A/Fig.2A right panel/MCF10A TK M7.tif]

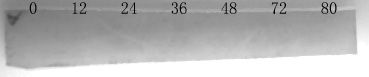

Supplement: S1 File — (ZIP) [file pone.0153540.s001.zip › S1 File/Fig.2/Fig.2A/Fig.2A right panel/MCF10A TK M8.tif]

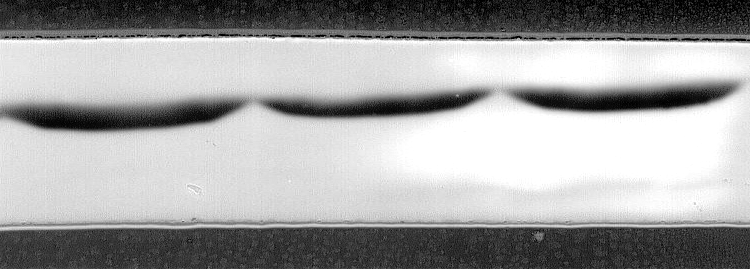

Supplement: S1 File — (ZIP) [file pone.0153540.s001.zip › S1 File/Fig.2/Fig.2B/Fig.2B left panel/MKN45 actin.tif]

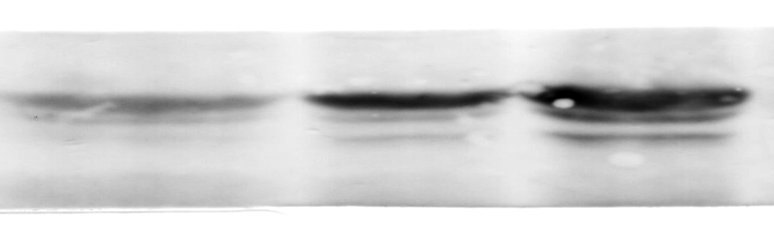

Supplement: S1 File — (ZIP) [file pone.0153540.s001.zip › S1 File/Fig.2/Fig.2B/Fig.2B left panel/MKN45 TK.tif]

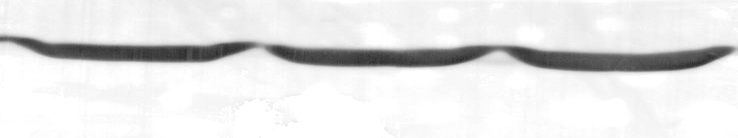

Supplement: S1 File — (ZIP) [file pone.0153540.s001.zip › S1 File/Fig.2/Fig.2B/Fig.2B right panel/MCF-10A actin.tif]

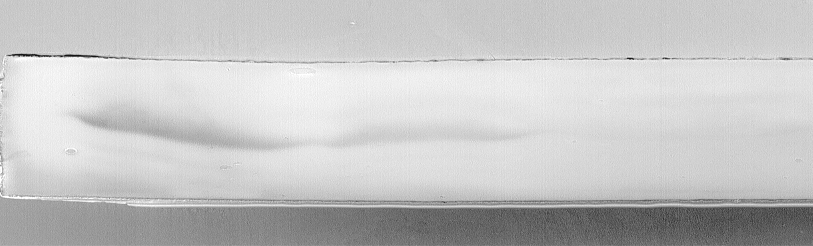

Supplement: S1 File — (ZIP) [file pone.0153540.s001.zip › S1 File/Fig.2/Fig.2B/Fig.2B right panel/MCF-10A TK.tif]

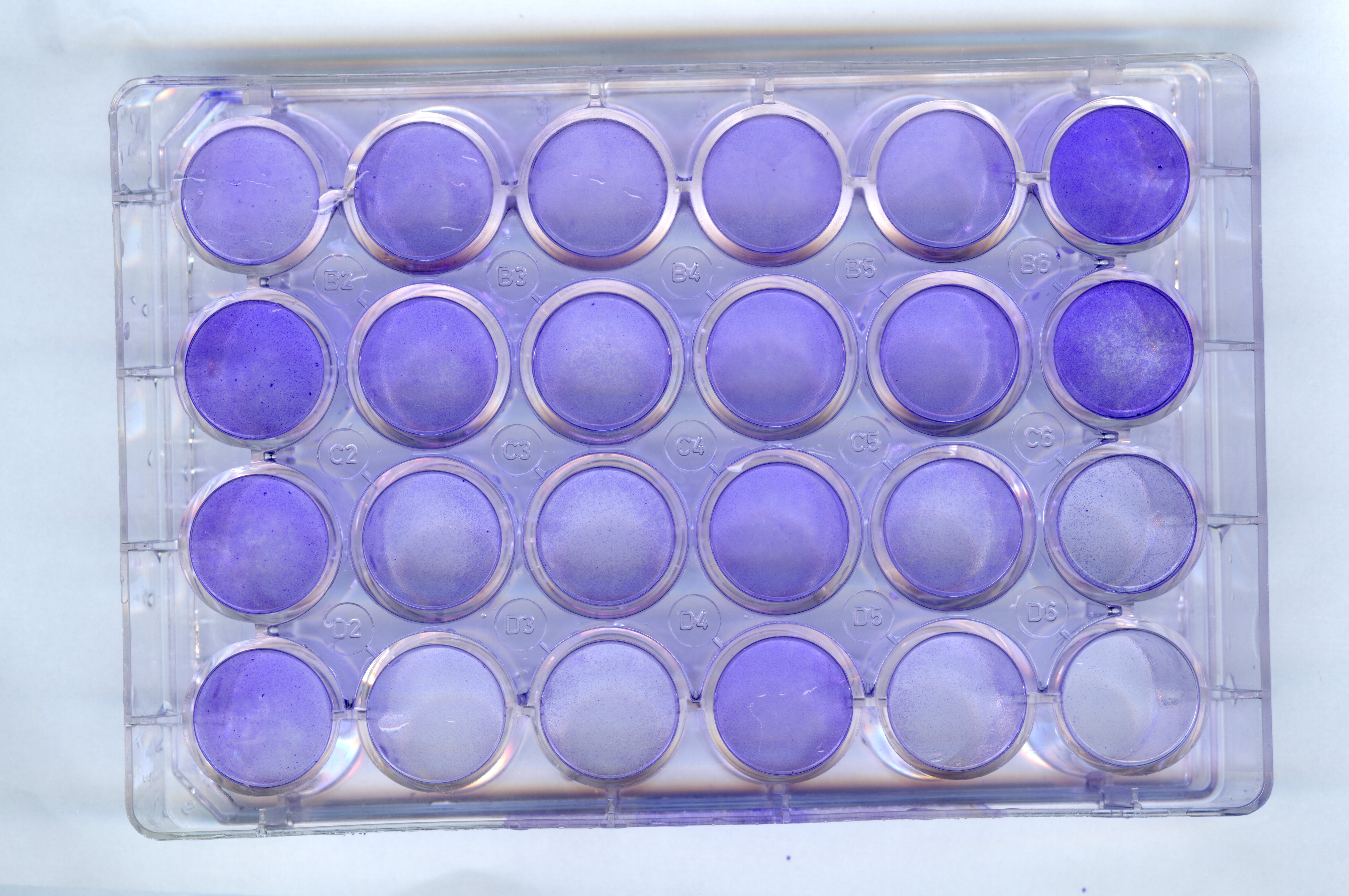

Supplement: S1 File — (ZIP) [file pone.0153540.s001.zip › S1 File/Fig.2/Fig.2F/5 day-1.jpg]

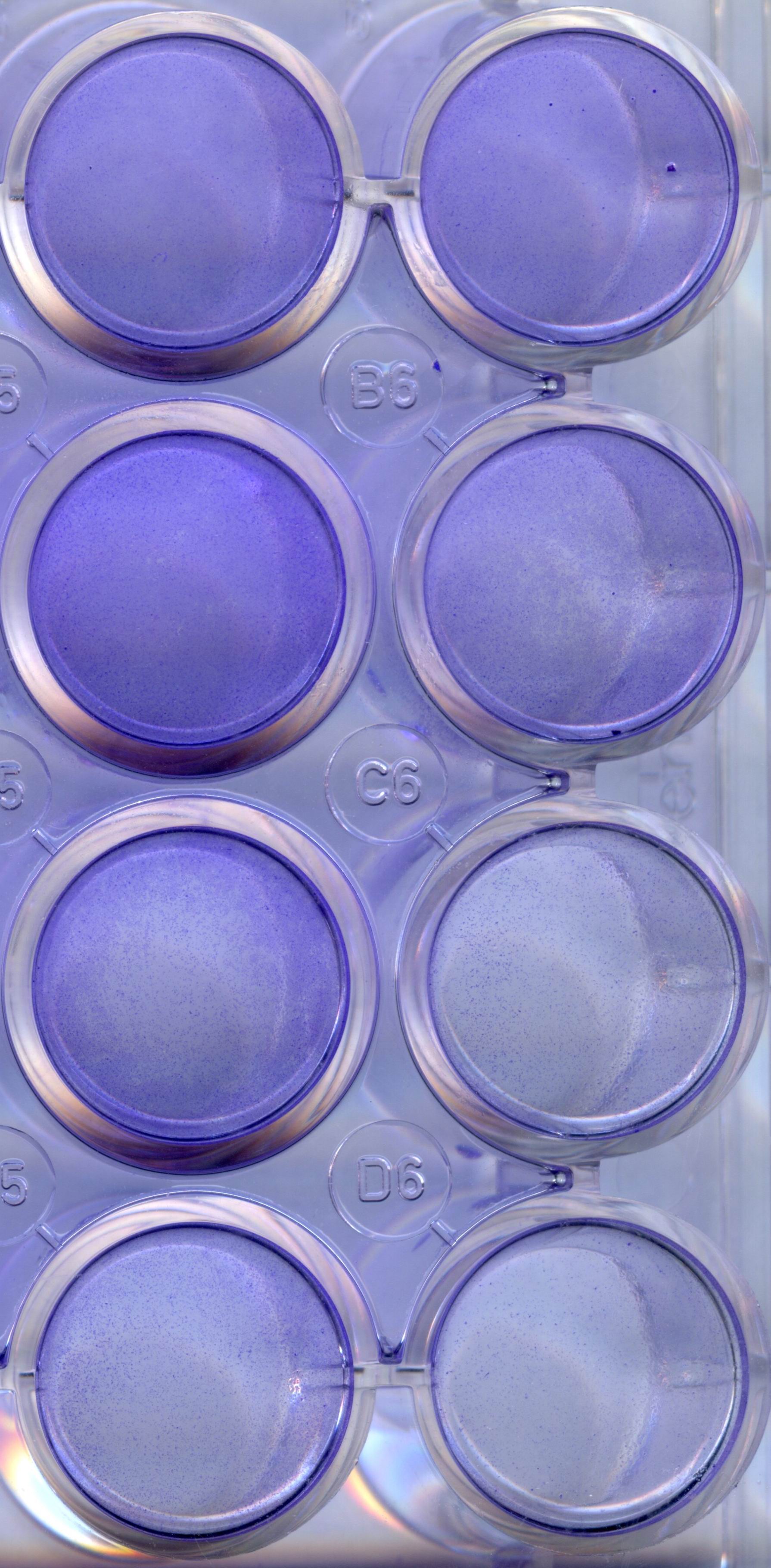

Supplement: S1 File — (ZIP) [file pone.0153540.s001.zip › S1 File/Fig.2/Fig.2F/5 day-2.jpg]

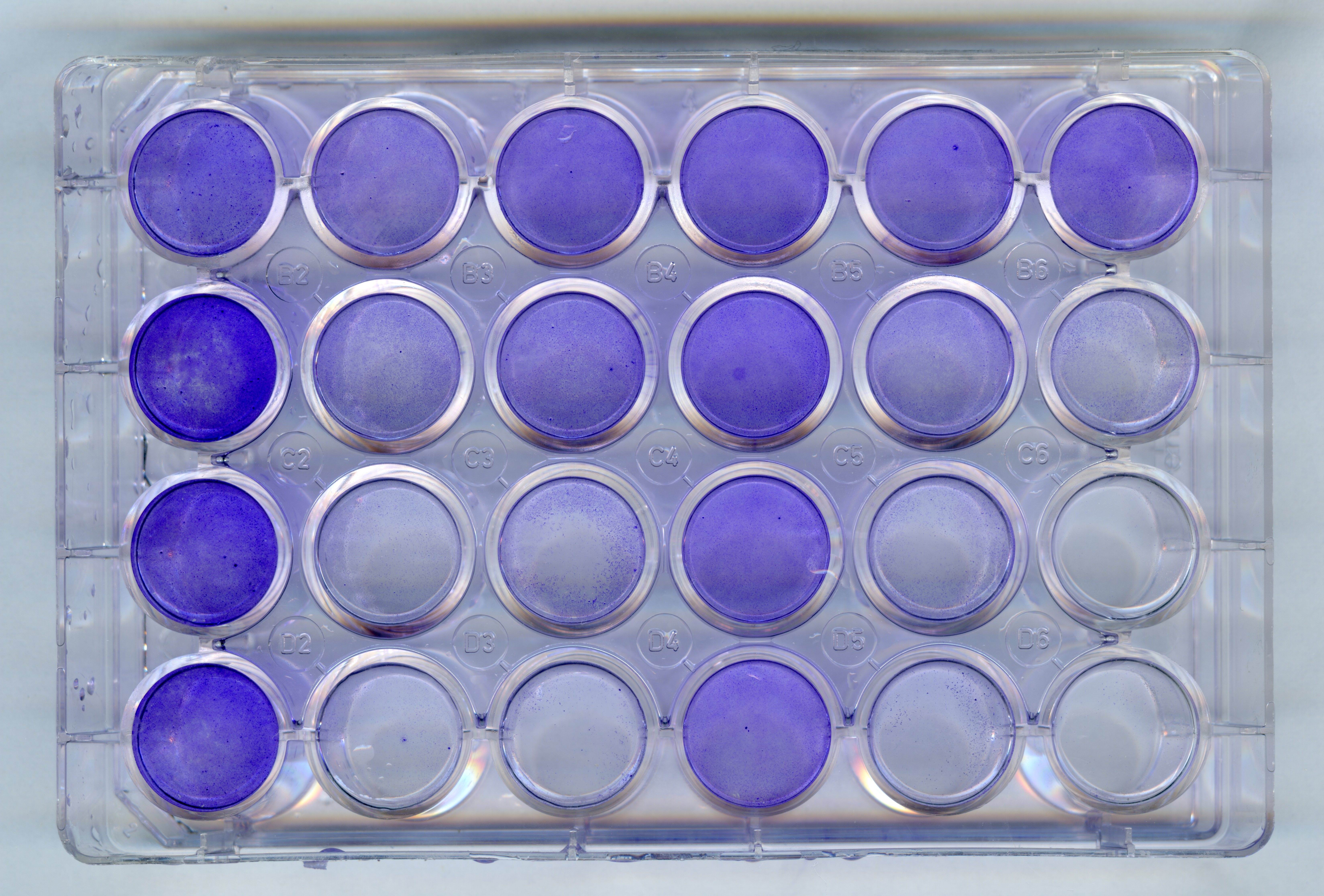

Supplement: S1 File — (ZIP) [file pone.0153540.s001.zip › S1 File/Fig.2/Fig.2F/7 day-1.jpg]

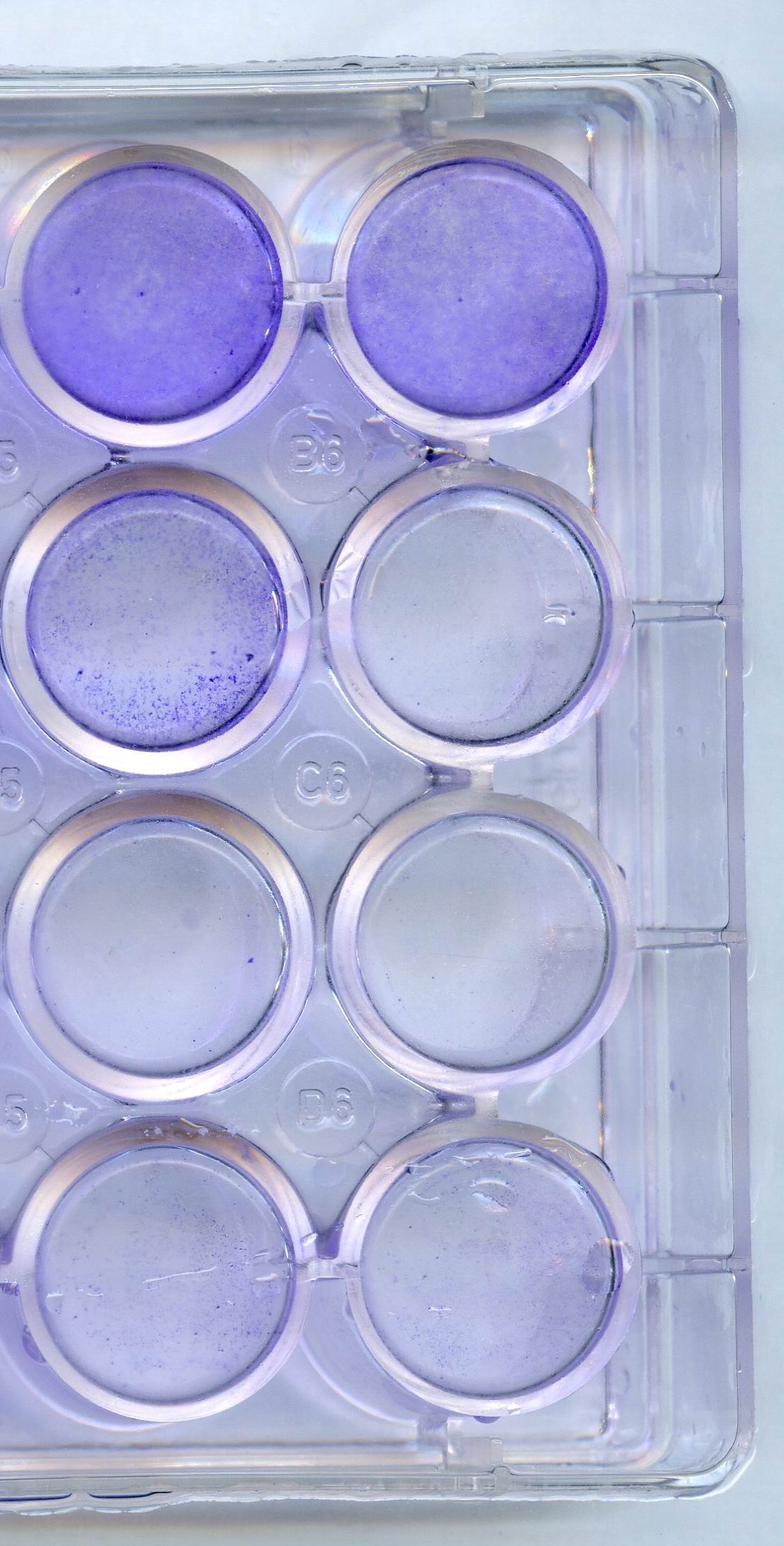

Supplement: S1 File — (ZIP) [file pone.0153540.s001.zip › S1 File/Fig.2/Fig.2F/7 day-2.jpg]

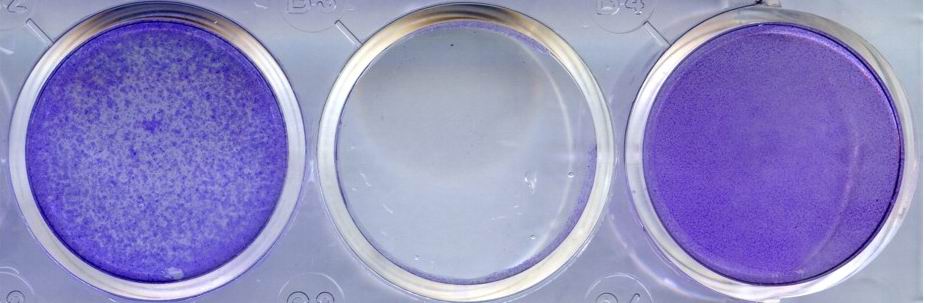

Supplement: S1 File — (ZIP) [file pone.0153540.s001.zip › S1 File/Fig.2/Fig.2G/MCF10A-1.jpg]

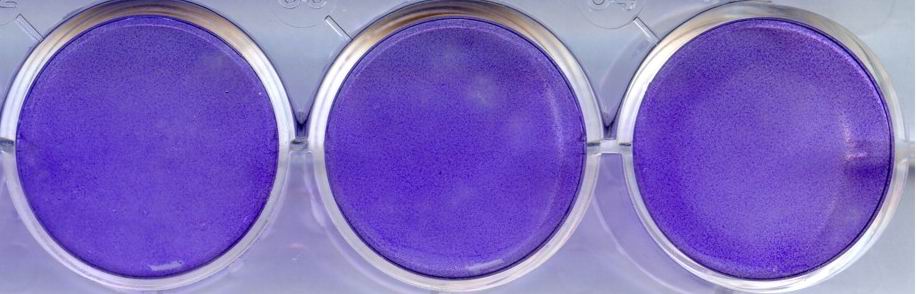

Supplement: S1 File — (ZIP) [file pone.0153540.s001.zip › S1 File/Fig.2/Fig.2G/MCF10A-2.jpg]

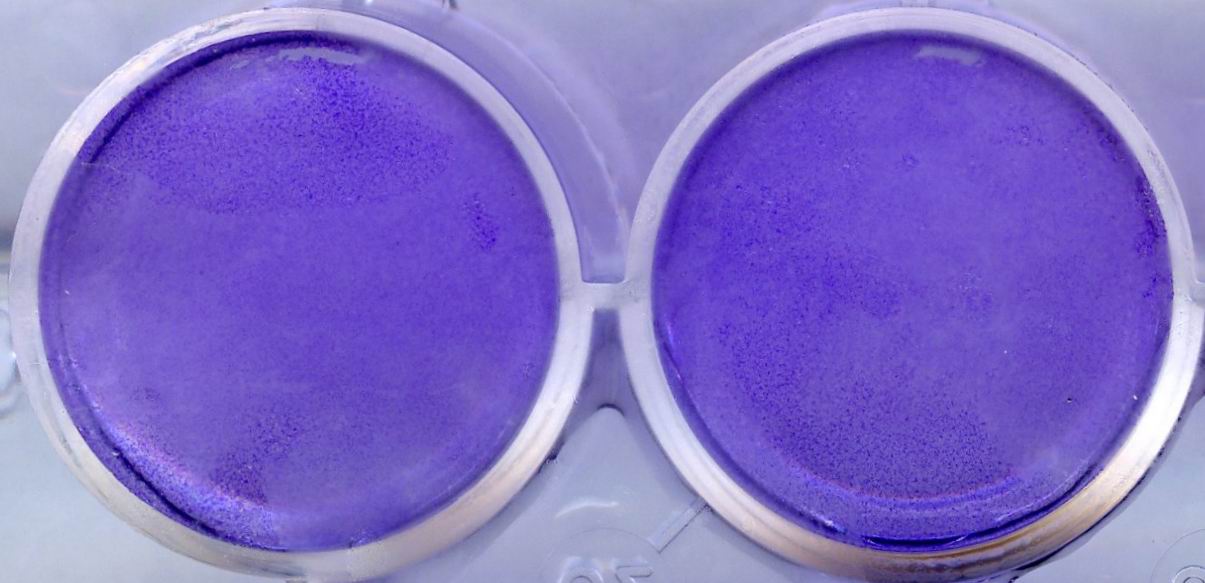

Supplement: S1 File — (ZIP) [file pone.0153540.s001.zip › S1 File/Fig.2/Fig.2G/MCF10A-3.jpg]

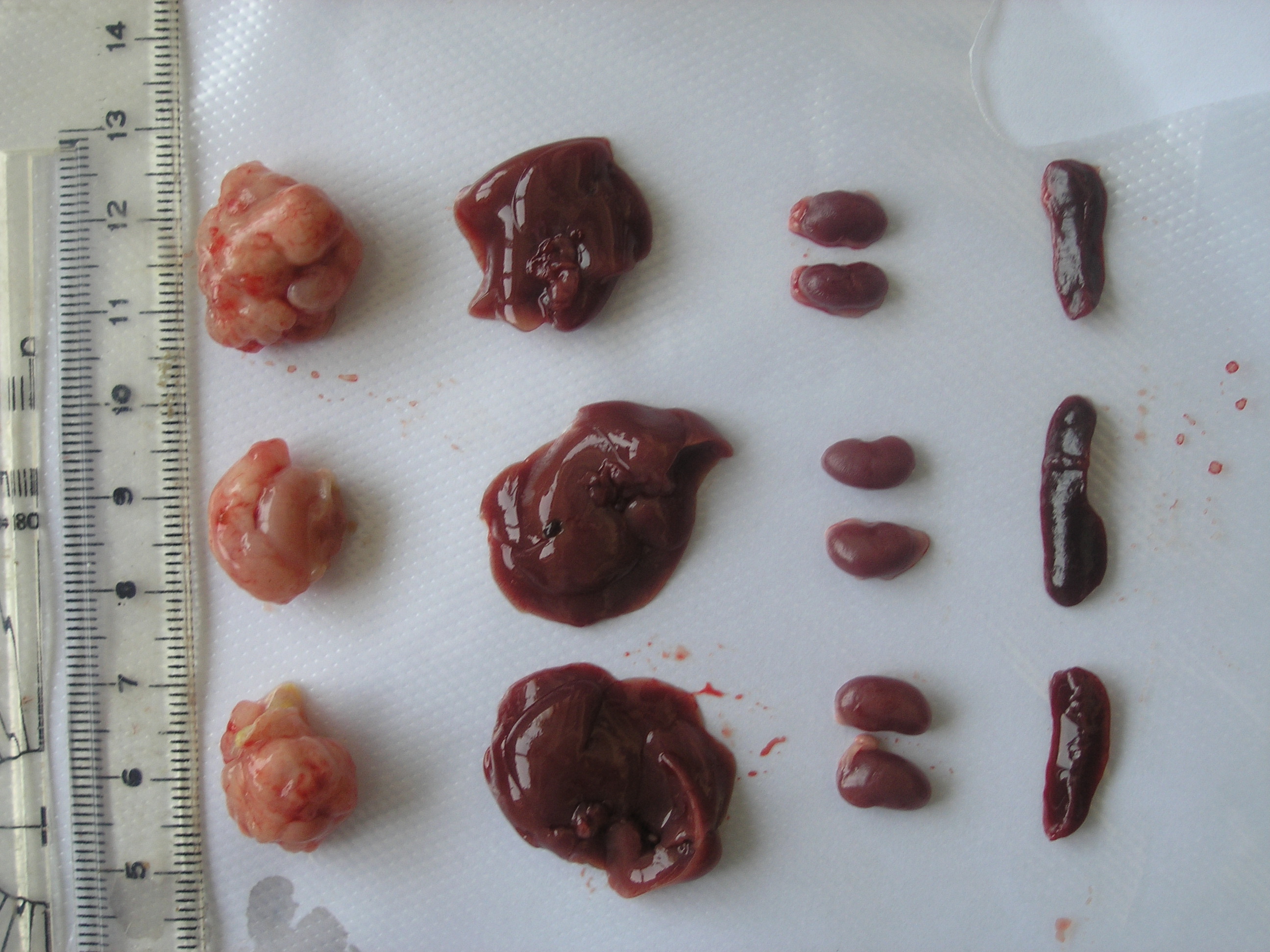

Supplement: S2 File — (ZIP) [file pone.0153540.s002.zip › S2 File/Fig.4A/Ad5dE1AdADP.JPG]

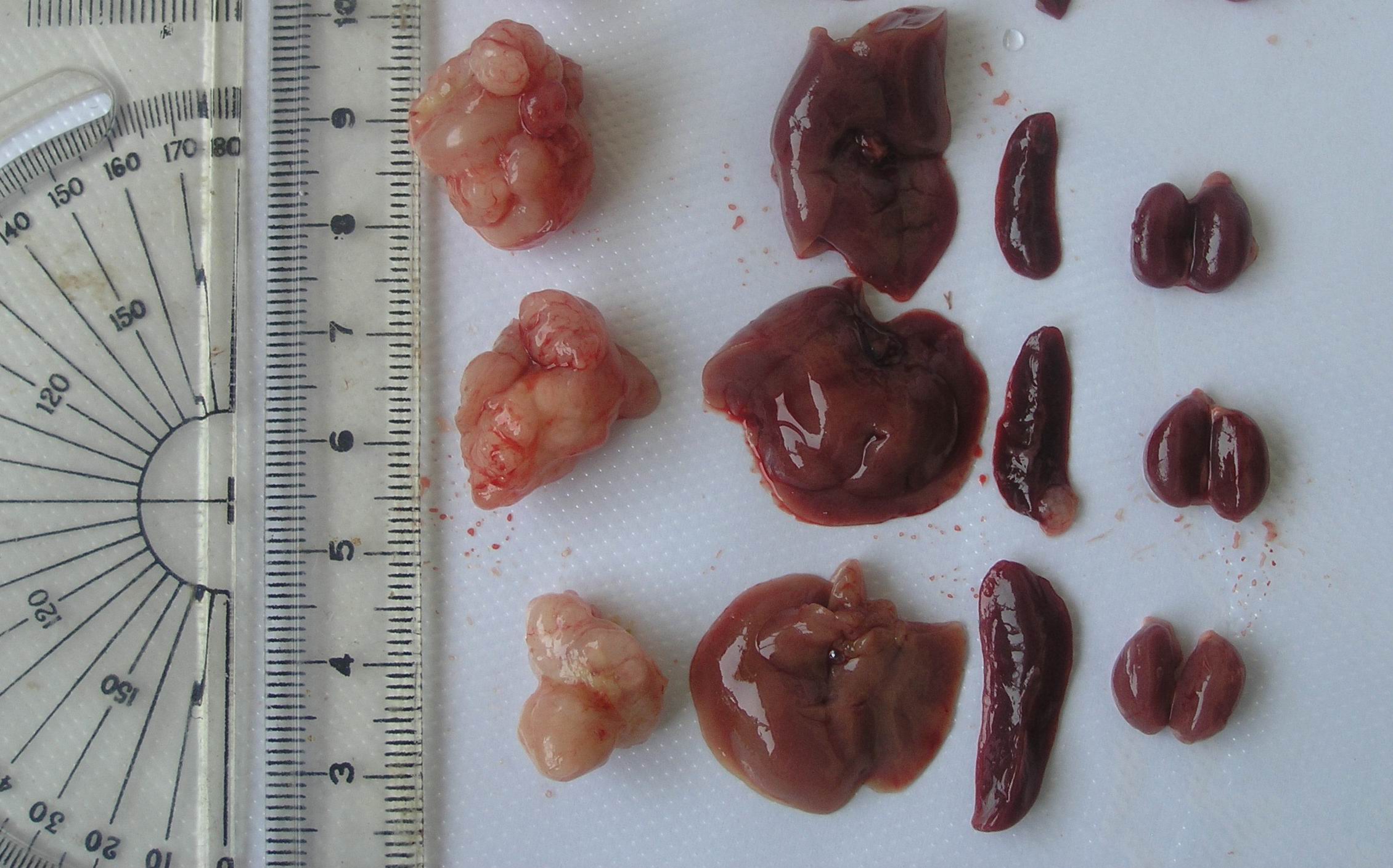

Supplement: S2 File — (ZIP) [file pone.0153540.s002.zip › S2 File/Fig.4A/Ad5dE1Adgp19k.jpg]

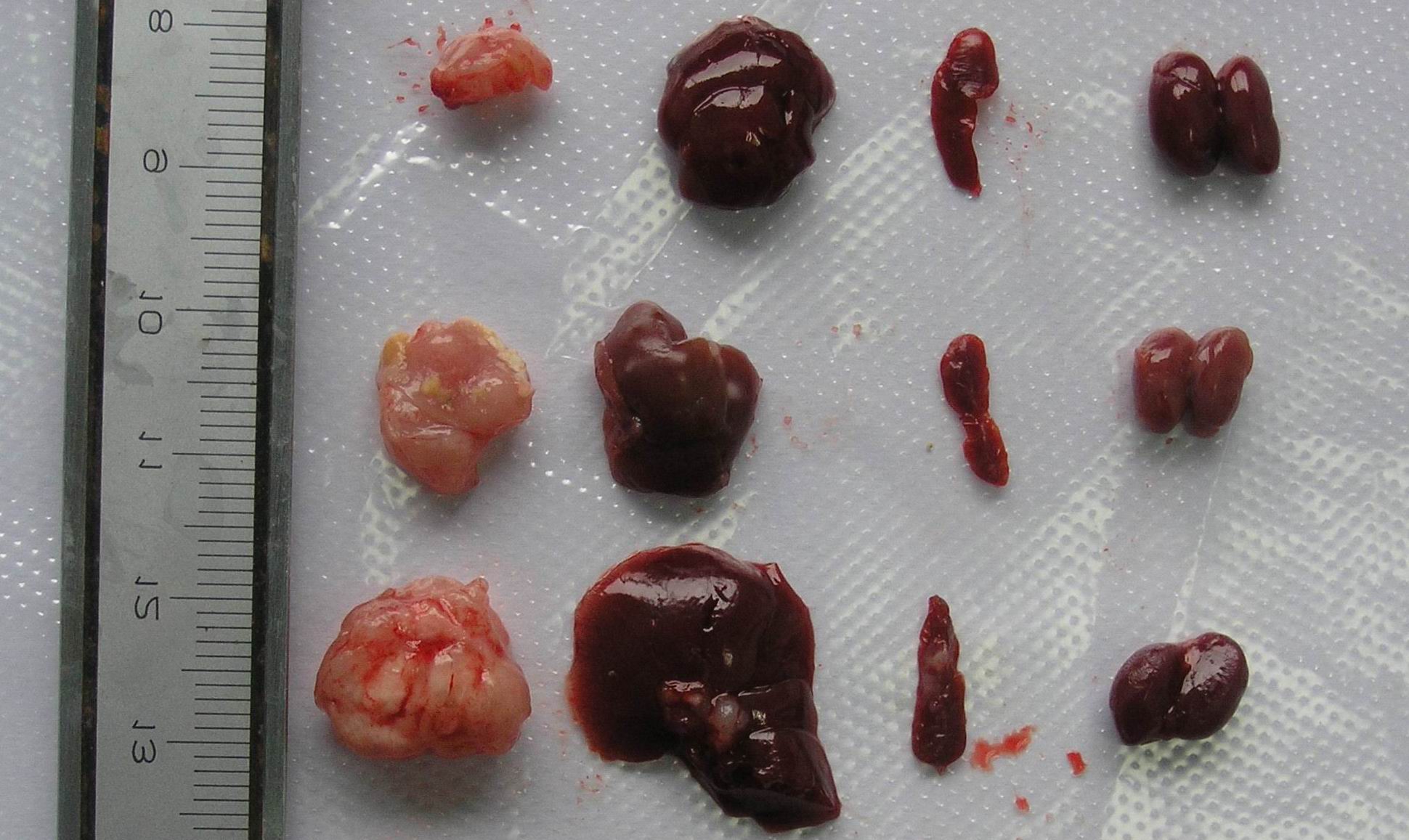

Supplement: S2 File — (ZIP) [file pone.0153540.s002.zip › S2 File/Fig.4A/Adv-TK.jpg]

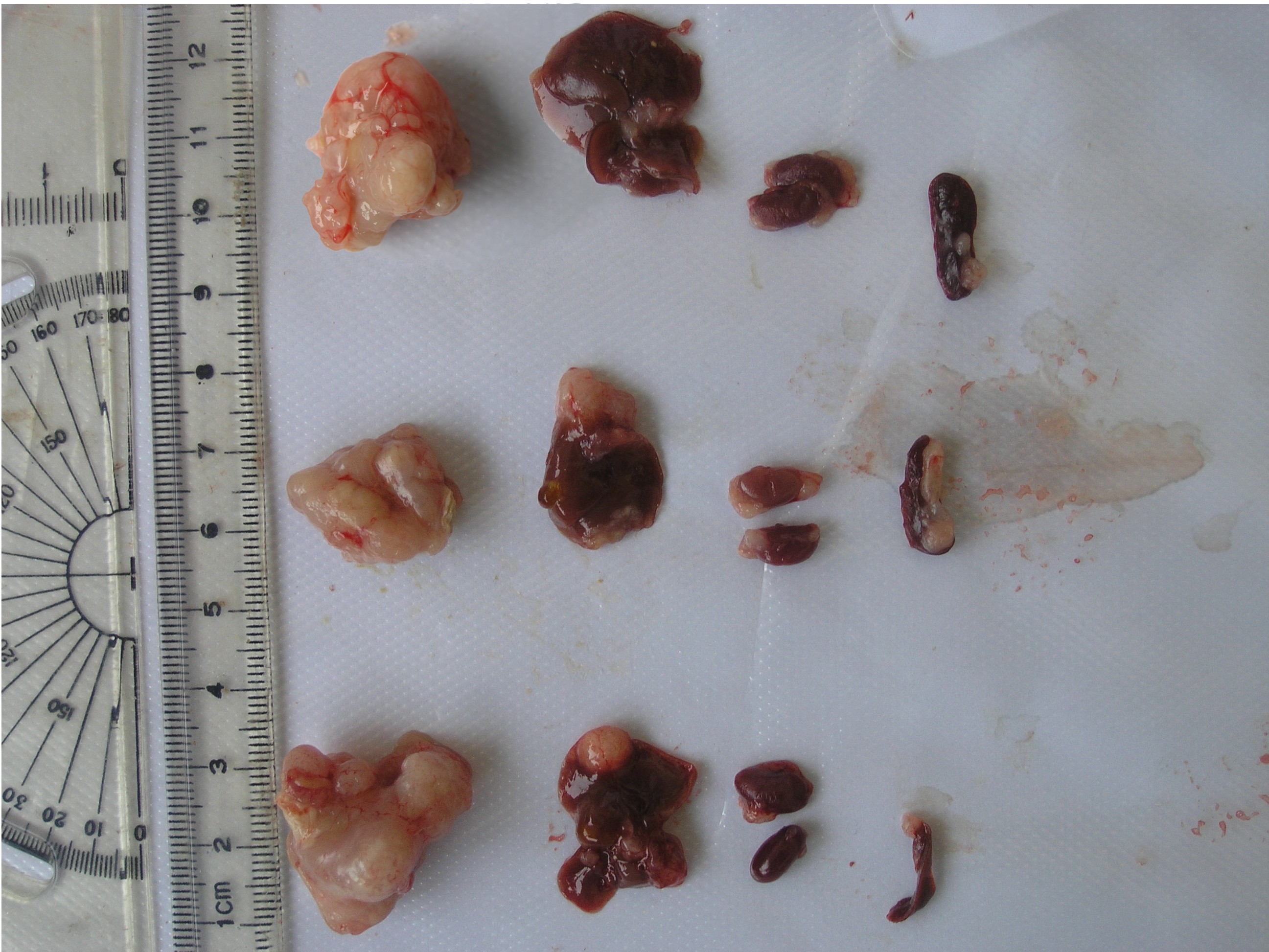

Supplement: S2 File — (ZIP) [file pone.0153540.s002.zip › S2 File/Fig.4A/CONTROL.jpg]

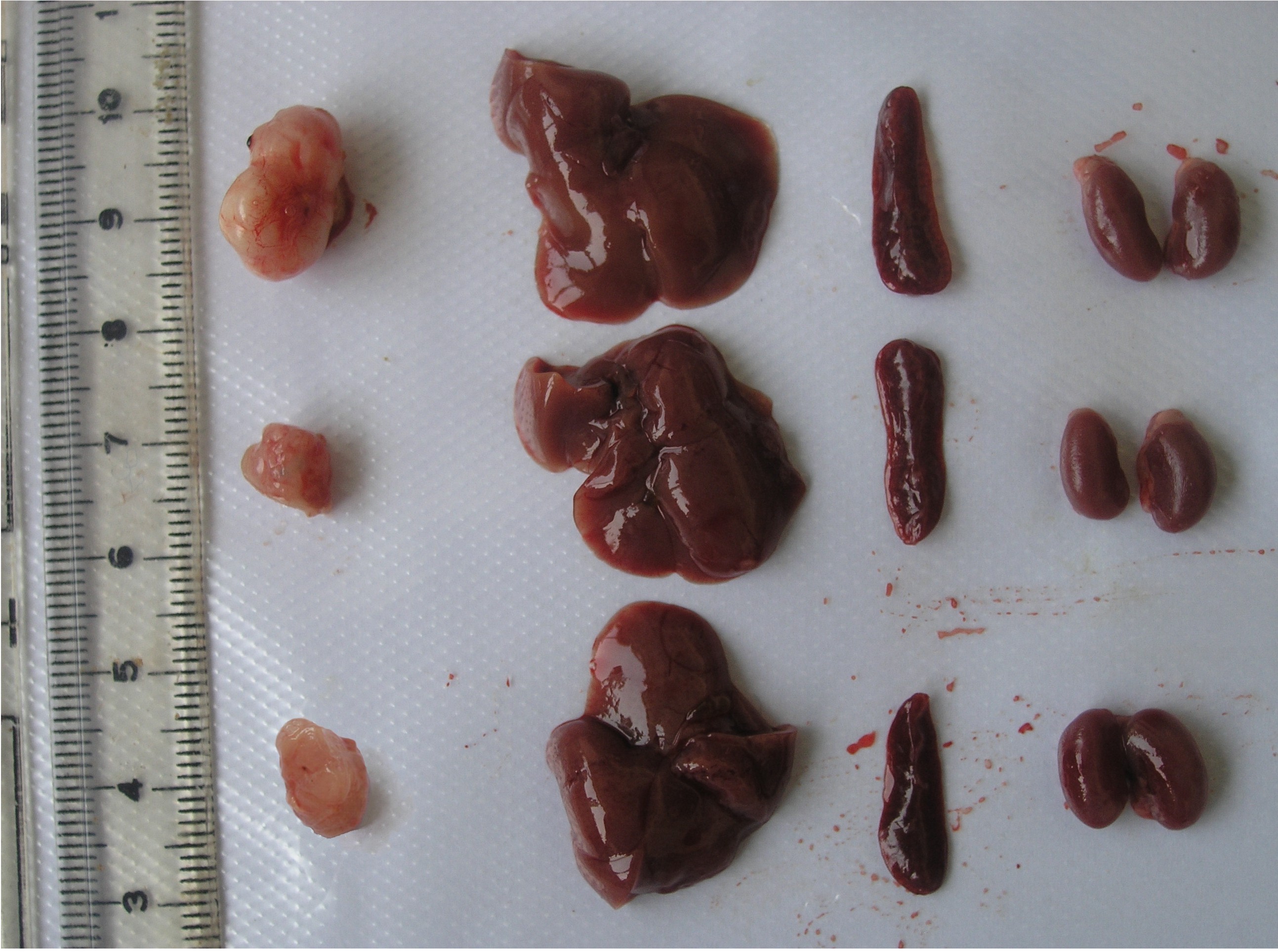

Supplement: S2 File — (ZIP) [file pone.0153540.s002.zip › S2 File/Fig.4A/M7.jpg]

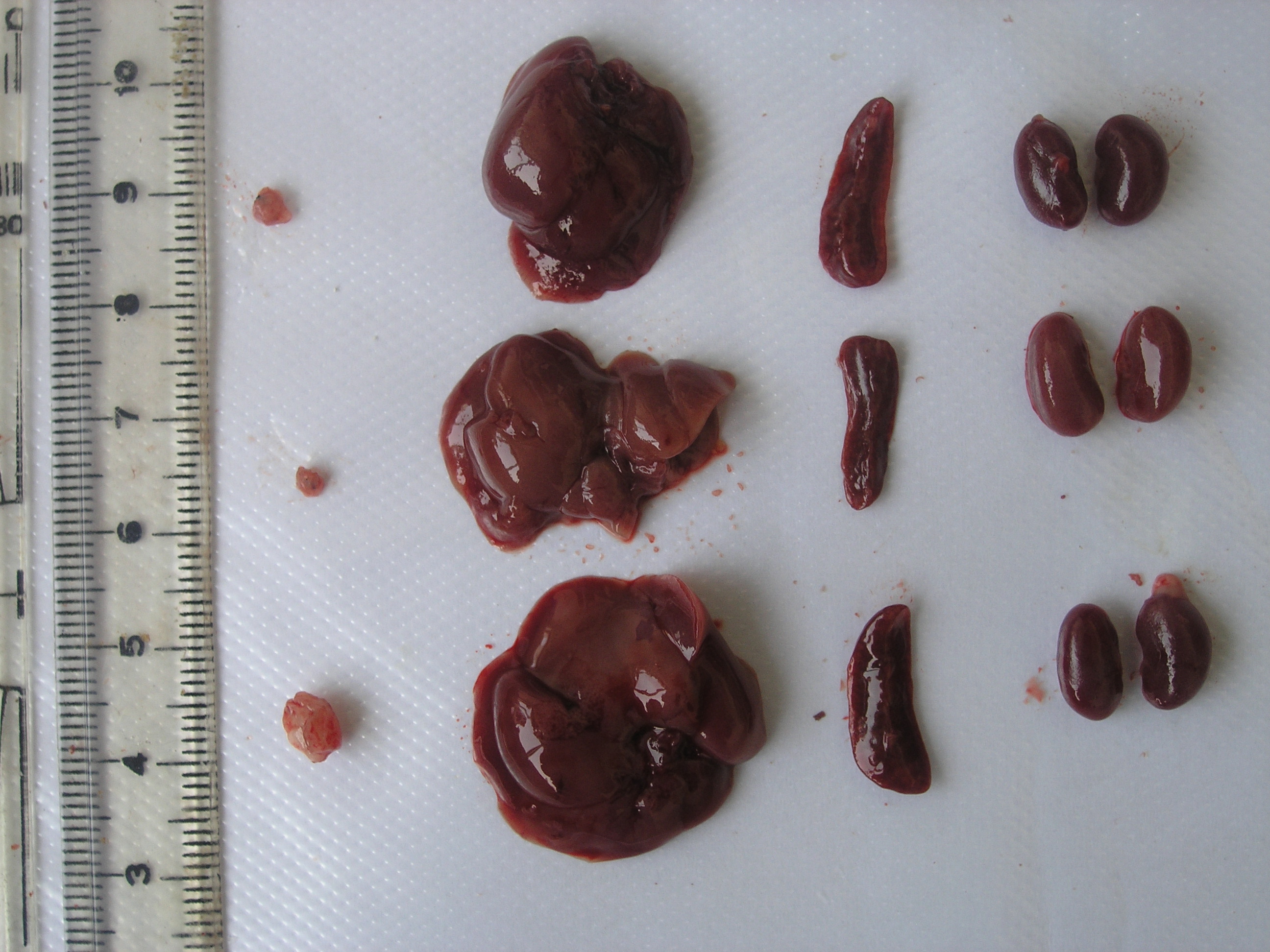

Supplement: S2 File — (ZIP) [file pone.0153540.s002.zip › S2 File/Fig.4A/M8.JPG]

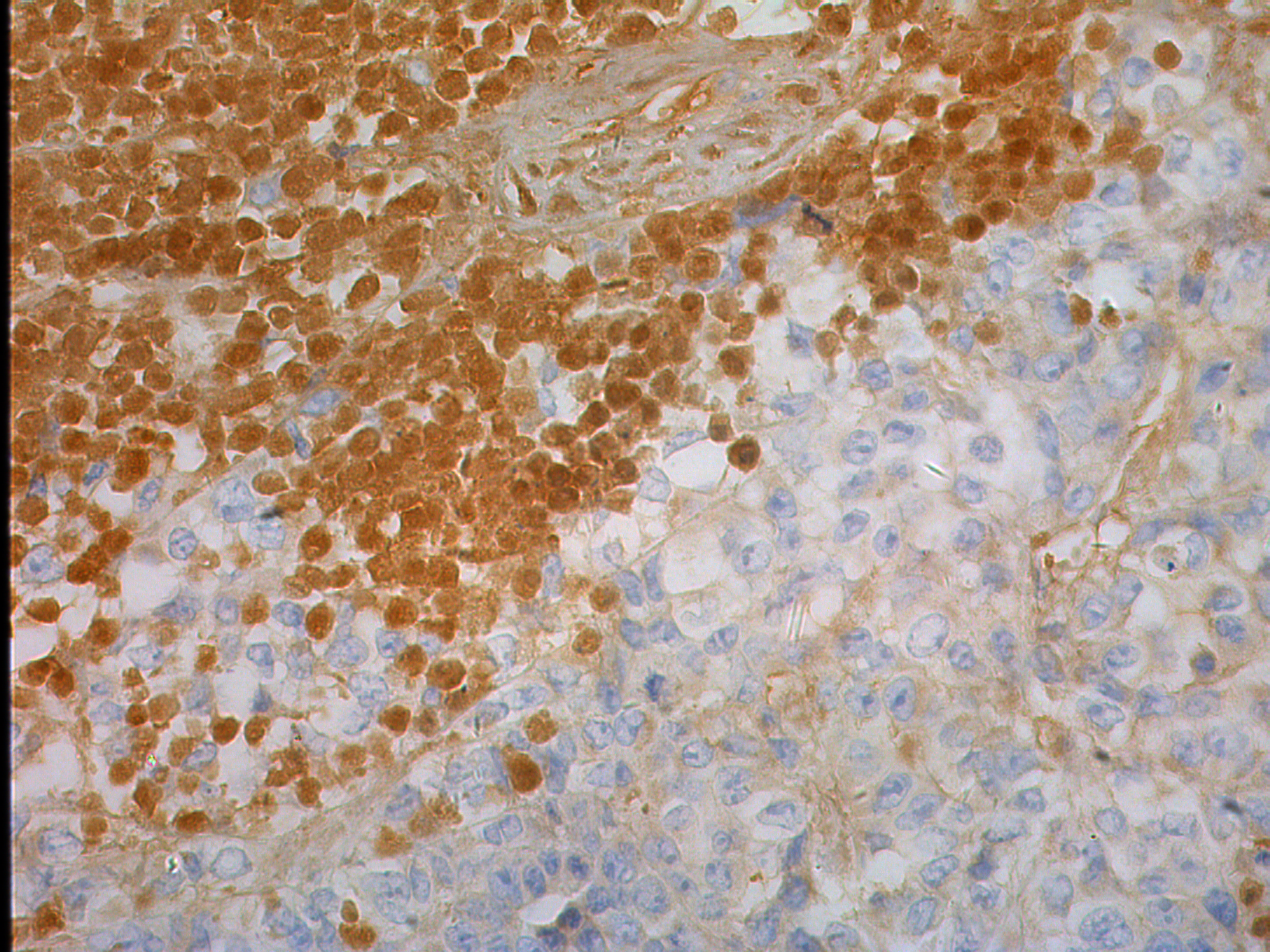

Supplement: S2 File — (ZIP) [file pone.0153540.s002.zip › S2 File/Fig.5/Fig.5A/Fig.5A lower panel IHC/Ad5dE1AdADP.tif]

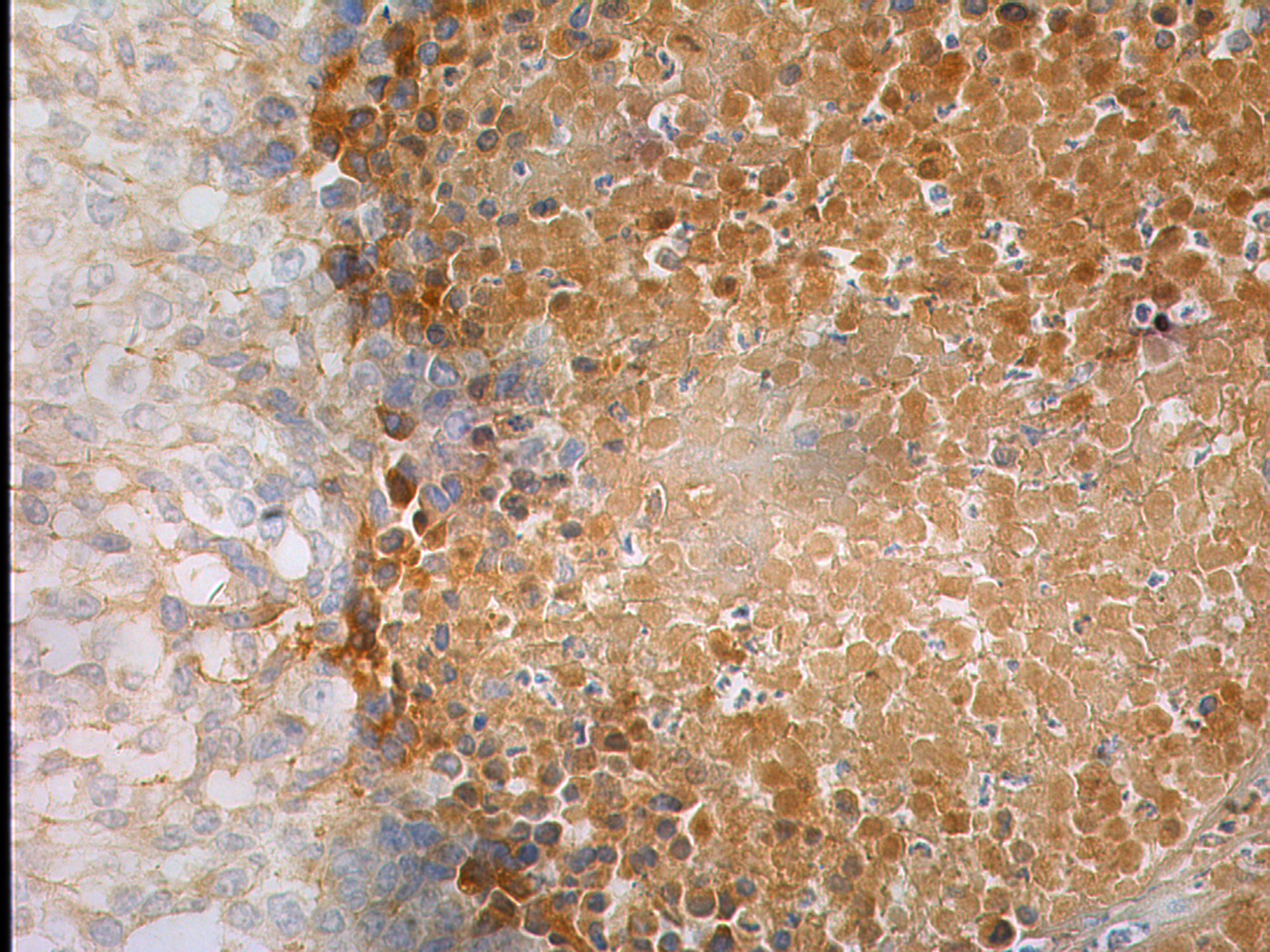

Supplement: S2 File — (ZIP) [file pone.0153540.s002.zip › S2 File/Fig.5/Fig.5A/Fig.5A lower panel IHC/Ad5dE1Adgp19k.tif]

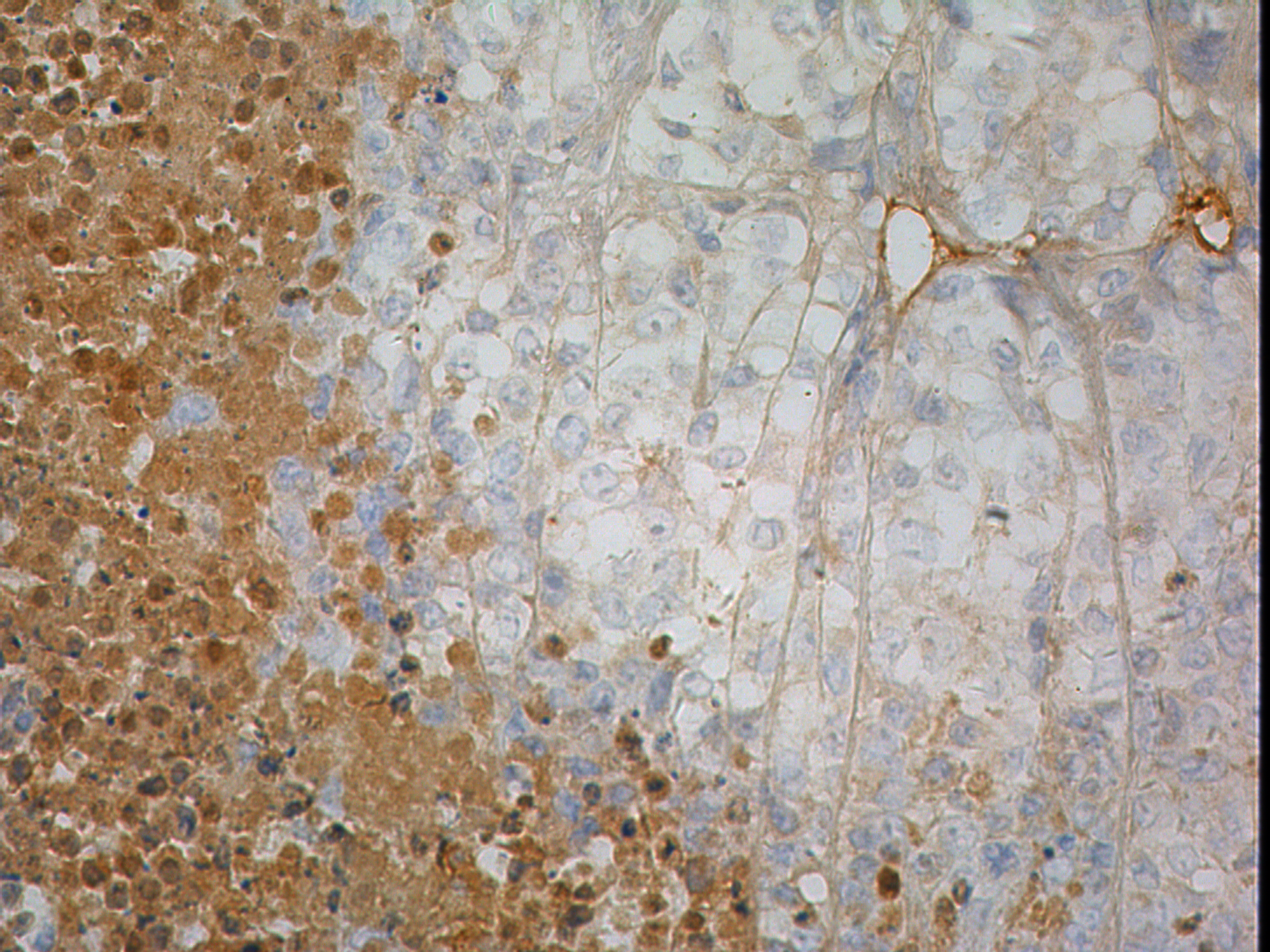

Supplement: S2 File — (ZIP) [file pone.0153540.s002.zip › S2 File/Fig.5/Fig.5A/Fig.5A lower panel IHC/Adv-TK.tif]

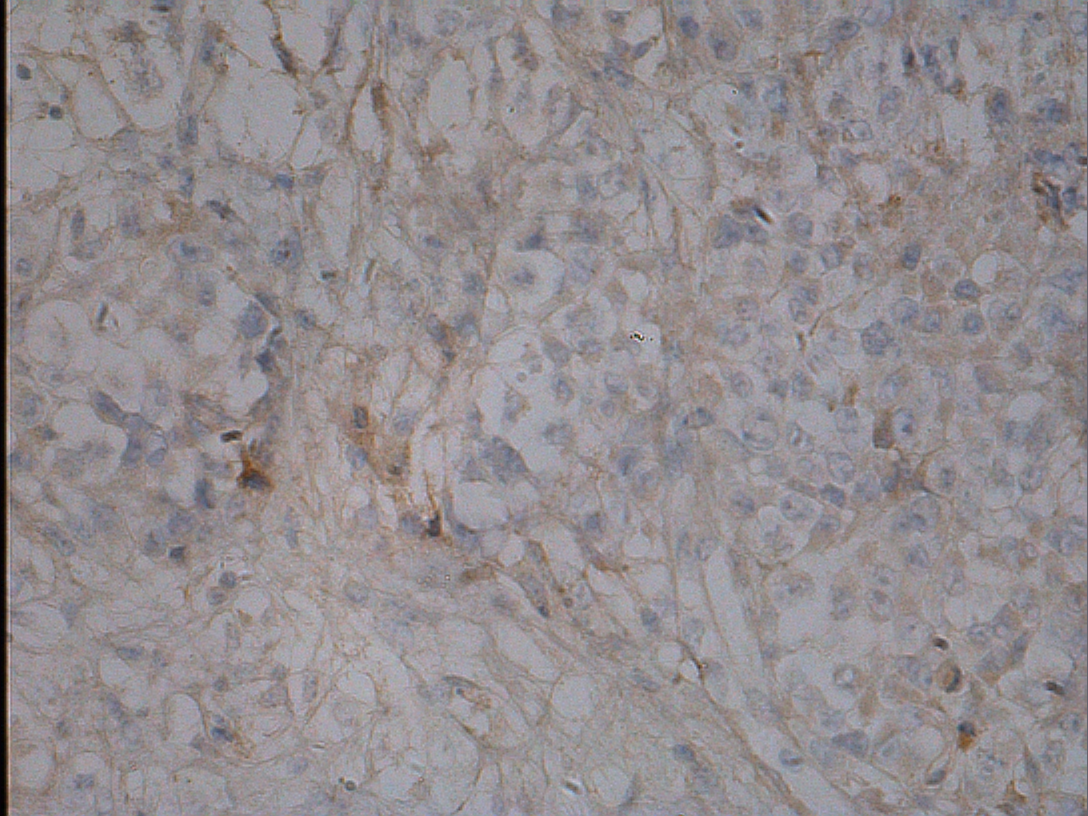

Supplement: S2 File — (ZIP) [file pone.0153540.s002.zip › S2 File/Fig.5/Fig.5A/Fig.5A lower panel IHC/CONTROL.tif]

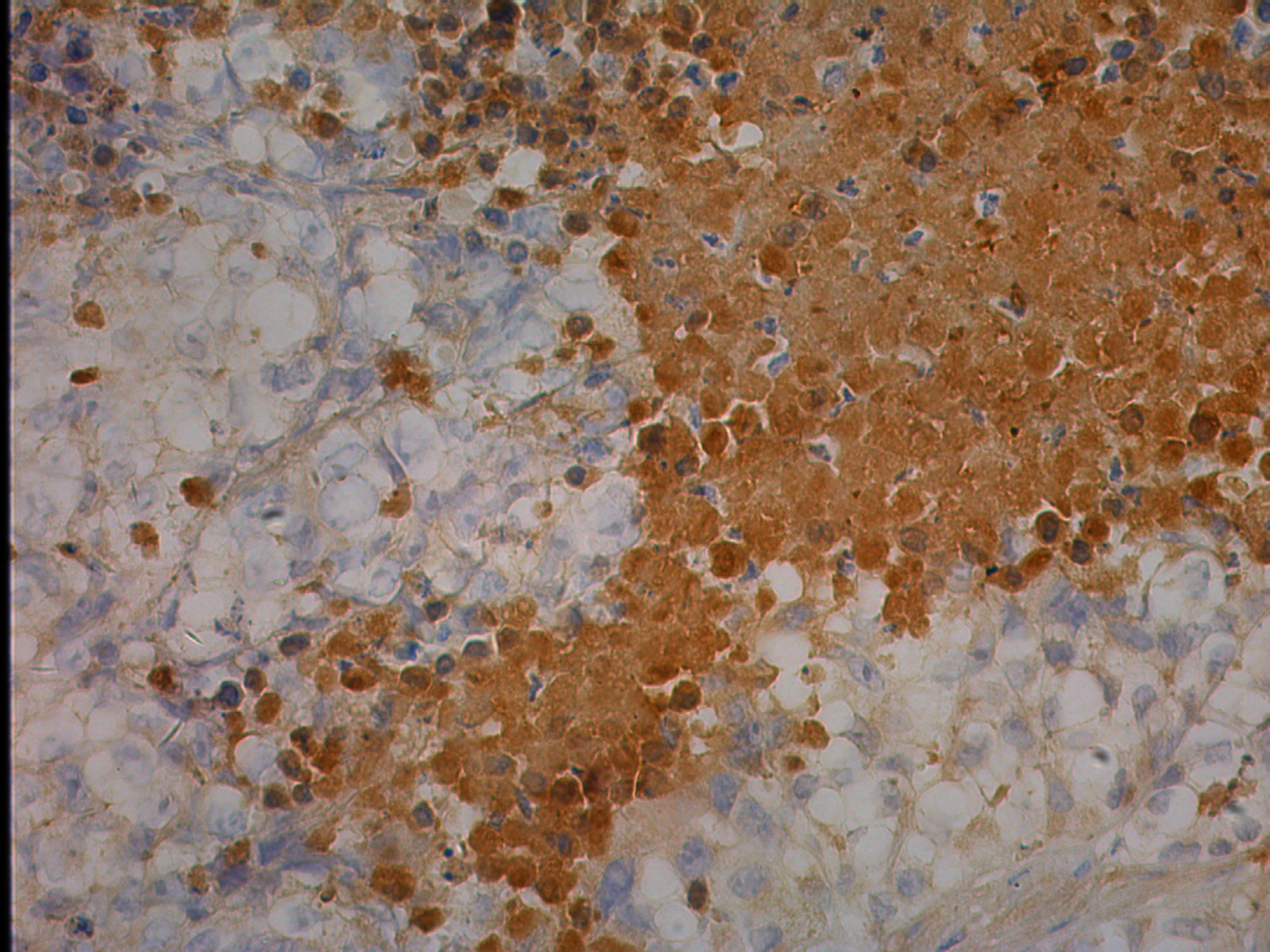

Supplement: S2 File — (ZIP) [file pone.0153540.s002.zip › S2 File/Fig.5/Fig.5A/Fig.5A lower panel IHC/M7.tif]

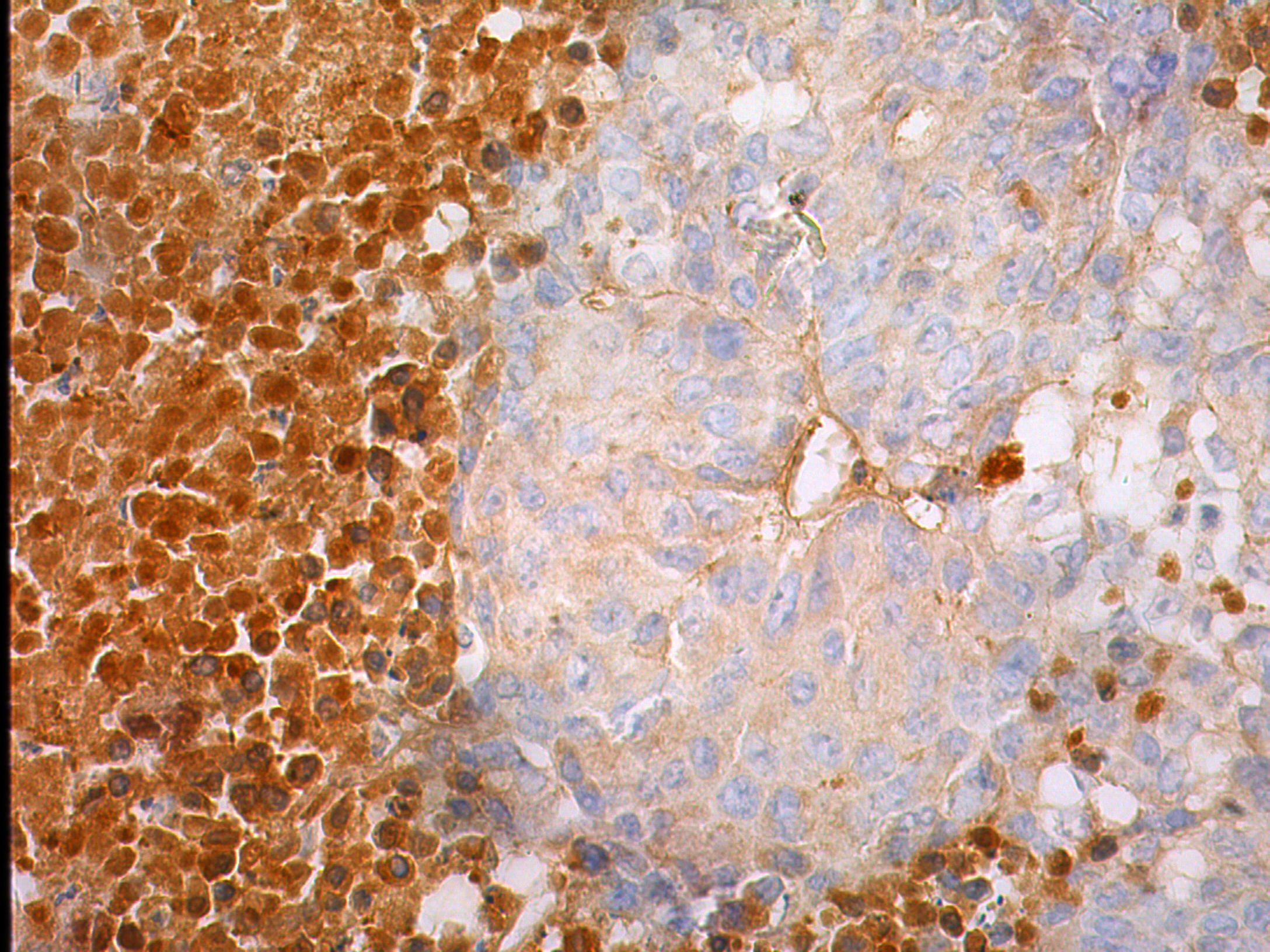

Supplement: S2 File — (ZIP) [file pone.0153540.s002.zip › S2 File/Fig.5/Fig.5A/Fig.5A lower panel IHC/M8.tif]

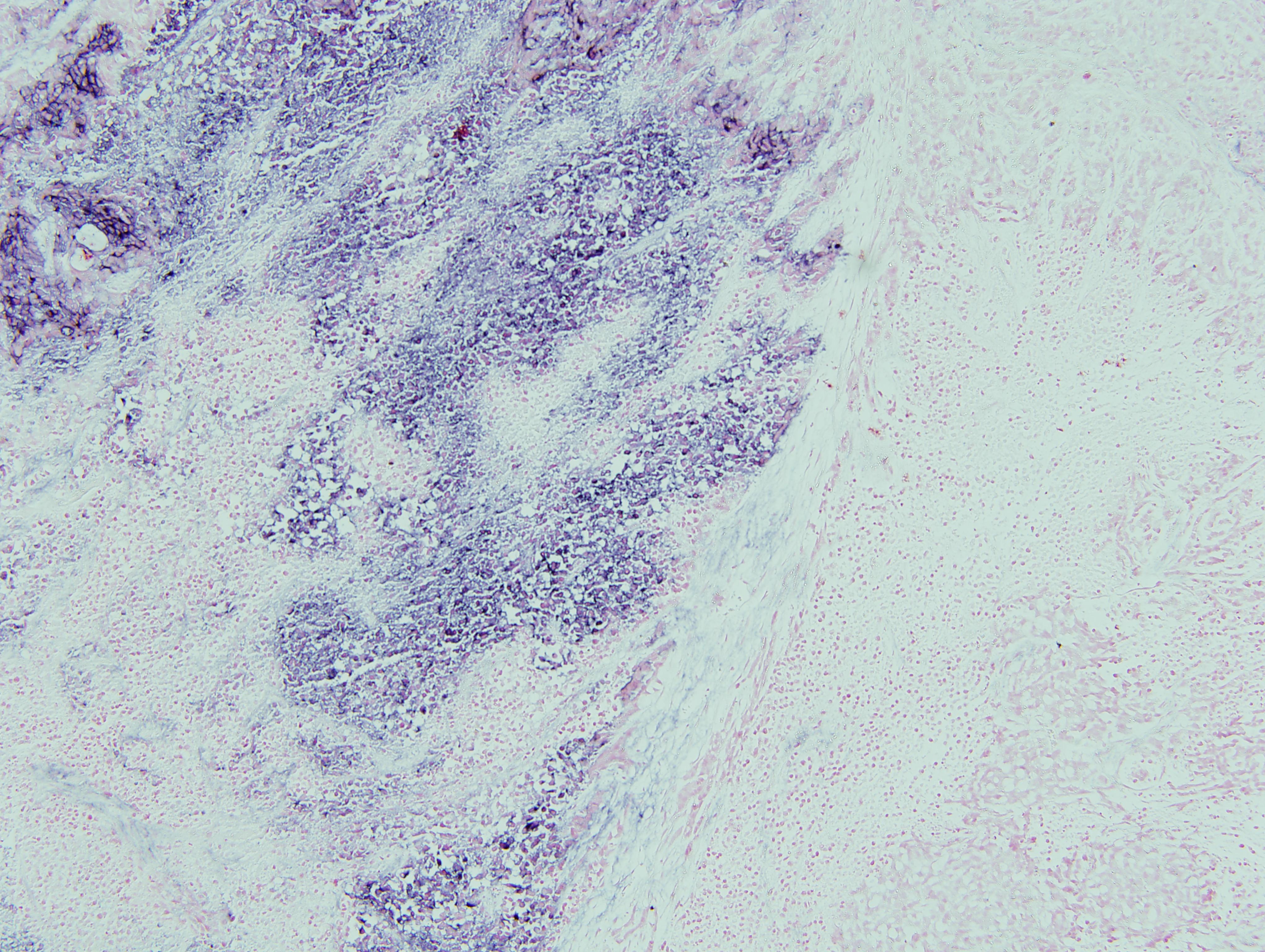

Supplement: S2 File — (ZIP) [file pone.0153540.s002.zip › S2 File/Fig.5/Fig.5A/Fig.5A middle panel in situ/Ad5dE1AdADP.jpg]

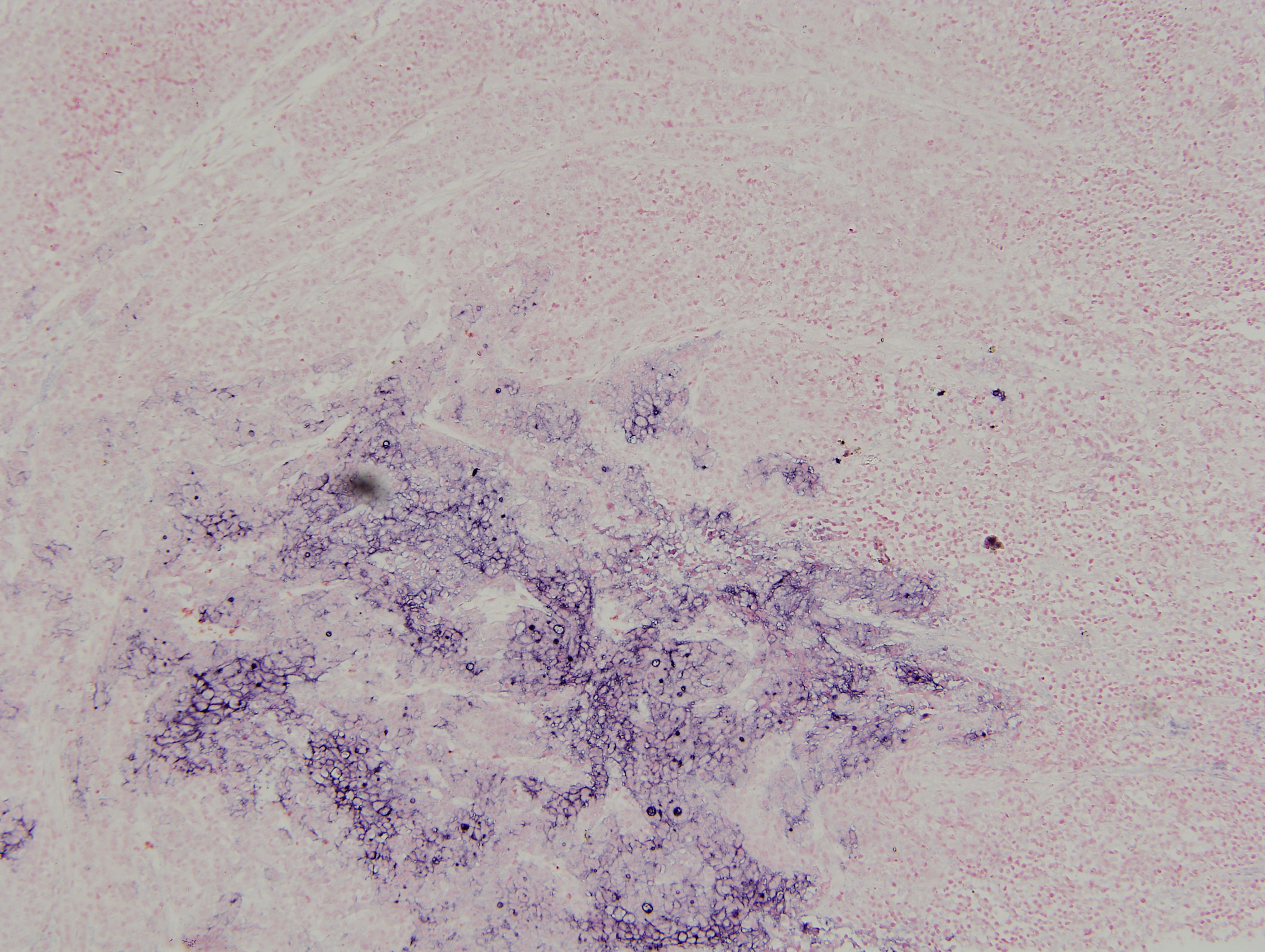

Supplement: S2 File — (ZIP) [file pone.0153540.s002.zip › S2 File/Fig.5/Fig.5A/Fig.5A middle panel in situ/Ad5dE1Adgp19k.jpg]

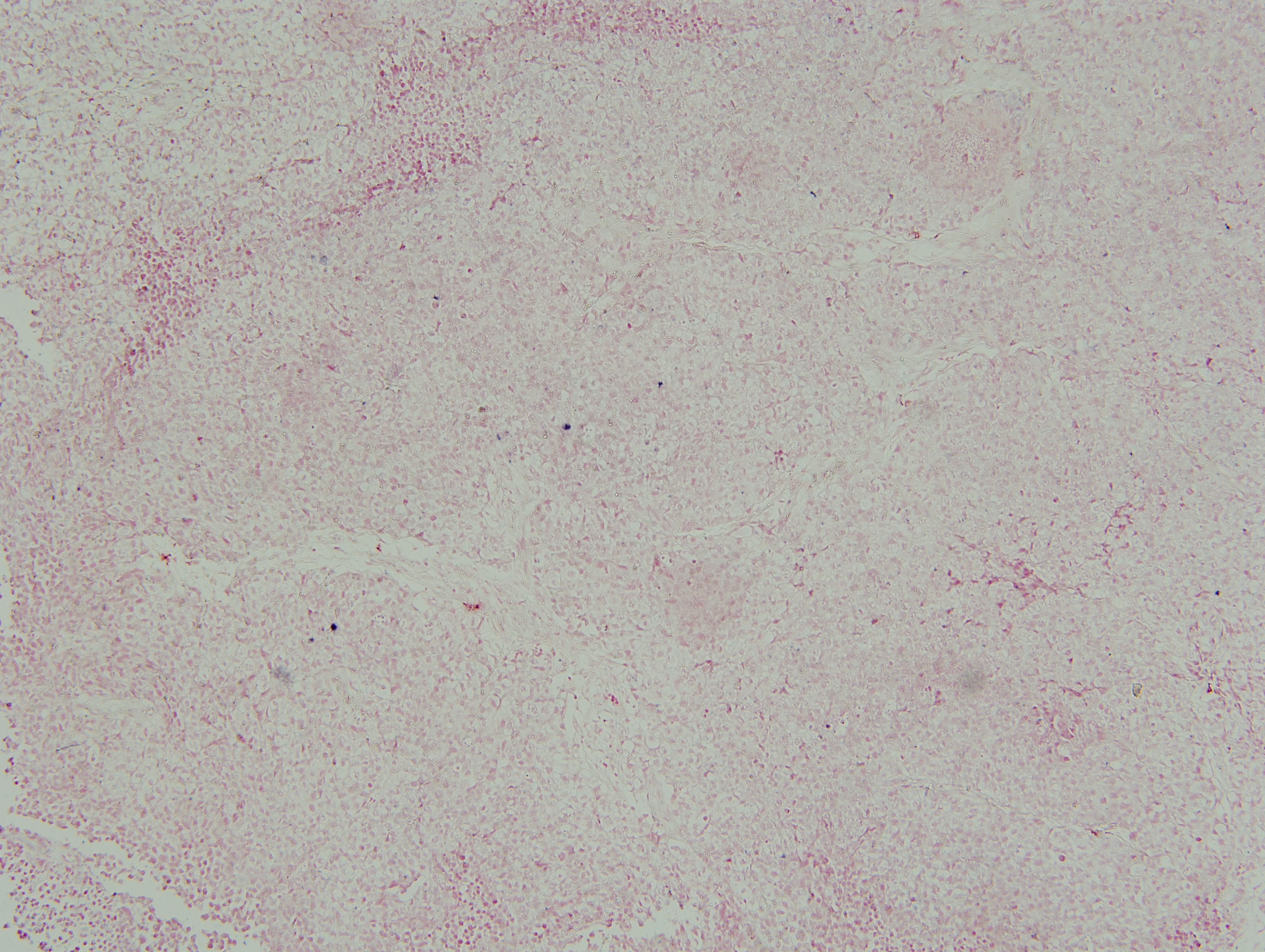

Supplement: S2 File — (ZIP) [file pone.0153540.s002.zip › S2 File/Fig.5/Fig.5A/Fig.5A middle panel in situ/Adv-TK.jpg]

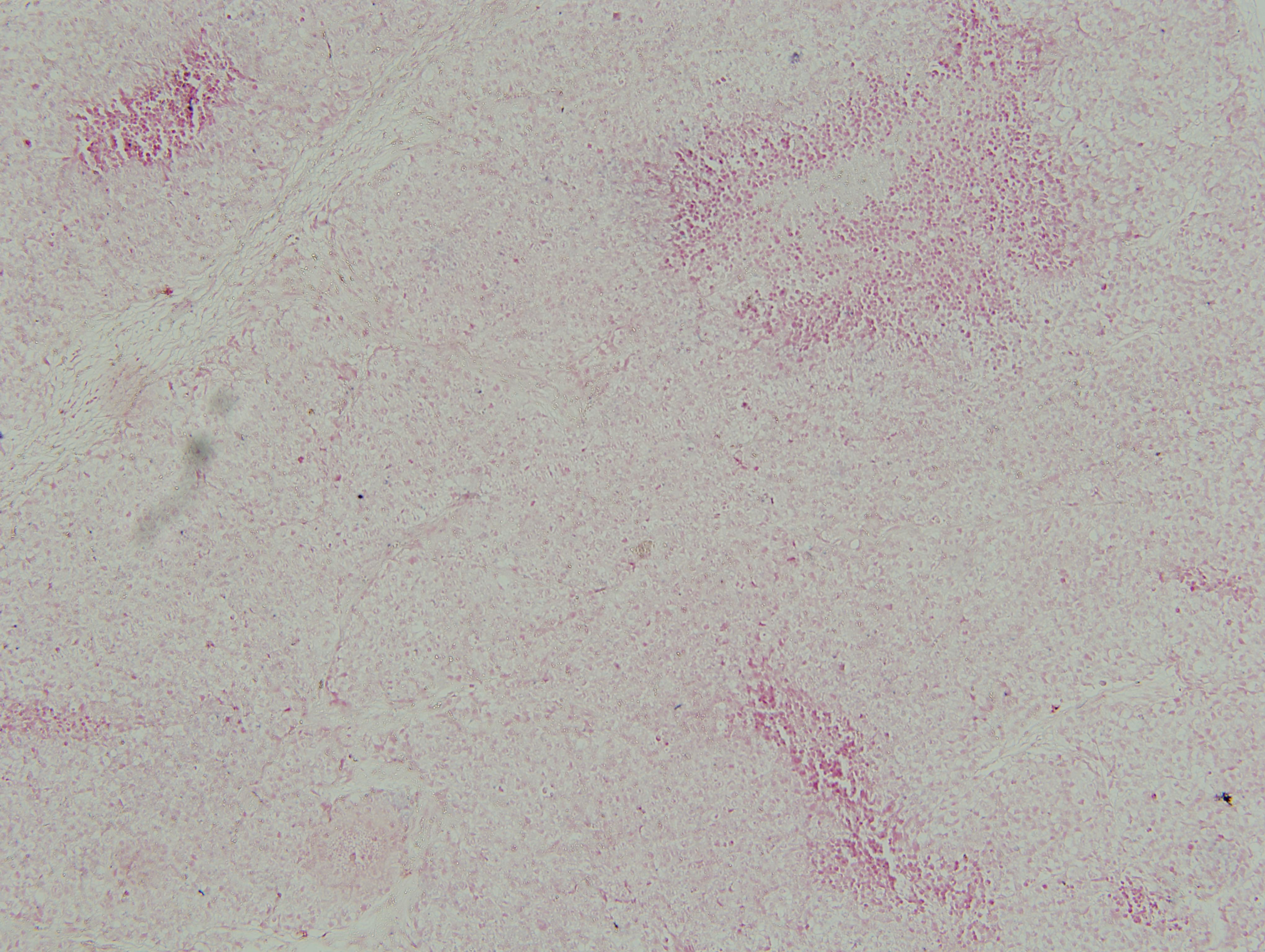

Supplement: S2 File — (ZIP) [file pone.0153540.s002.zip › S2 File/Fig.5/Fig.5A/Fig.5A middle panel in situ/CONTROL.jpg]

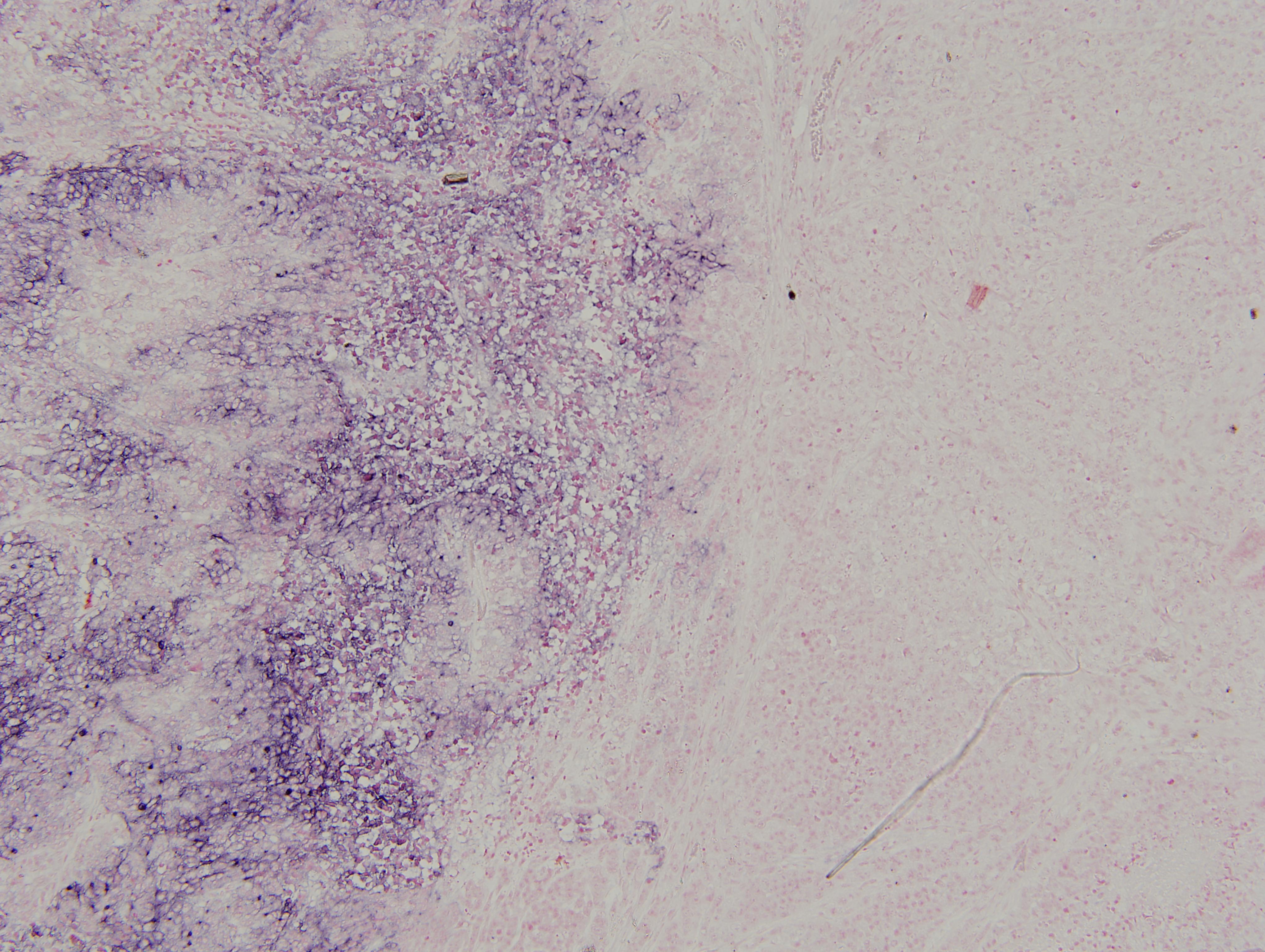

Supplement: S2 File — (ZIP) [file pone.0153540.s002.zip › S2 File/Fig.5/Fig.5A/Fig.5A middle panel in situ/M7.jpg]

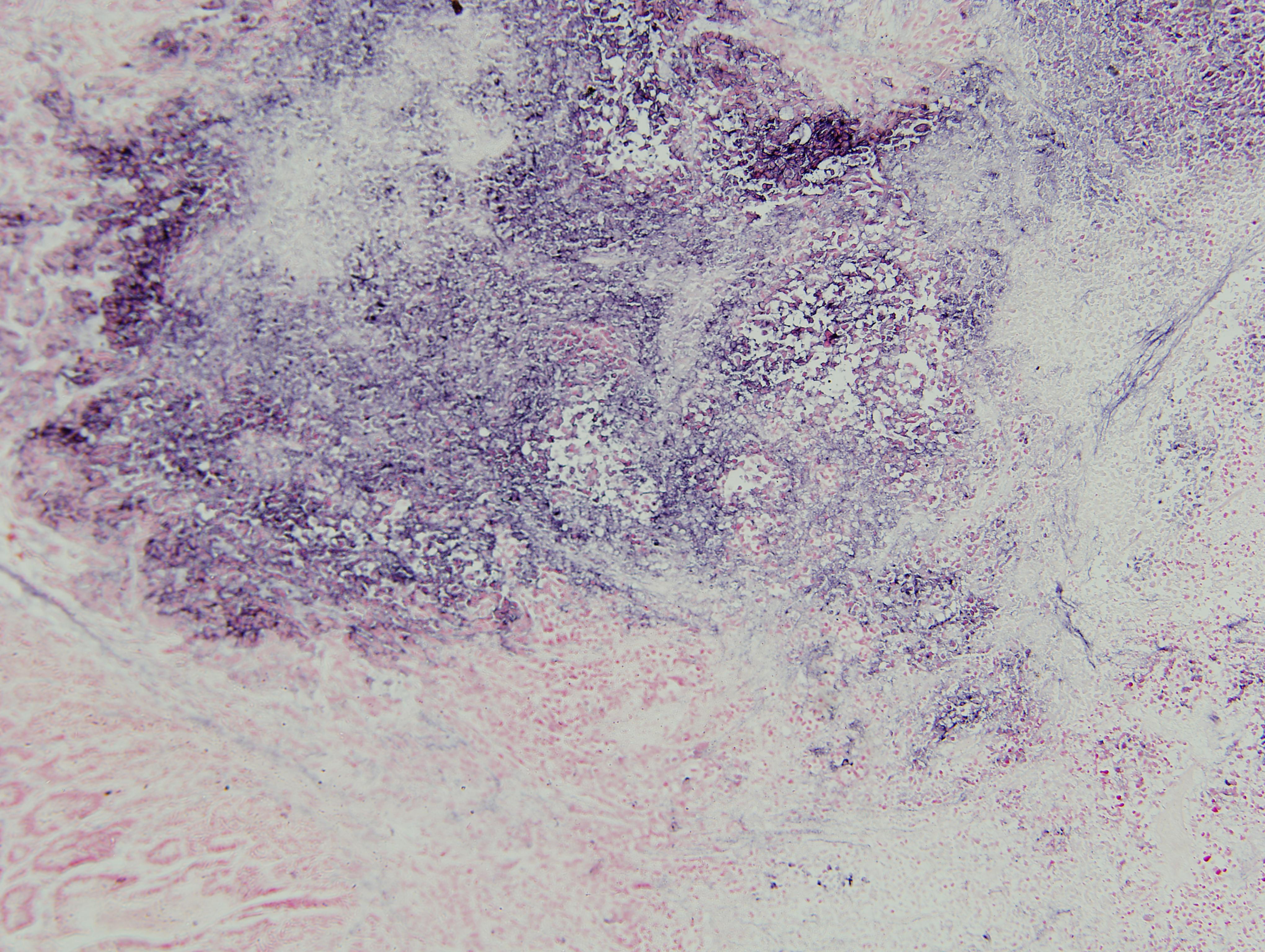

Supplement: S2 File — (ZIP) [file pone.0153540.s002.zip › S2 File/Fig.5/Fig.5A/Fig.5A middle panel in situ/M8.jpg]

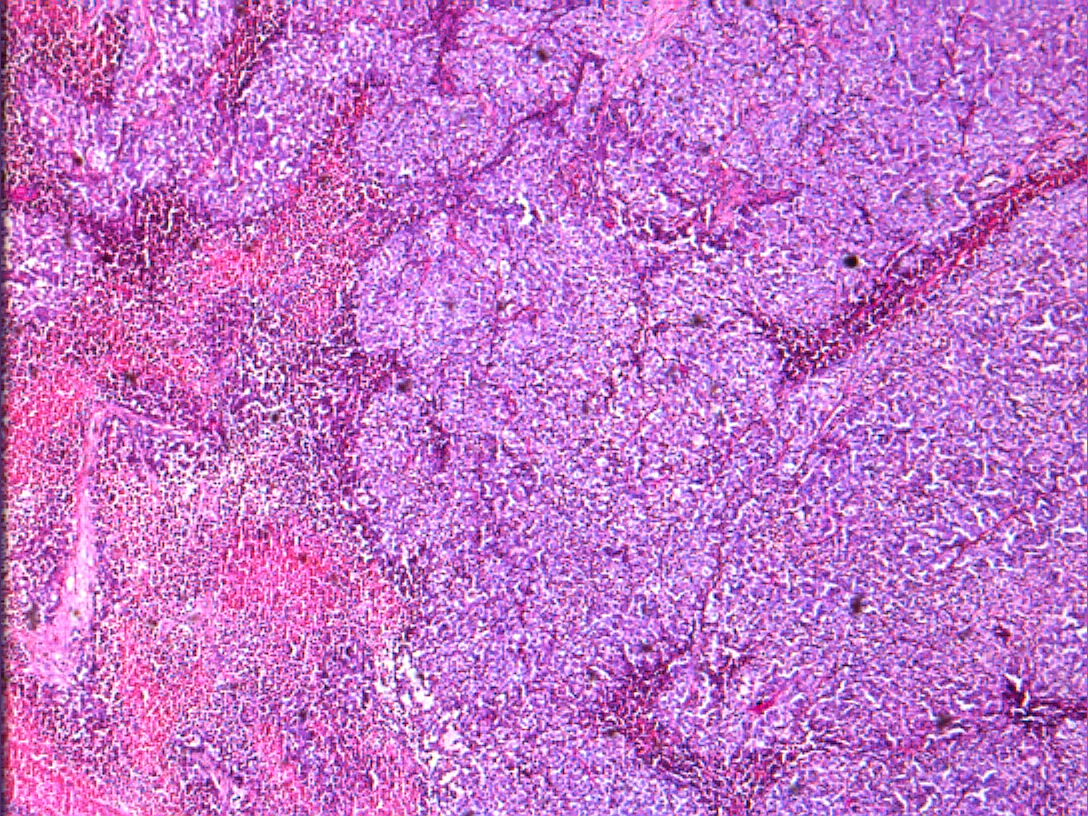

Supplement: S2 File — (ZIP) [file pone.0153540.s002.zip › S2 File/Fig.5/Fig.5A/Fig.5A upper panel HE/Ad5dE1AdADP.tif]

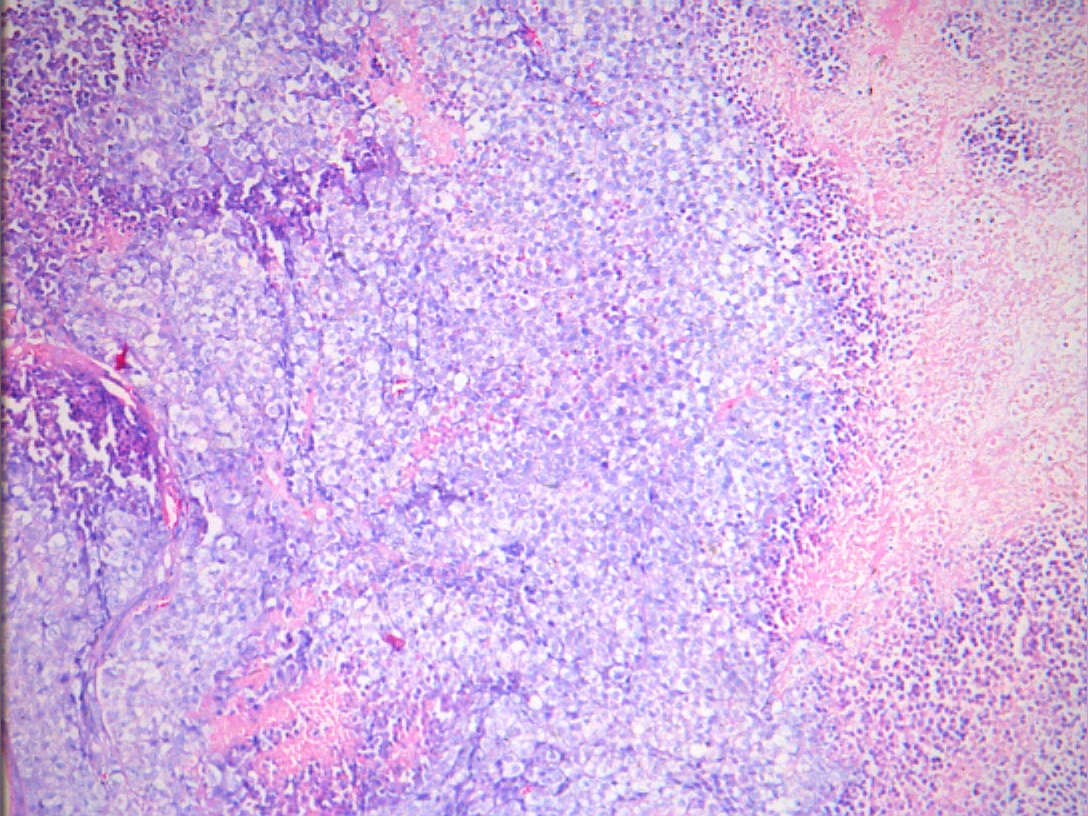

Supplement: S2 File — (ZIP) [file pone.0153540.s002.zip › S2 File/Fig.5/Fig.5A/Fig.5A upper panel HE/Ad5dE1Adgp19k.tif]

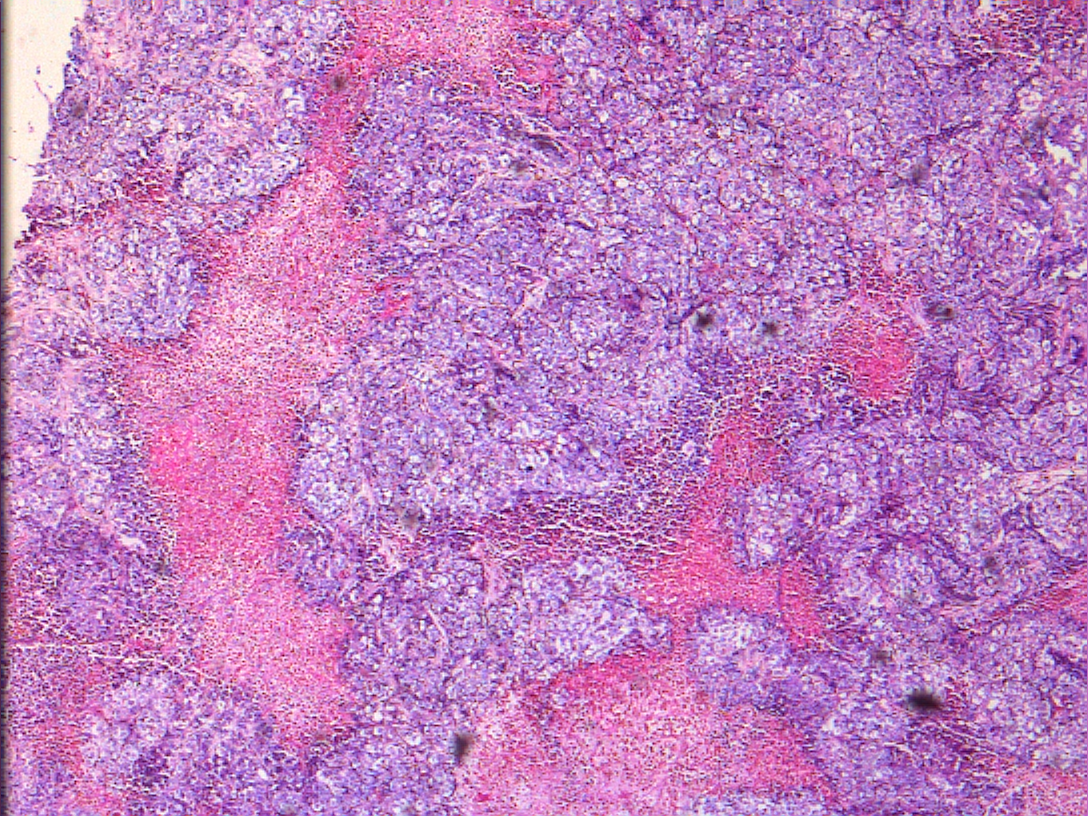

Supplement: S2 File — (ZIP) [file pone.0153540.s002.zip › S2 File/Fig.5/Fig.5A/Fig.5A upper panel HE/Adv-TK.tif]

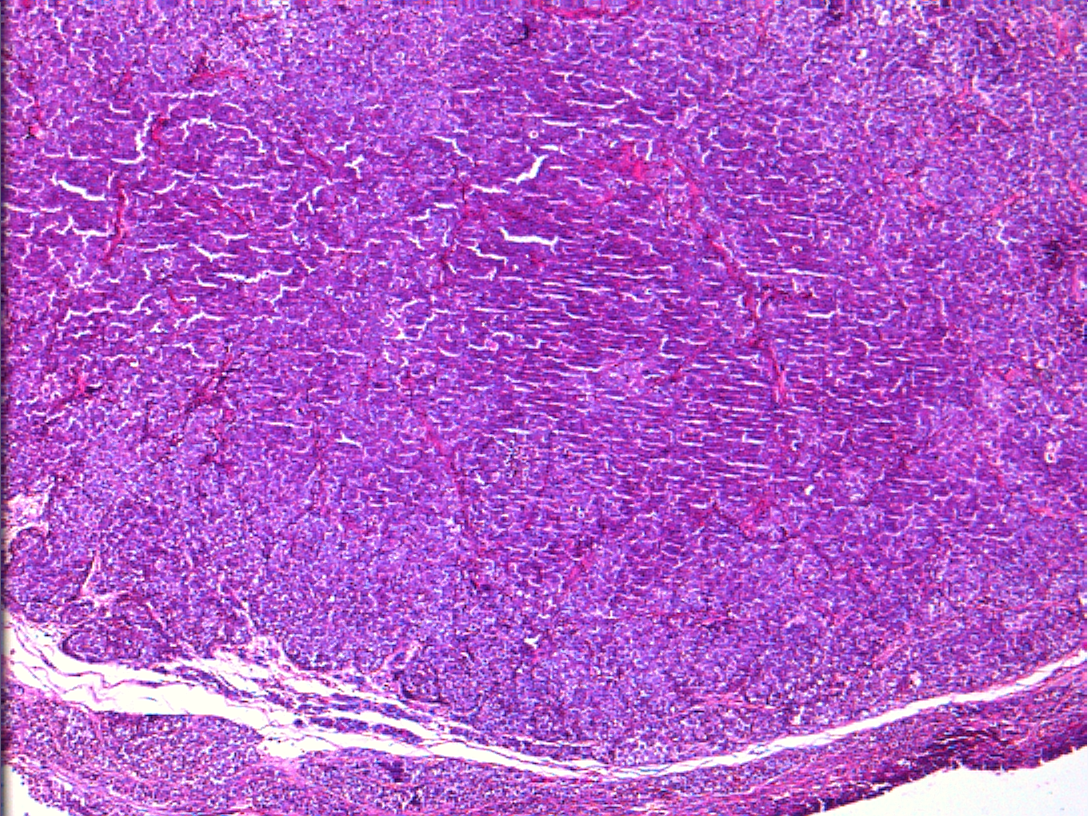

Supplement: S2 File — (ZIP) [file pone.0153540.s002.zip › S2 File/Fig.5/Fig.5A/Fig.5A upper panel HE/CONTROL.tif]

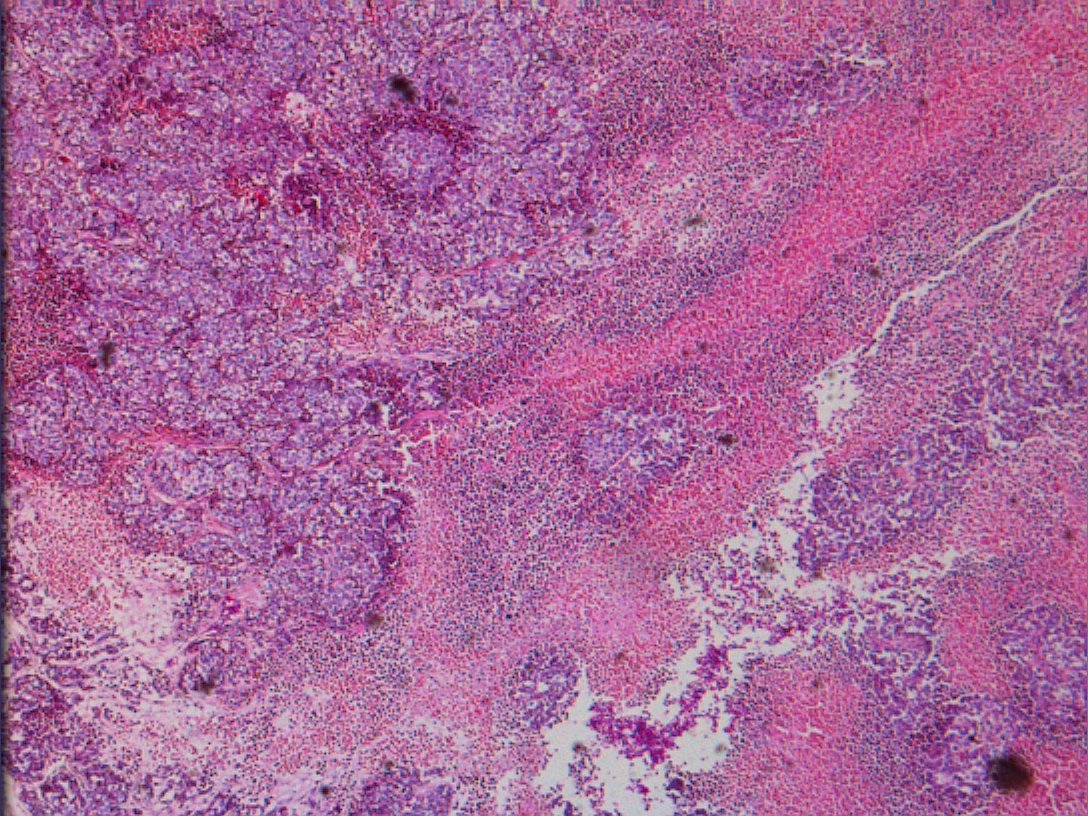

Supplement: S2 File — (ZIP) [file pone.0153540.s002.zip › S2 File/Fig.5/Fig.5A/Fig.5A upper panel HE/M7.tif]

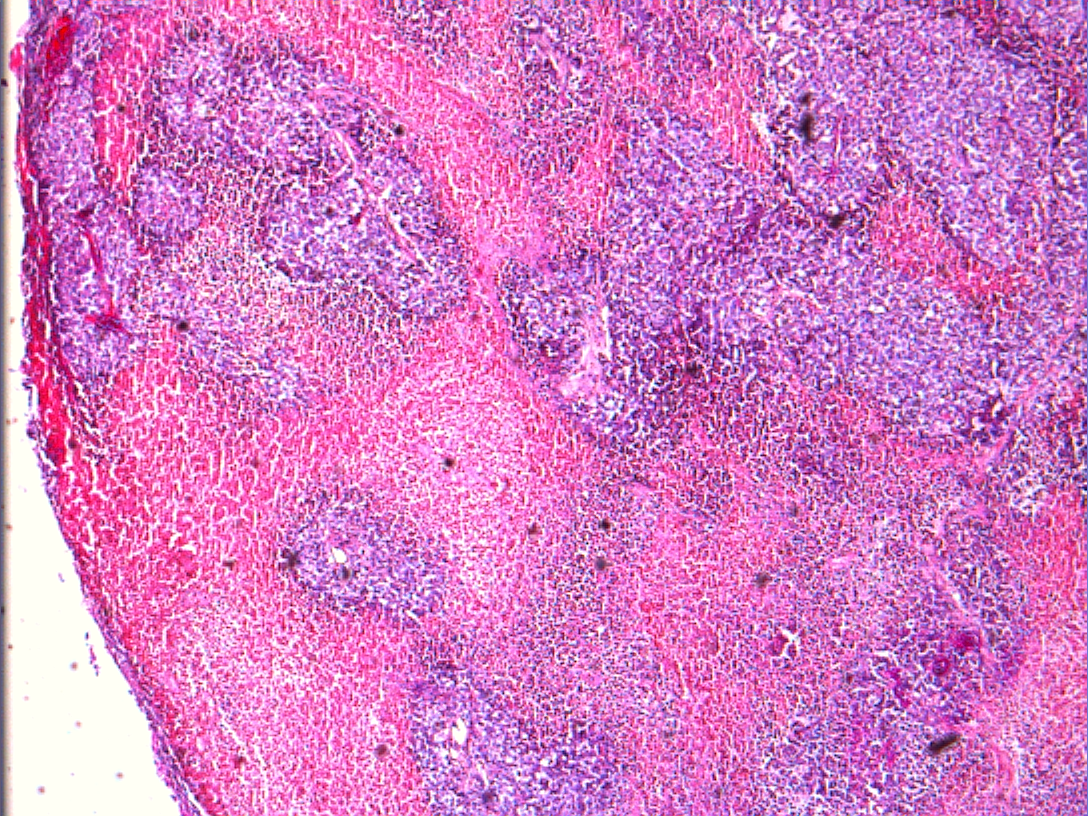

Supplement: S2 File — (ZIP) [file pone.0153540.s002.zip › S2 File/Fig.5/Fig.5A/Fig.5A upper panel HE/M8.tif]

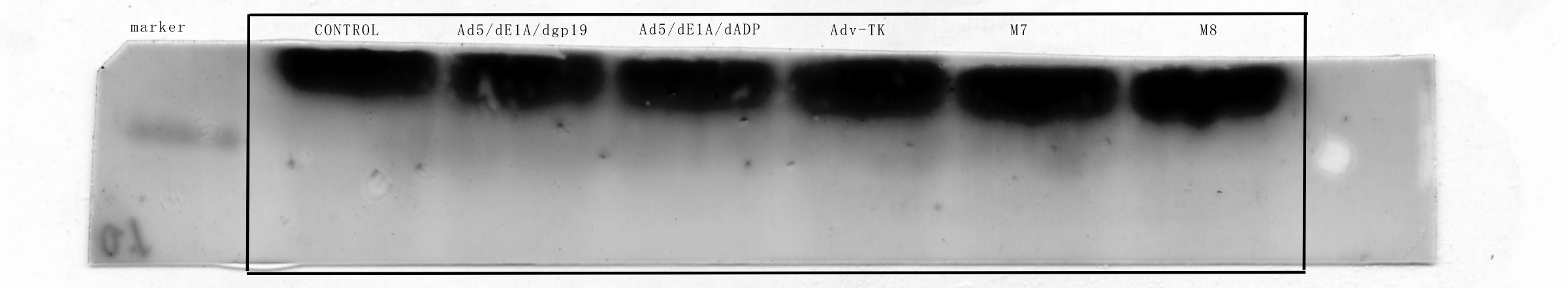

Supplement: S2 File — (ZIP) [file pone.0153540.s002.zip › S2 File/Fig.5/Fig.5D/actin.TIF]

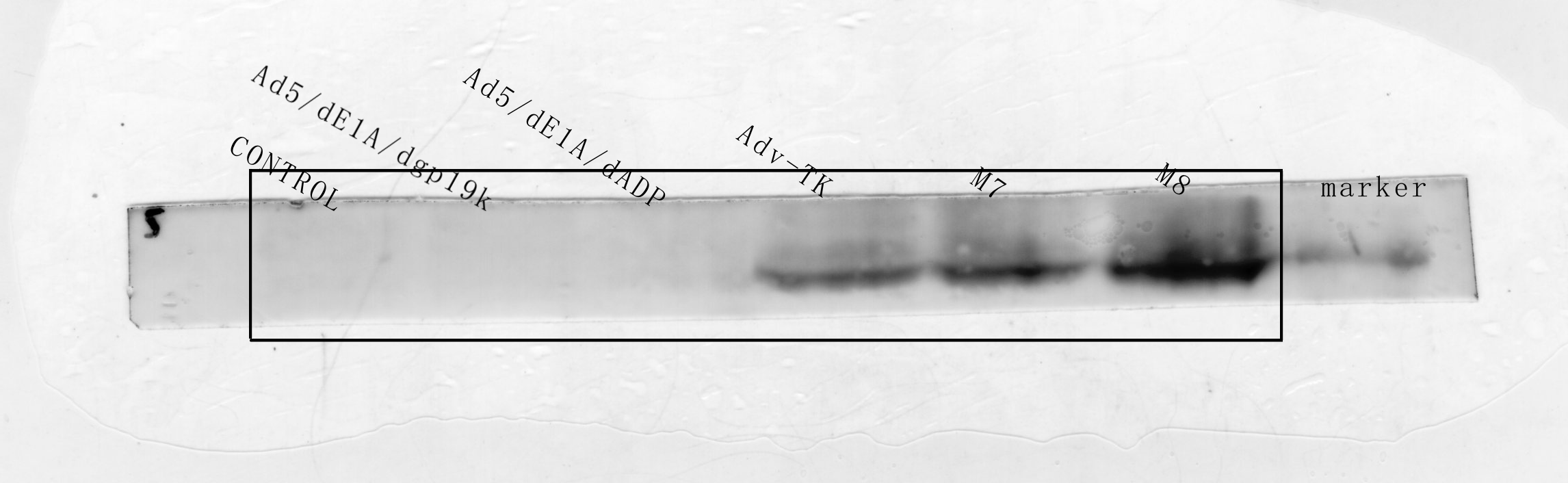

Supplement: S2 File — (ZIP) [file pone.0153540.s002.zip › S2 File/Fig.5/Fig.5D/TK.tif]

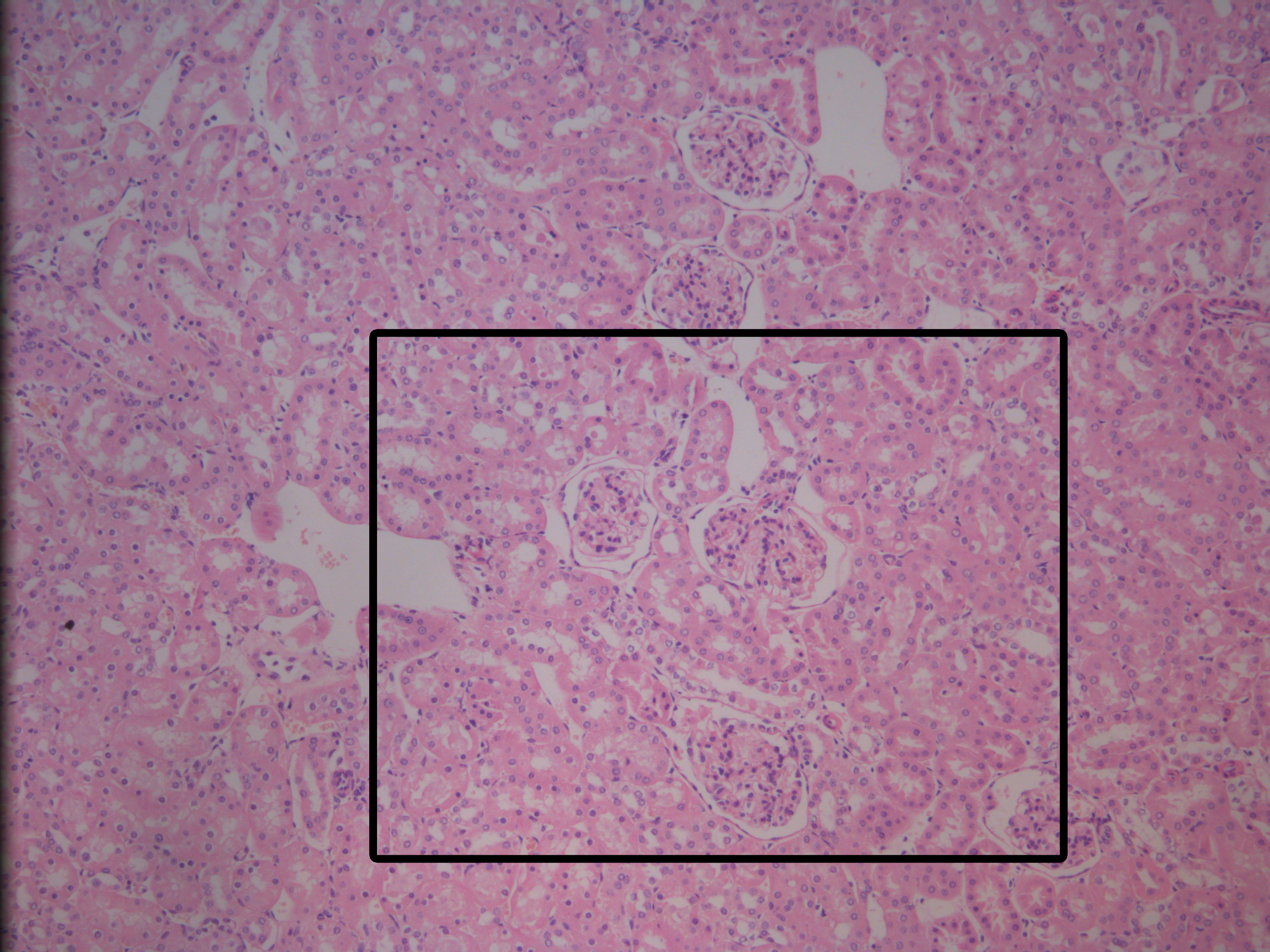

Supplement: S3 File — (ZIP) [file pone.0153540.s003.zip › S3 File/Fig.6E/KINDEY/Adv-TK+GCV.jpg]

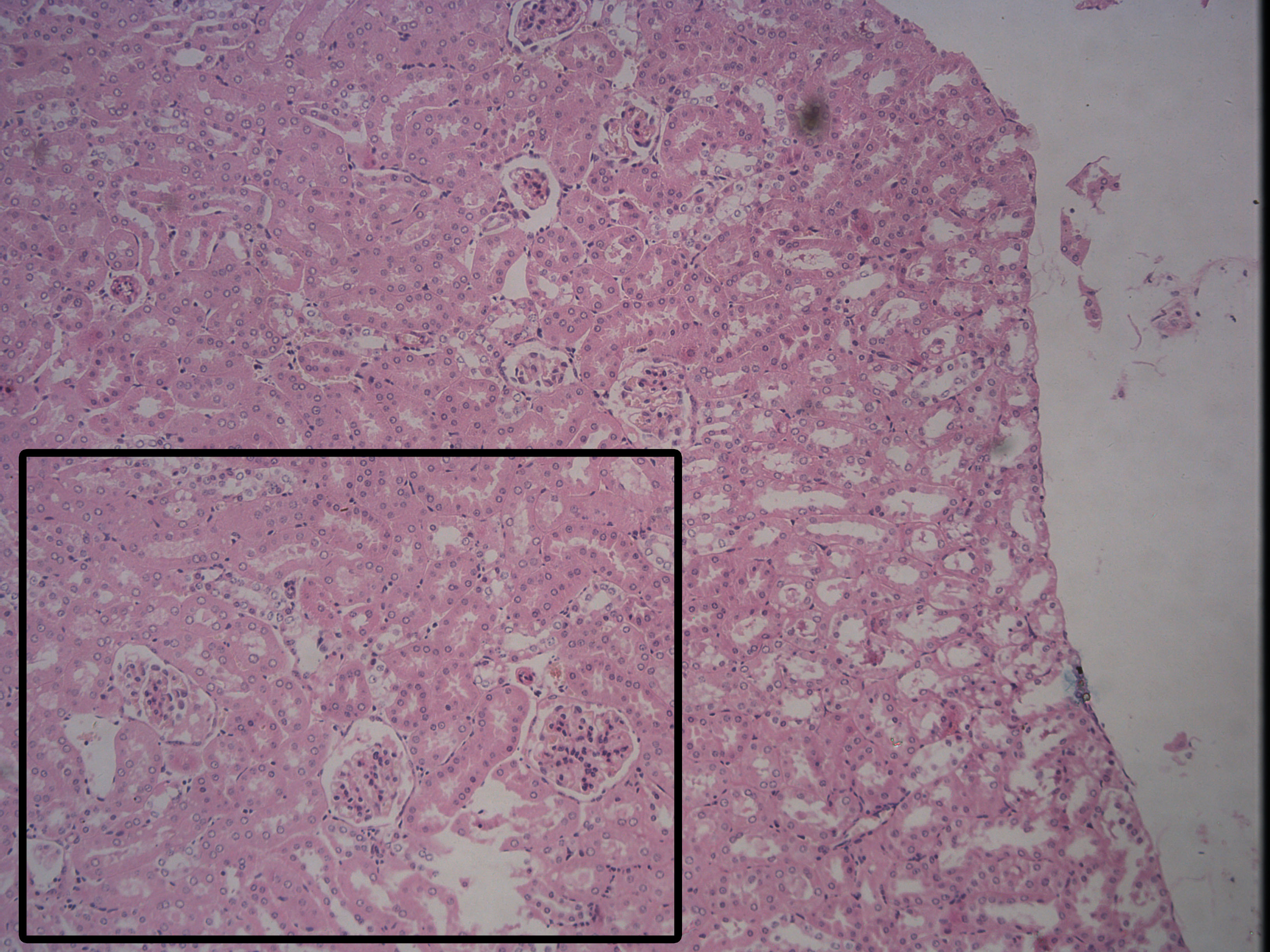

Supplement: S3 File — (ZIP) [file pone.0153540.s003.zip › S3 File/Fig.6E/KINDEY/CONTROL.jpg]

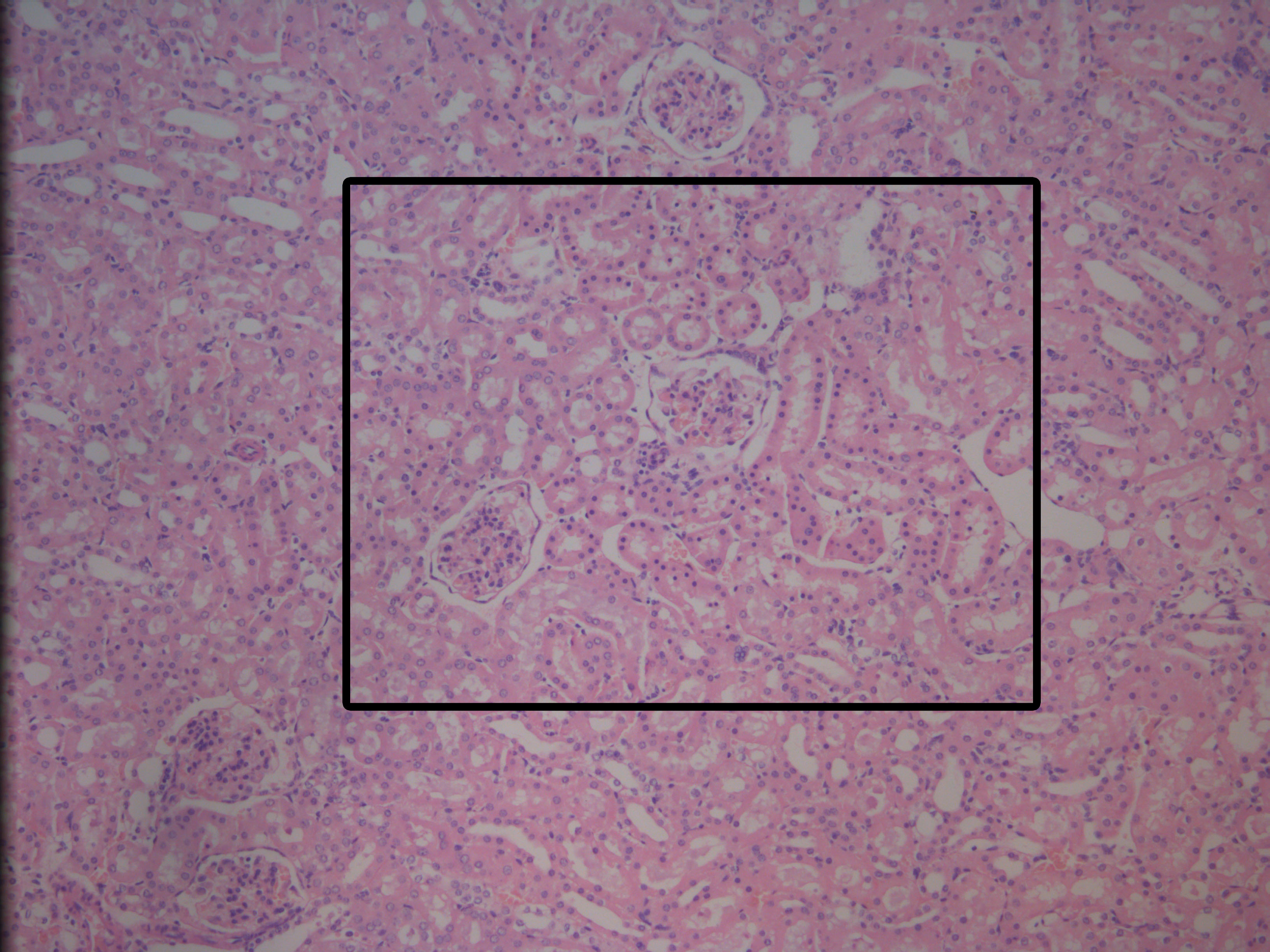

Supplement: S3 File — (ZIP) [file pone.0153540.s003.zip › S3 File/Fig.6E/KINDEY/GCV.jpg]

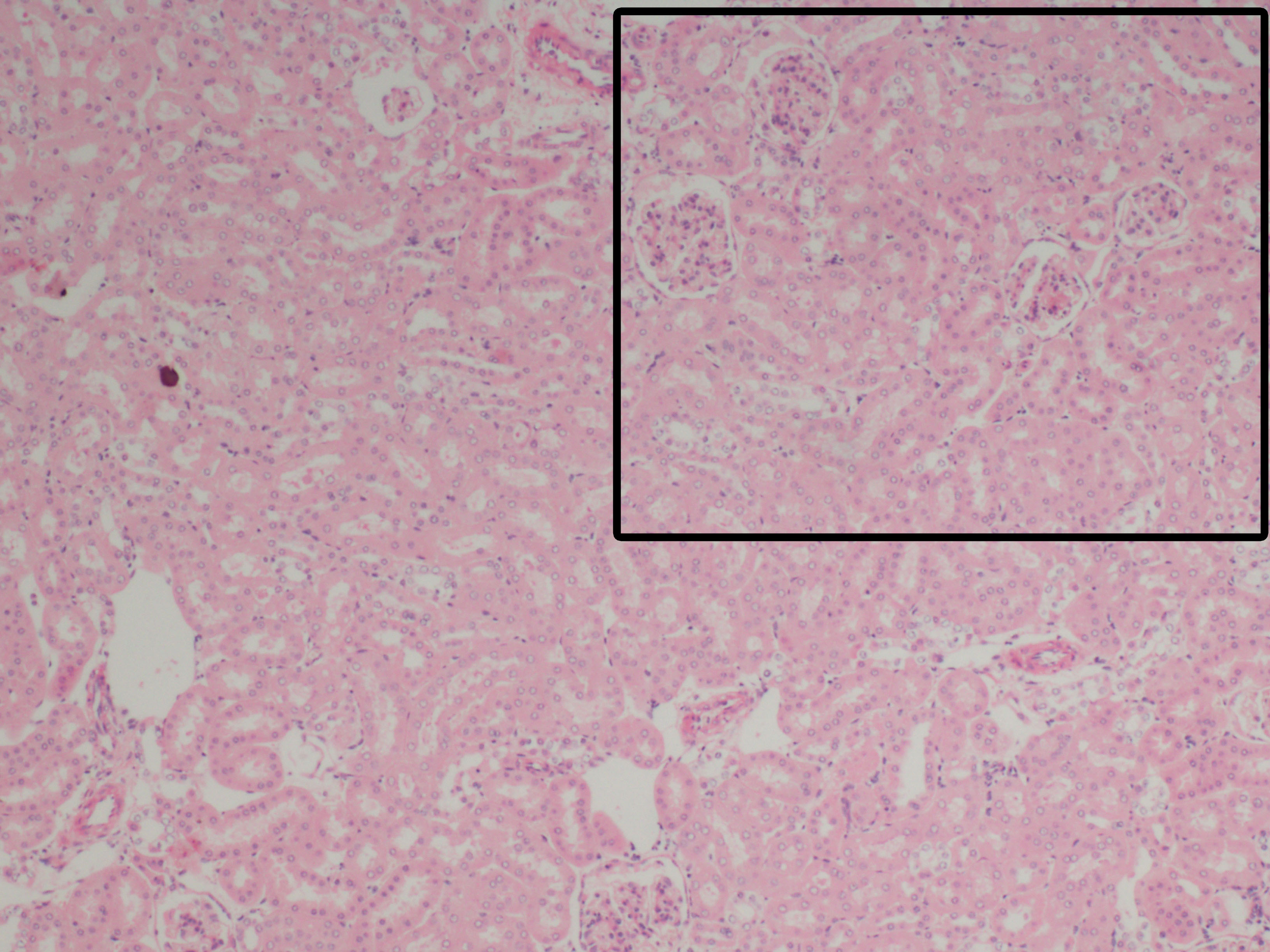

Supplement: S3 File — (ZIP) [file pone.0153540.s003.zip › S3 File/Fig.6E/KINDEY/M7+GCV.jpg]

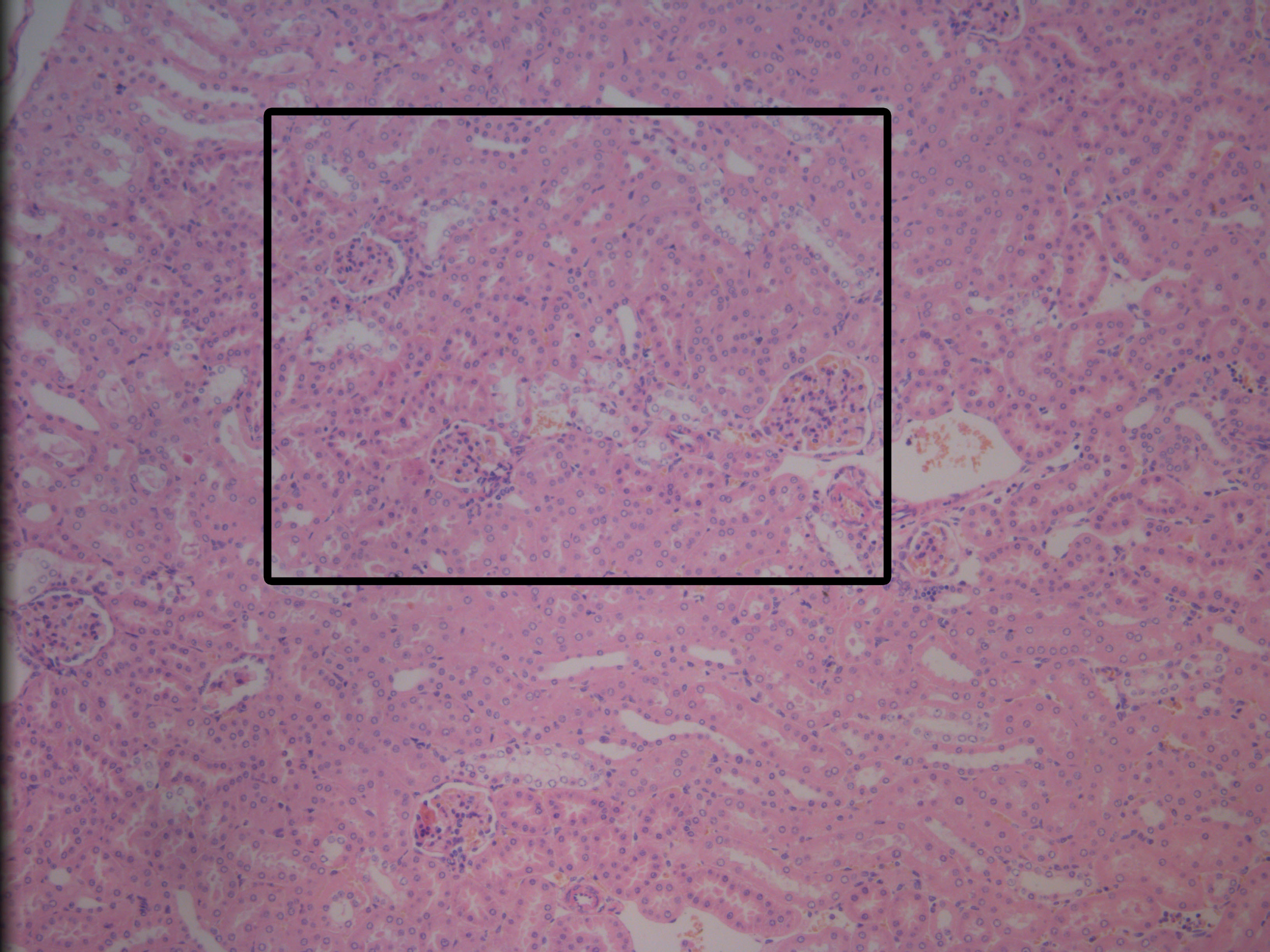

Supplement: S3 File — (ZIP) [file pone.0153540.s003.zip › S3 File/Fig.6E/KINDEY/M8+GCV.jpg]

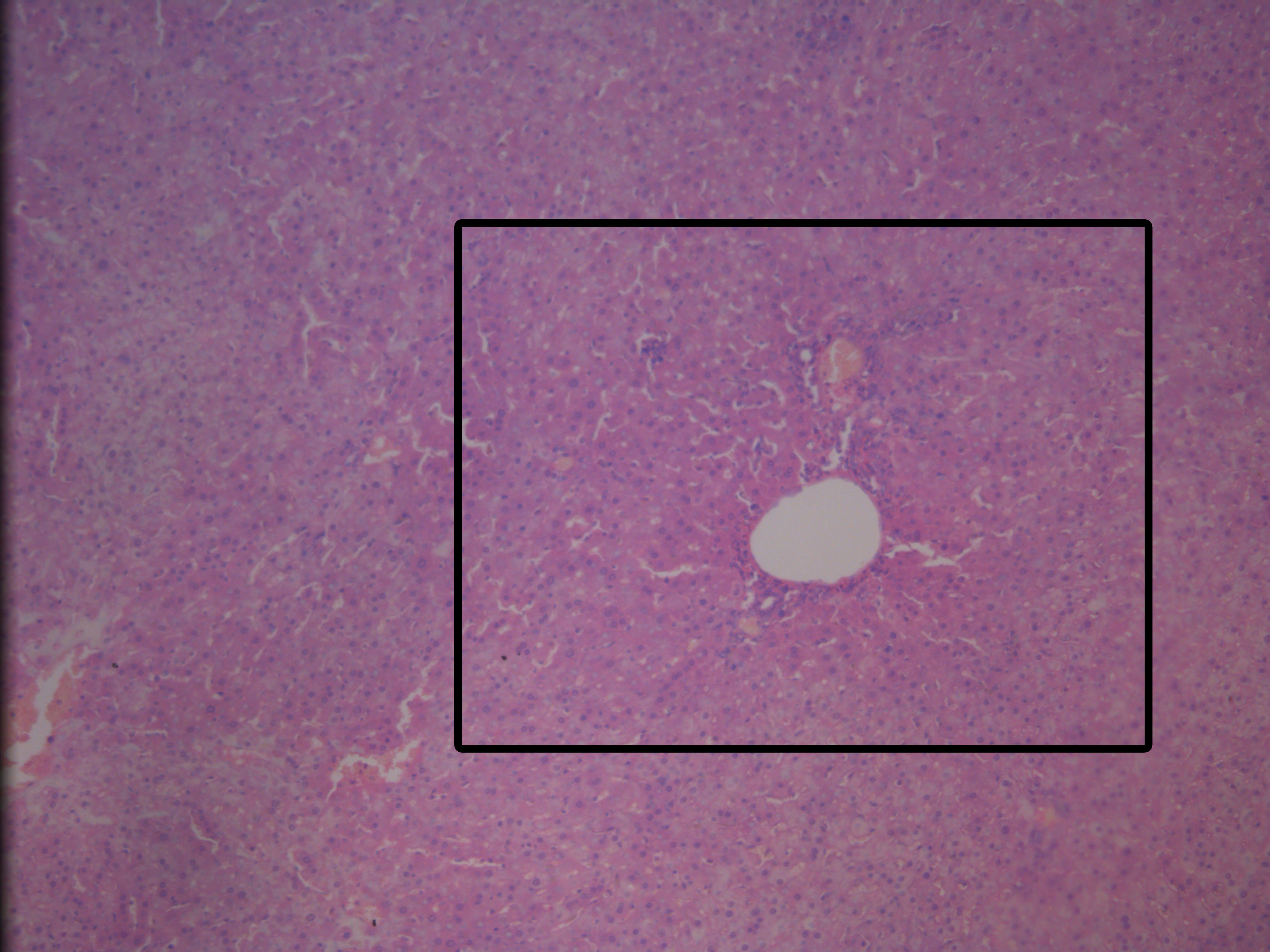

Supplement: S3 File — (ZIP) [file pone.0153540.s003.zip › S3 File/Fig.6E/LIVER/Adv-TK+GCV.jpg]

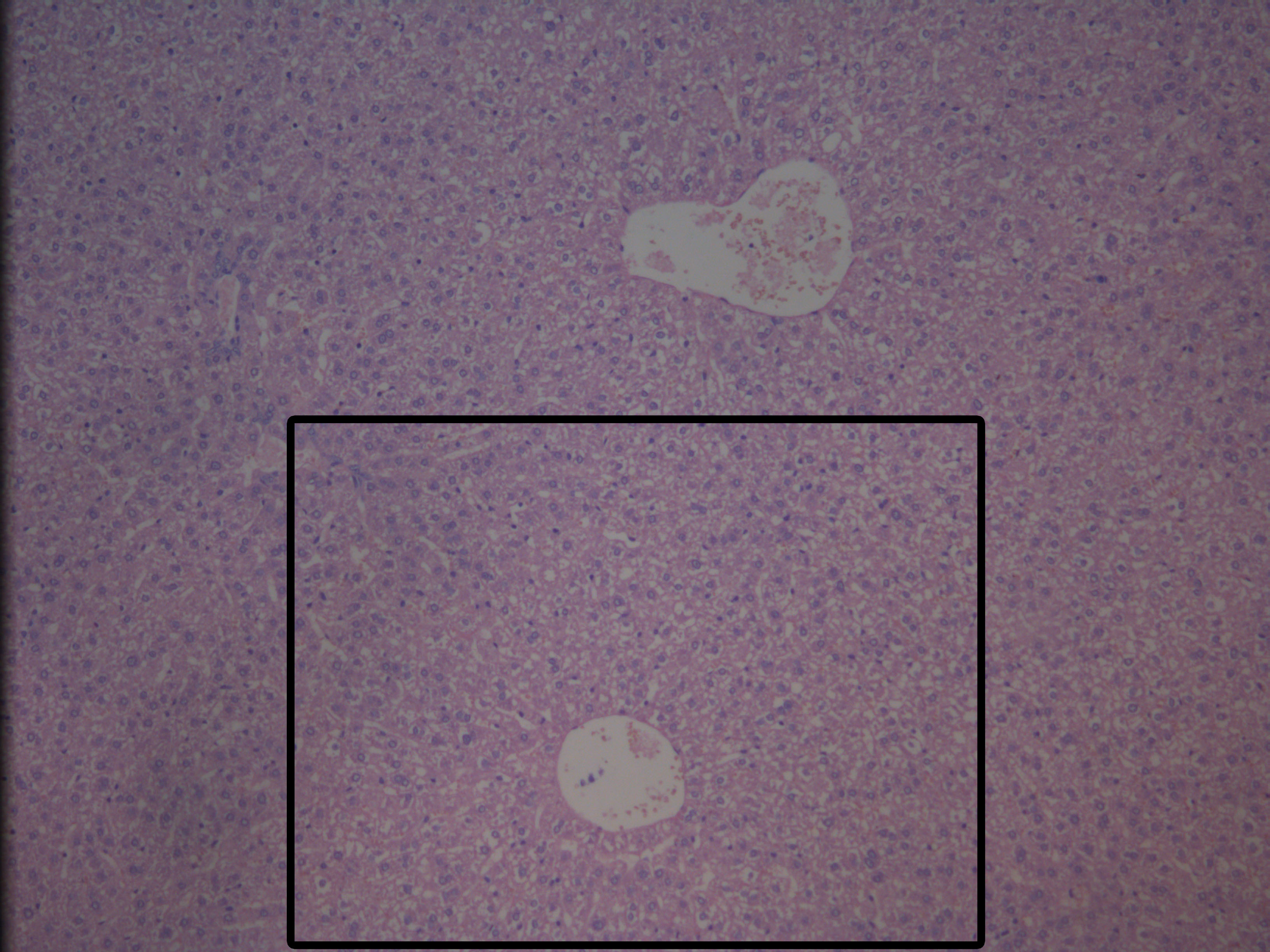

Supplement: S3 File — (ZIP) [file pone.0153540.s003.zip › S3 File/Fig.6E/LIVER/CONTROL.jpg]

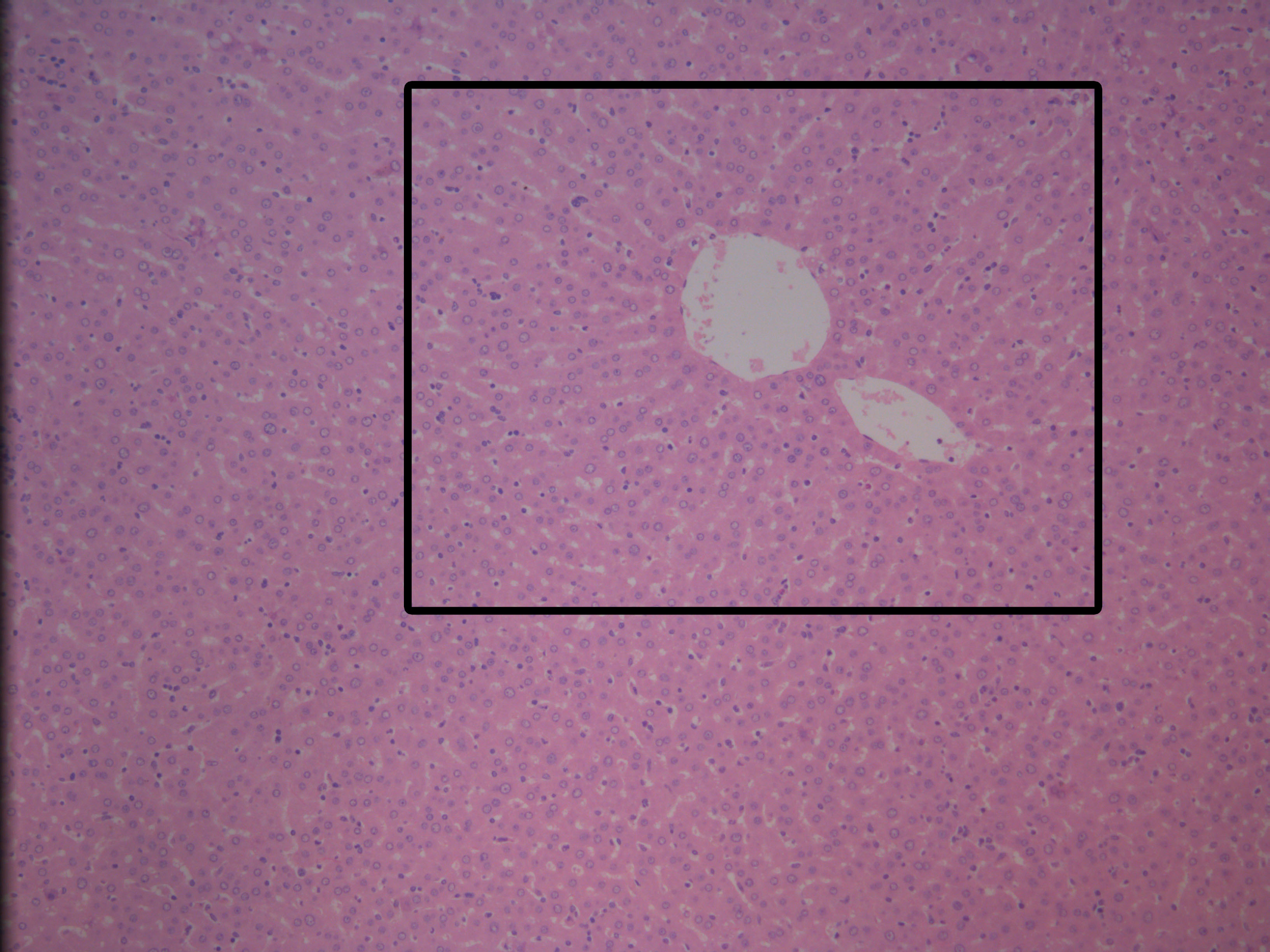

Supplement: S3 File — (ZIP) [file pone.0153540.s003.zip › S3 File/Fig.6E/LIVER/GCV.jpg]

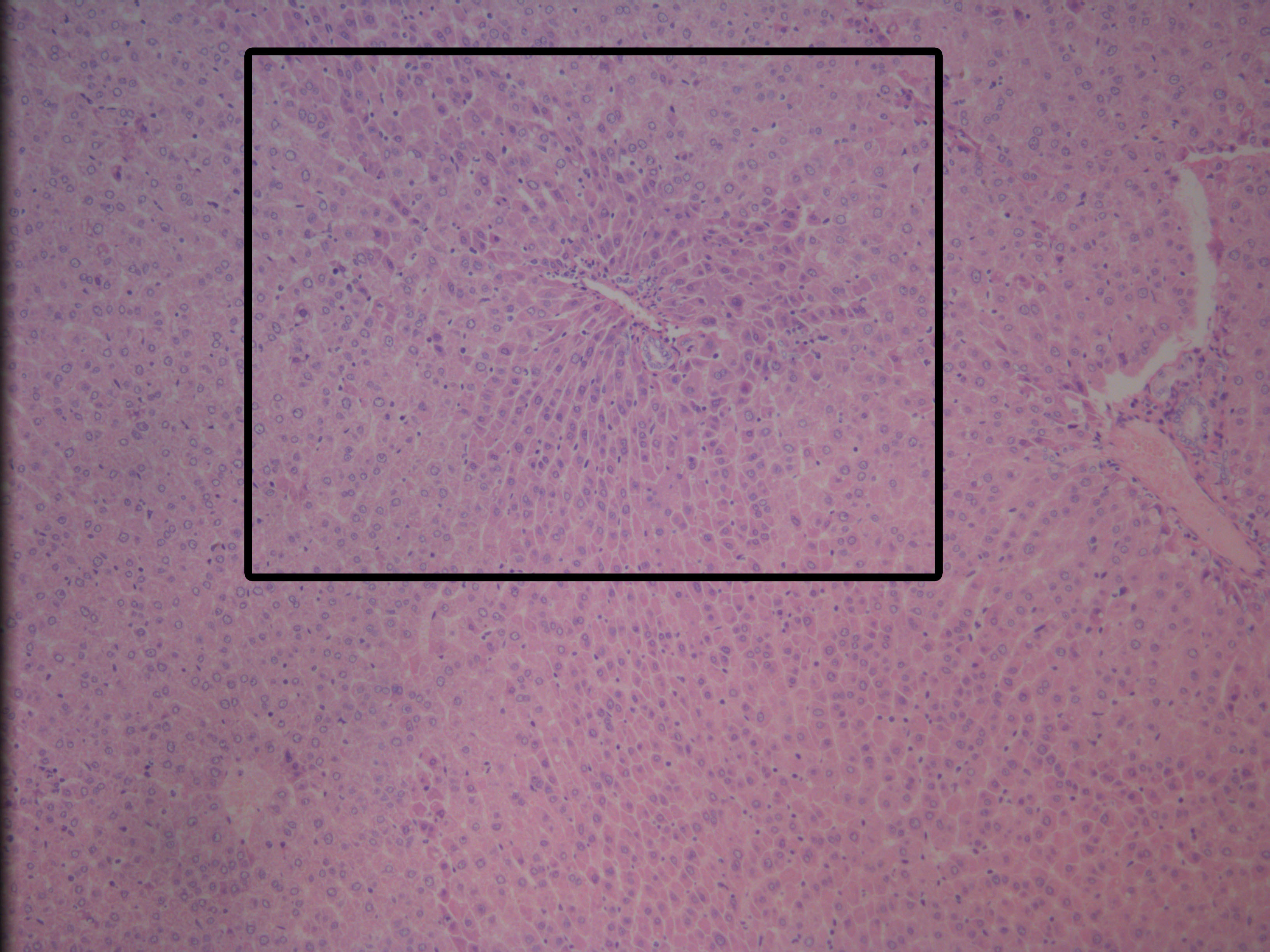

Supplement: S3 File — (ZIP) [file pone.0153540.s003.zip › S3 File/Fig.6E/LIVER/M7+GCV.jpg]

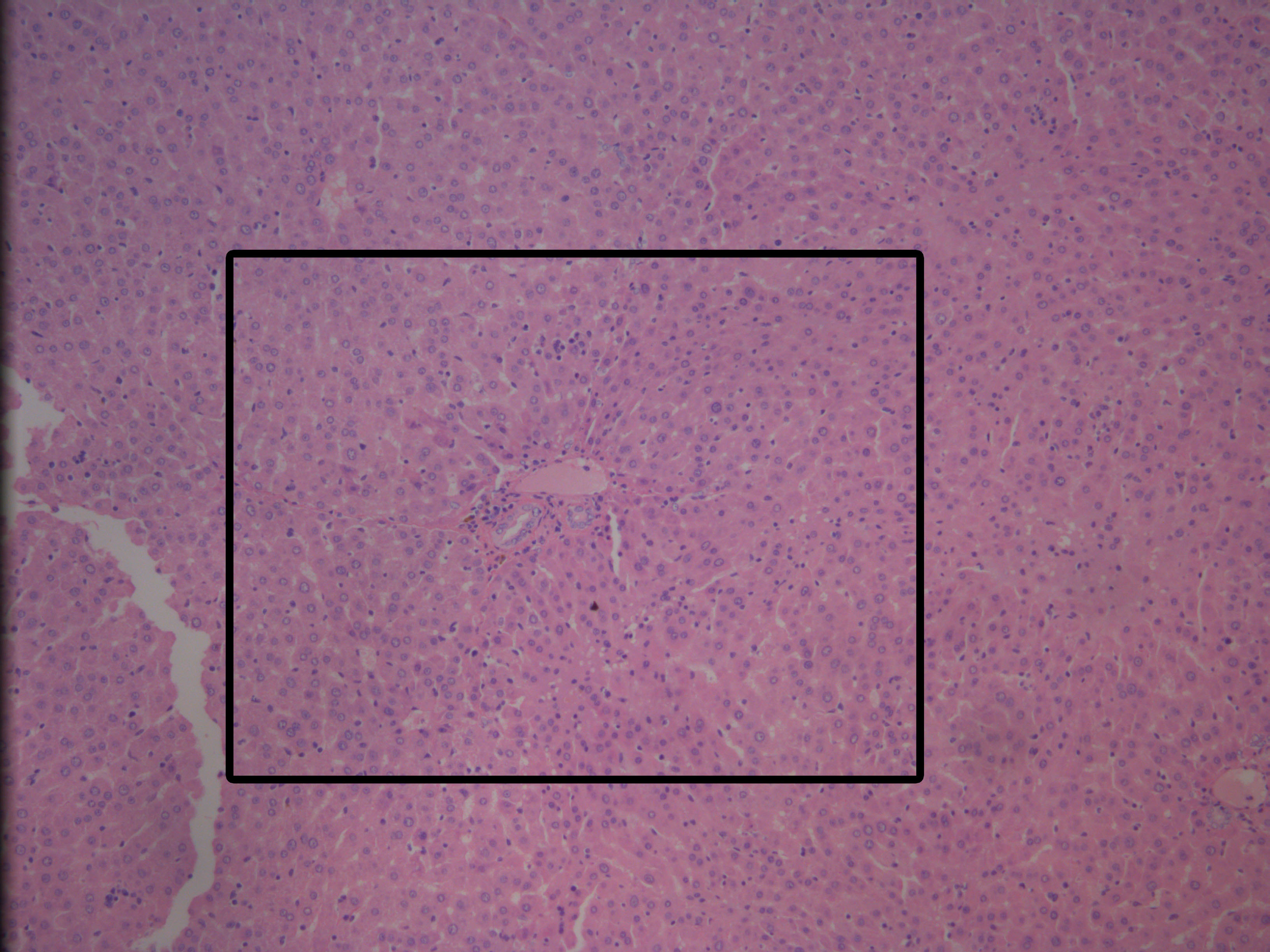

Supplement: S3 File — (ZIP) [file pone.0153540.s003.zip › S3 File/Fig.6E/LIVER/M8+GCV.jpg]

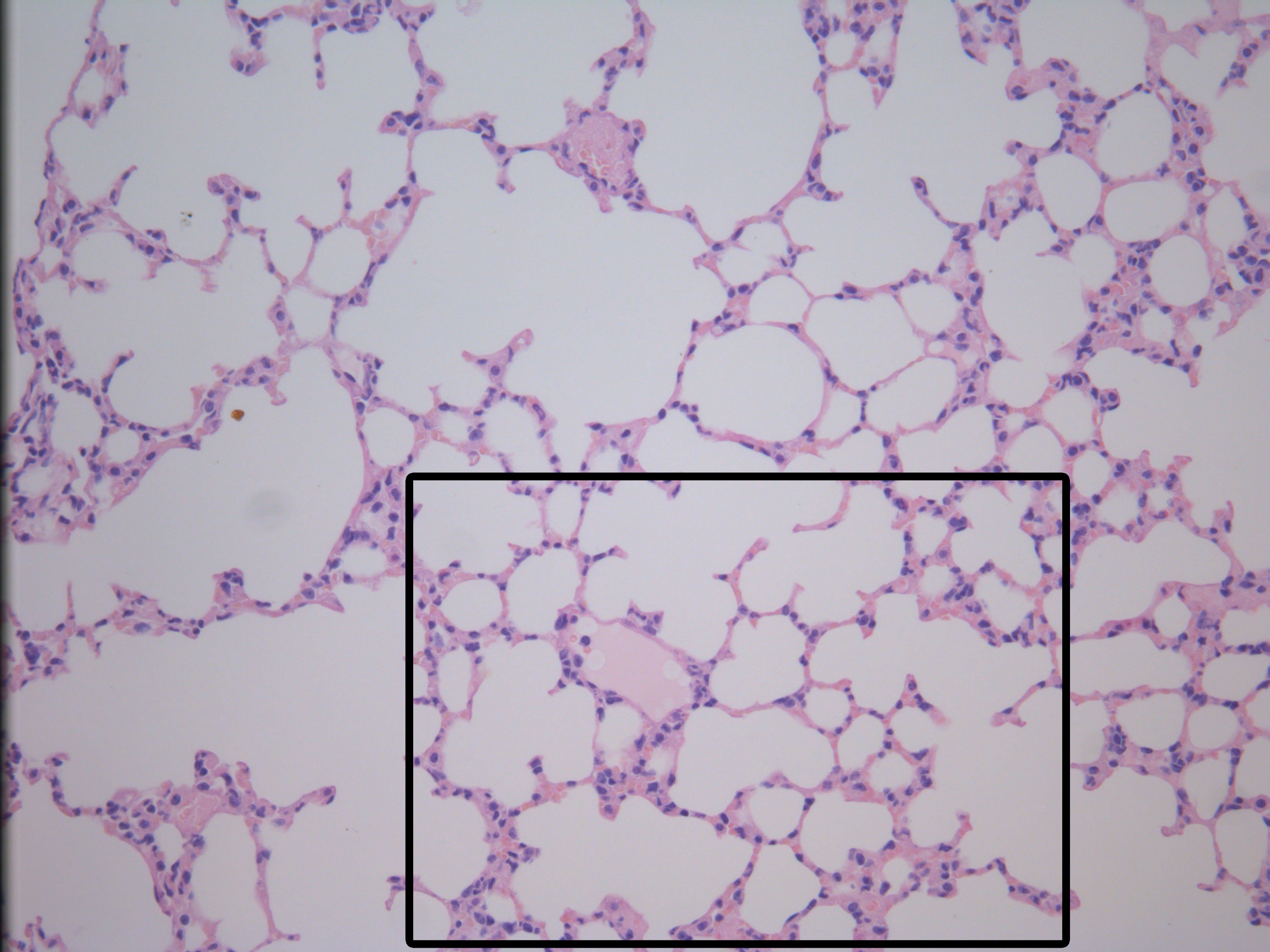

Supplement: S3 File — (ZIP) [file pone.0153540.s003.zip › S3 File/Fig.6E/LUNG/Adv-TK+GCV.jpg]

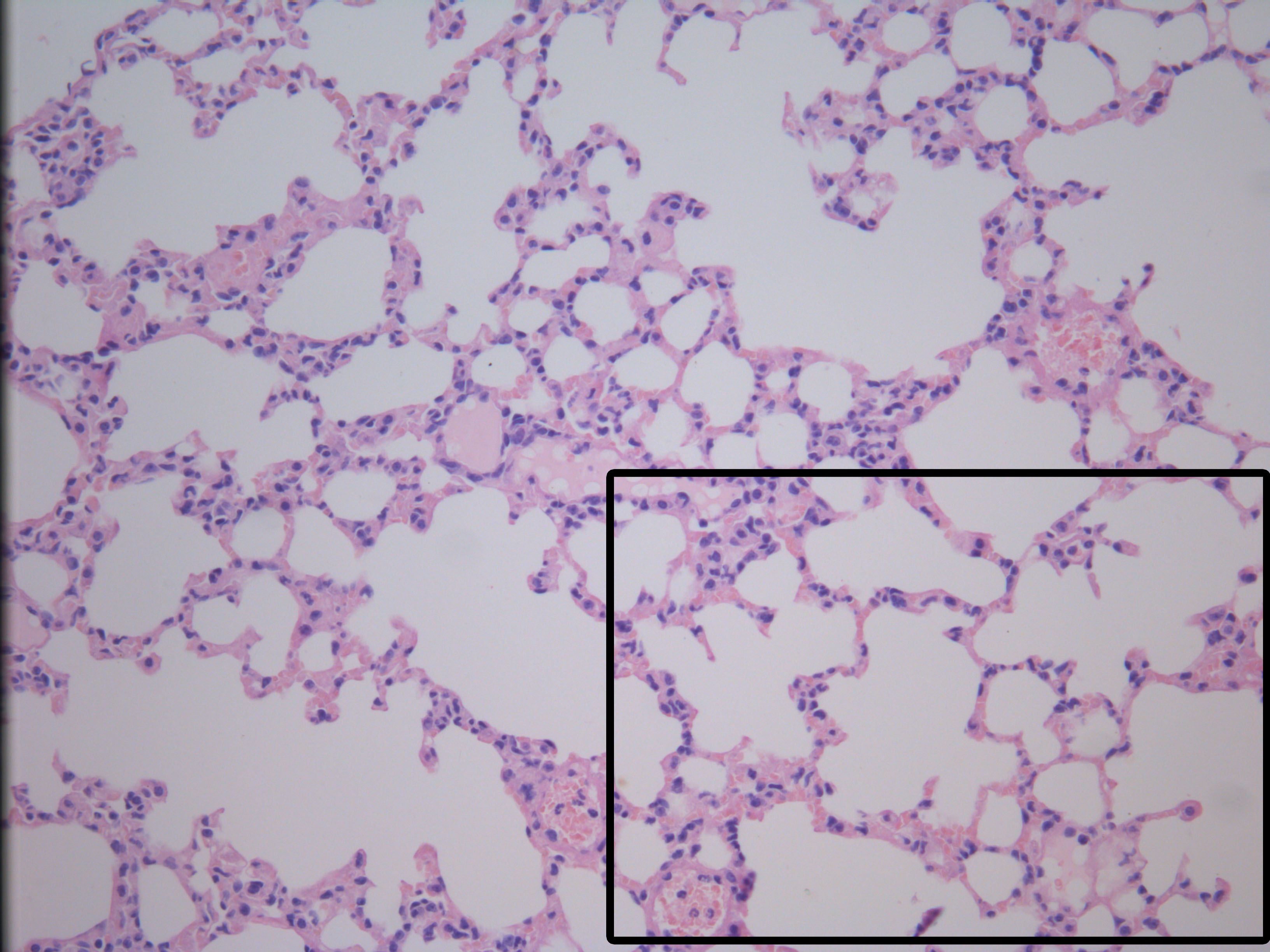

Supplement: S3 File — (ZIP) [file pone.0153540.s003.zip › S3 File/Fig.6E/LUNG/CONTROL.jpg]

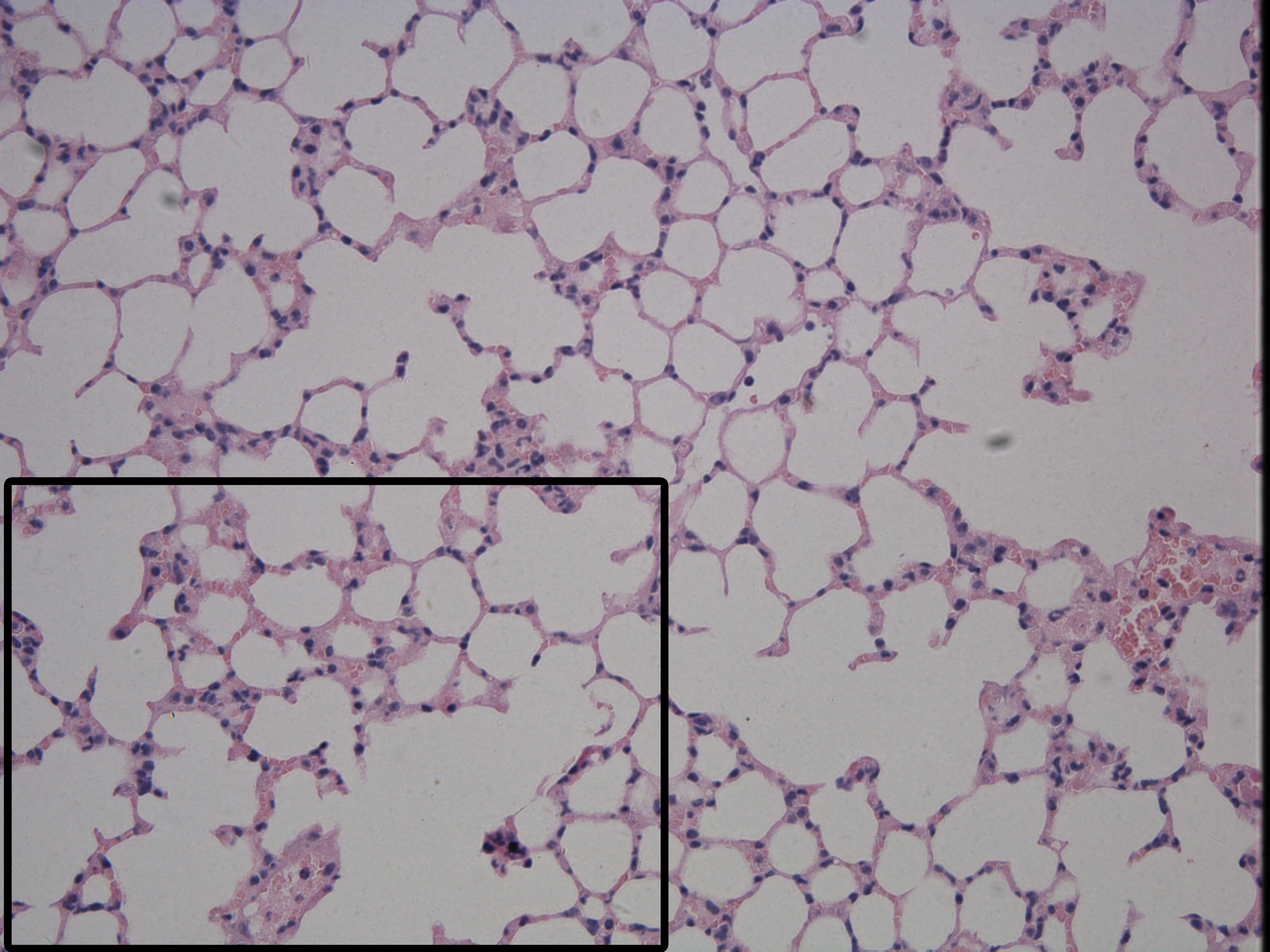

Supplement: S3 File — (ZIP) [file pone.0153540.s003.zip › S3 File/Fig.6E/LUNG/GCV.jpg]

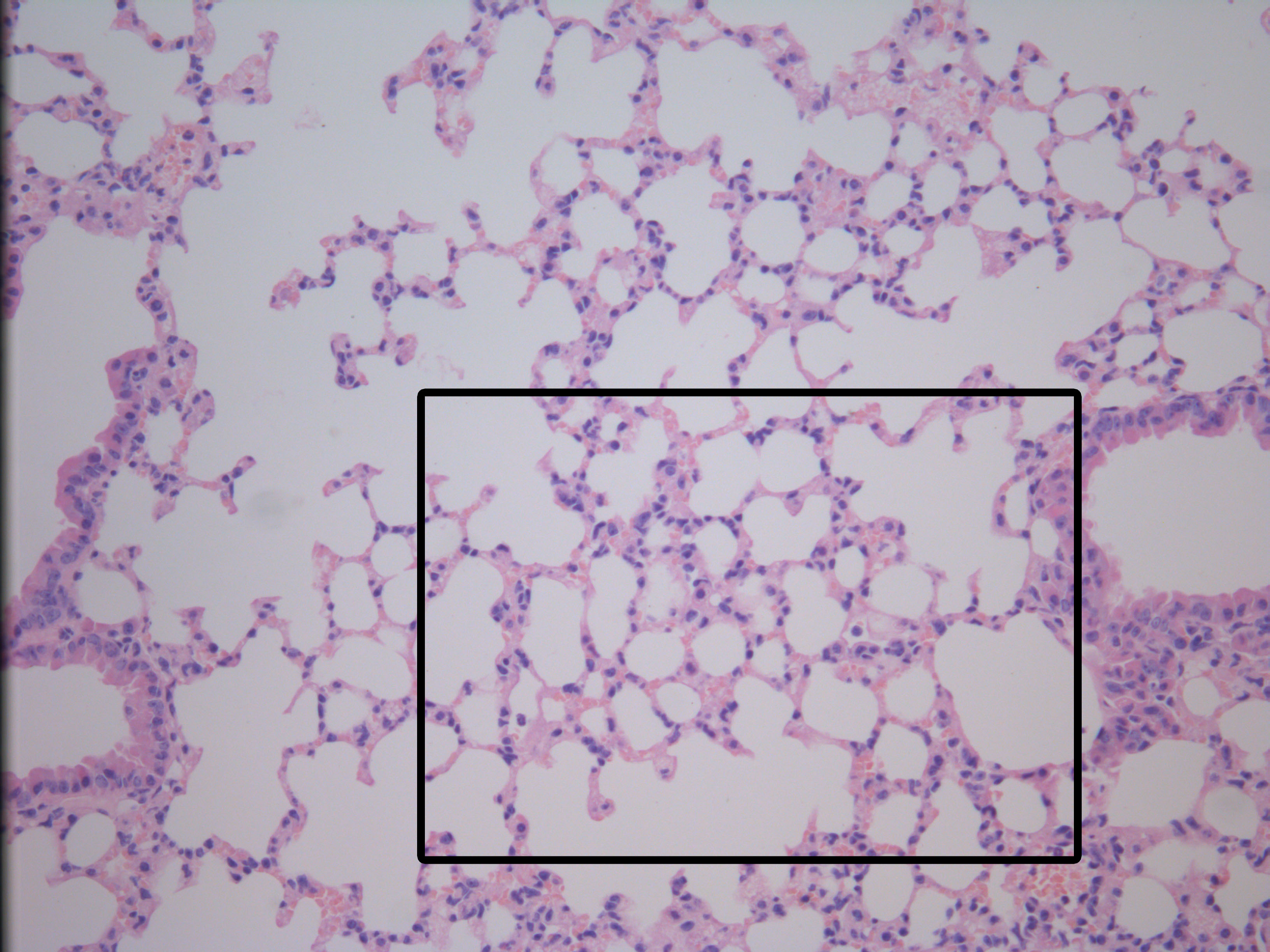

Supplement: S3 File — (ZIP) [file pone.0153540.s003.zip › S3 File/Fig.6E/LUNG/M7+GCV.jpg]

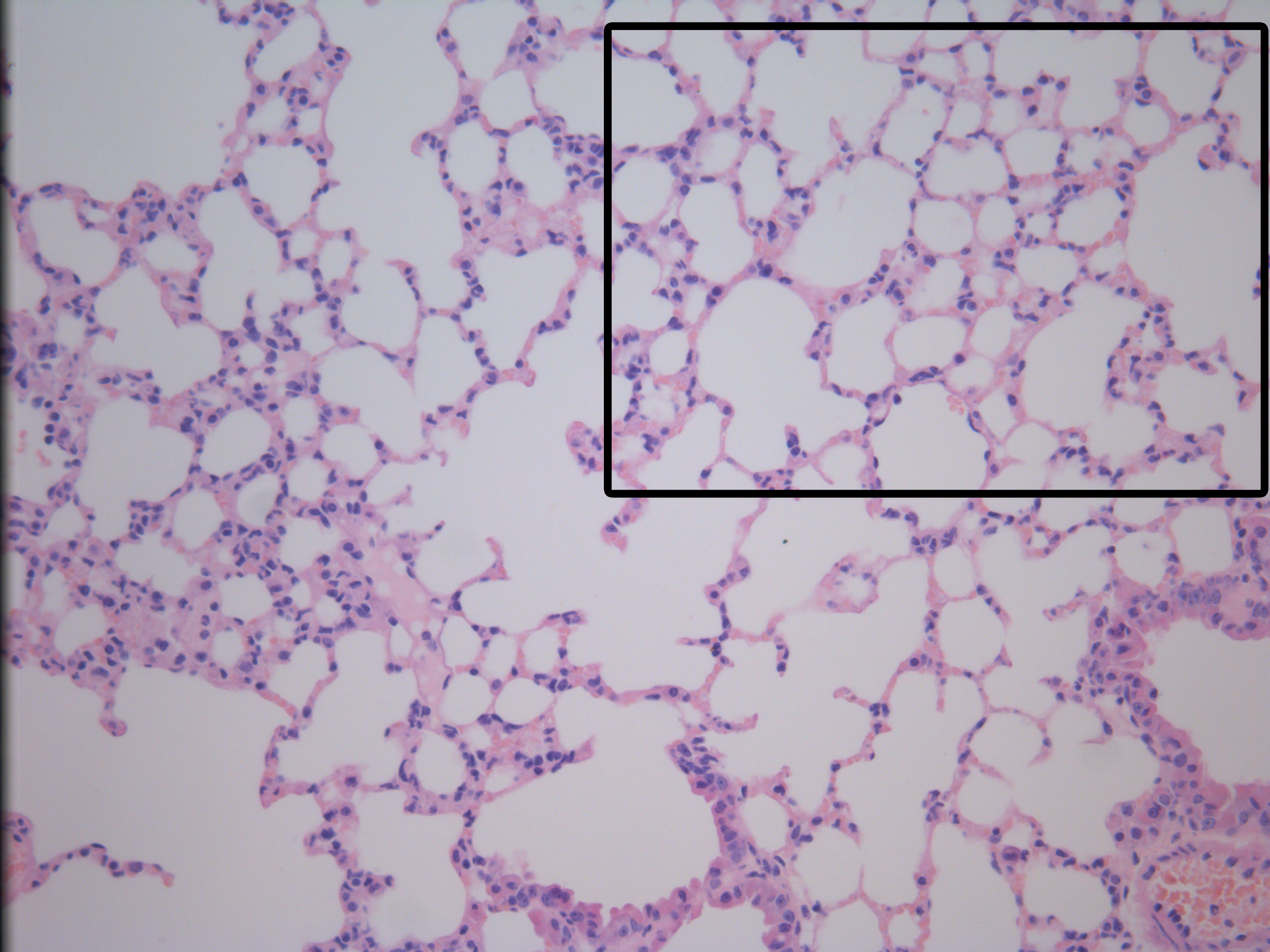

Supplement: S3 File — (ZIP) [file pone.0153540.s003.zip › S3 File/Fig.6E/LUNG/M8+GCV.jpg]

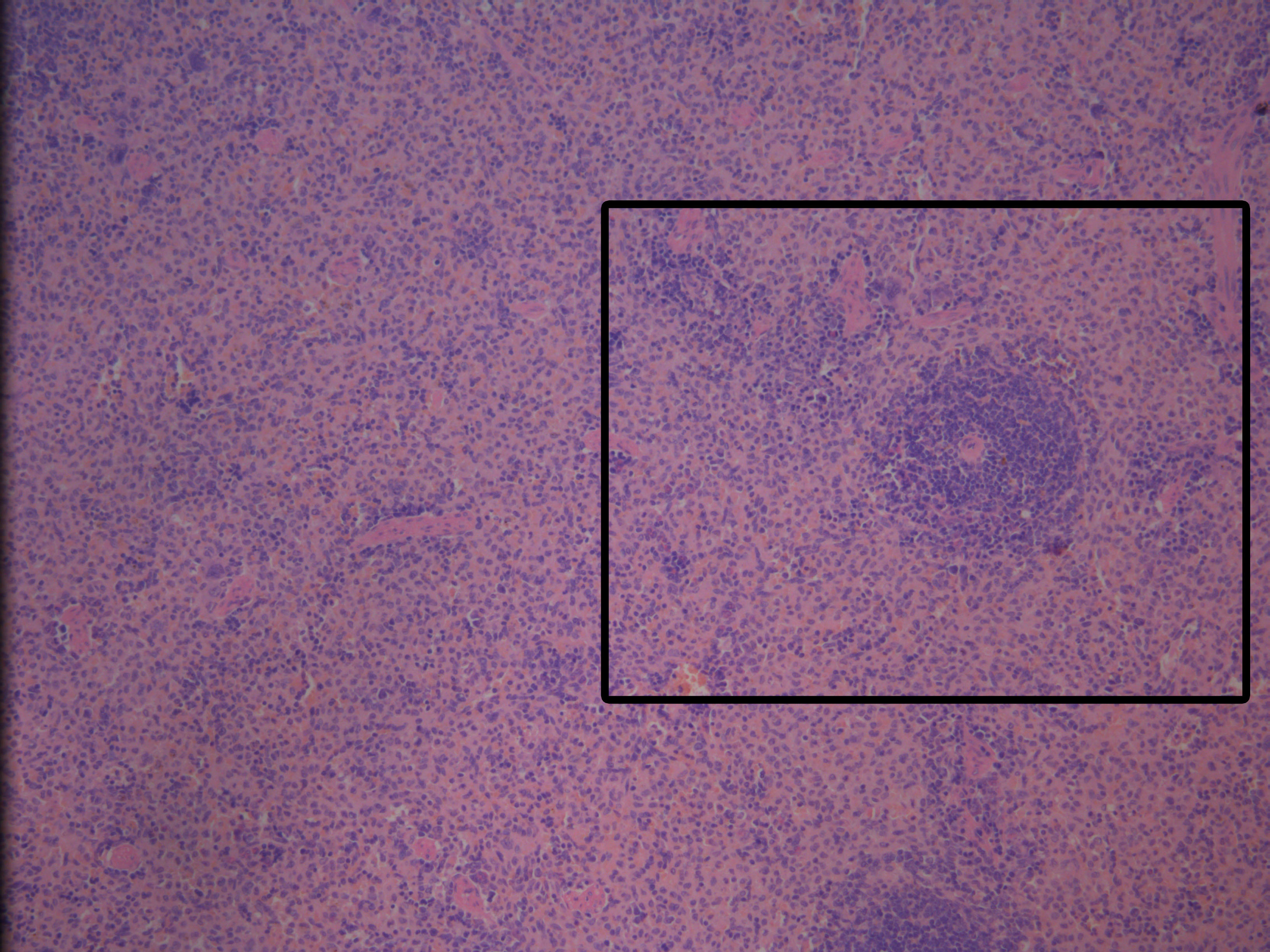

Supplement: S3 File — (ZIP) [file pone.0153540.s003.zip › S3 File/Fig.6E/SPLEEN/Adv-TK+GCV.jpg]

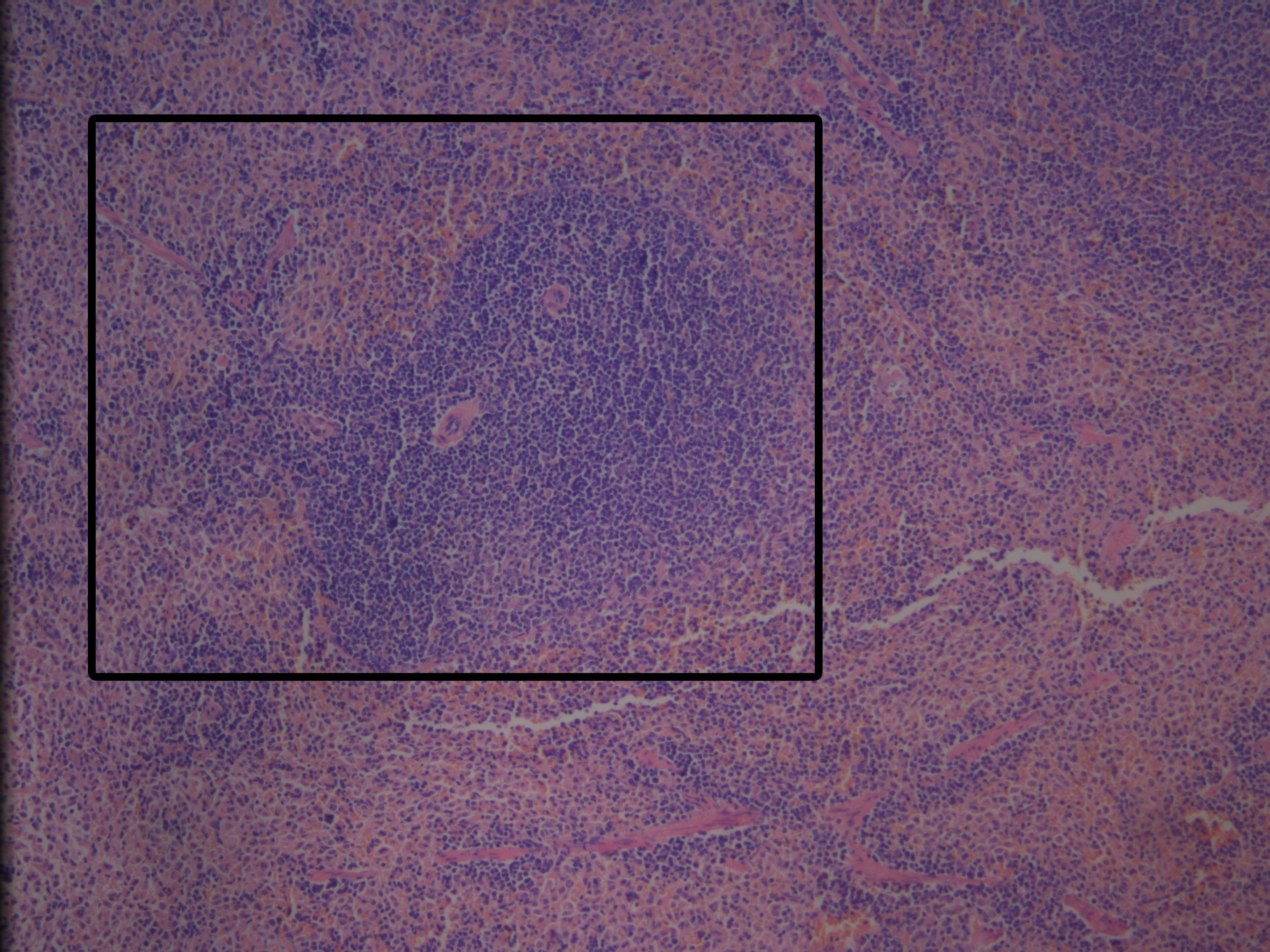

Supplement: S3 File — (ZIP) [file pone.0153540.s003.zip › S3 File/Fig.6E/SPLEEN/CONTROL.jpg]

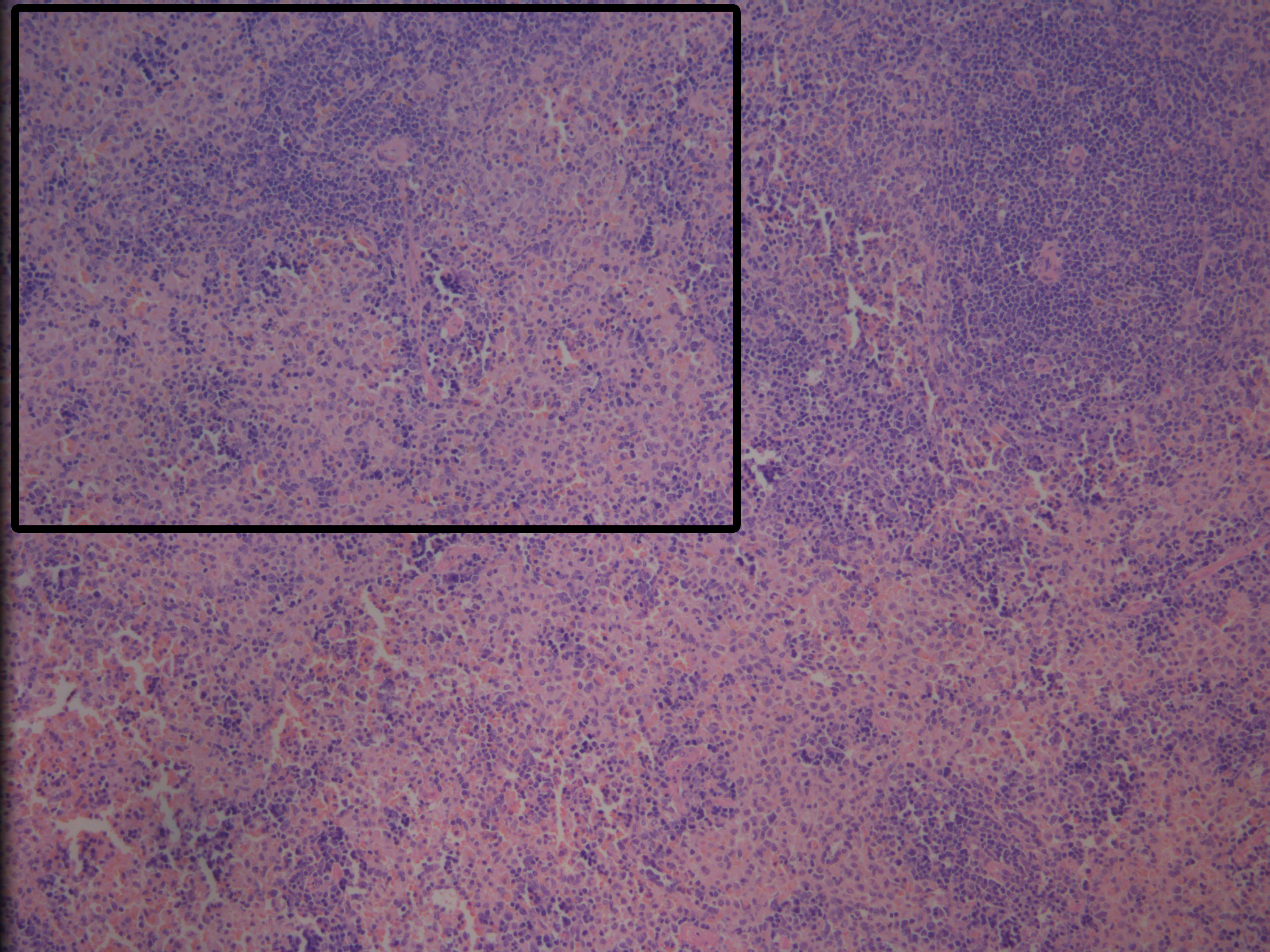

Supplement: S3 File — (ZIP) [file pone.0153540.s003.zip › S3 File/Fig.6E/SPLEEN/GCV.jpg]

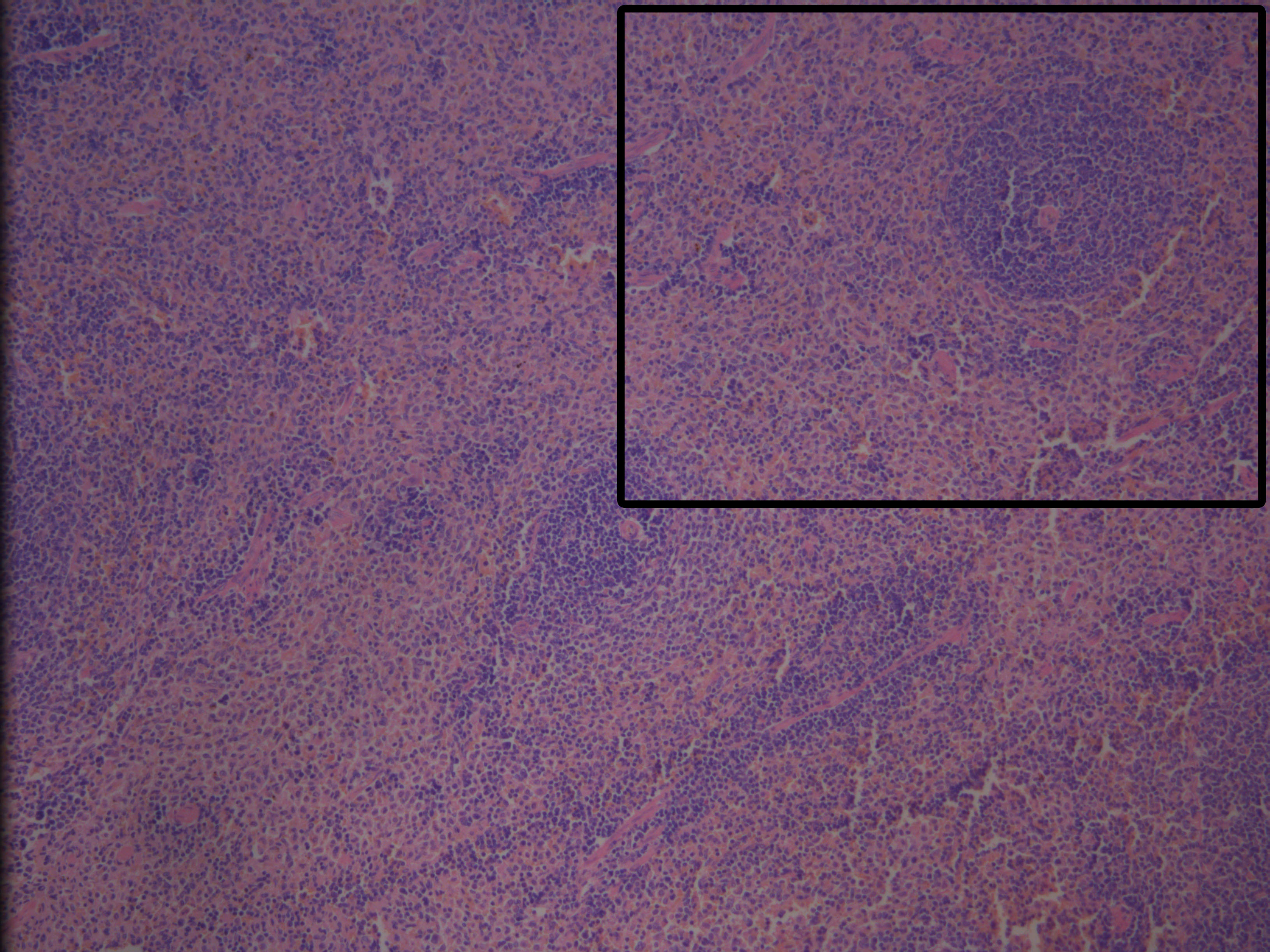

Supplement: S3 File — (ZIP) [file pone.0153540.s003.zip › S3 File/Fig.6E/SPLEEN/M7+GCV.jpg]

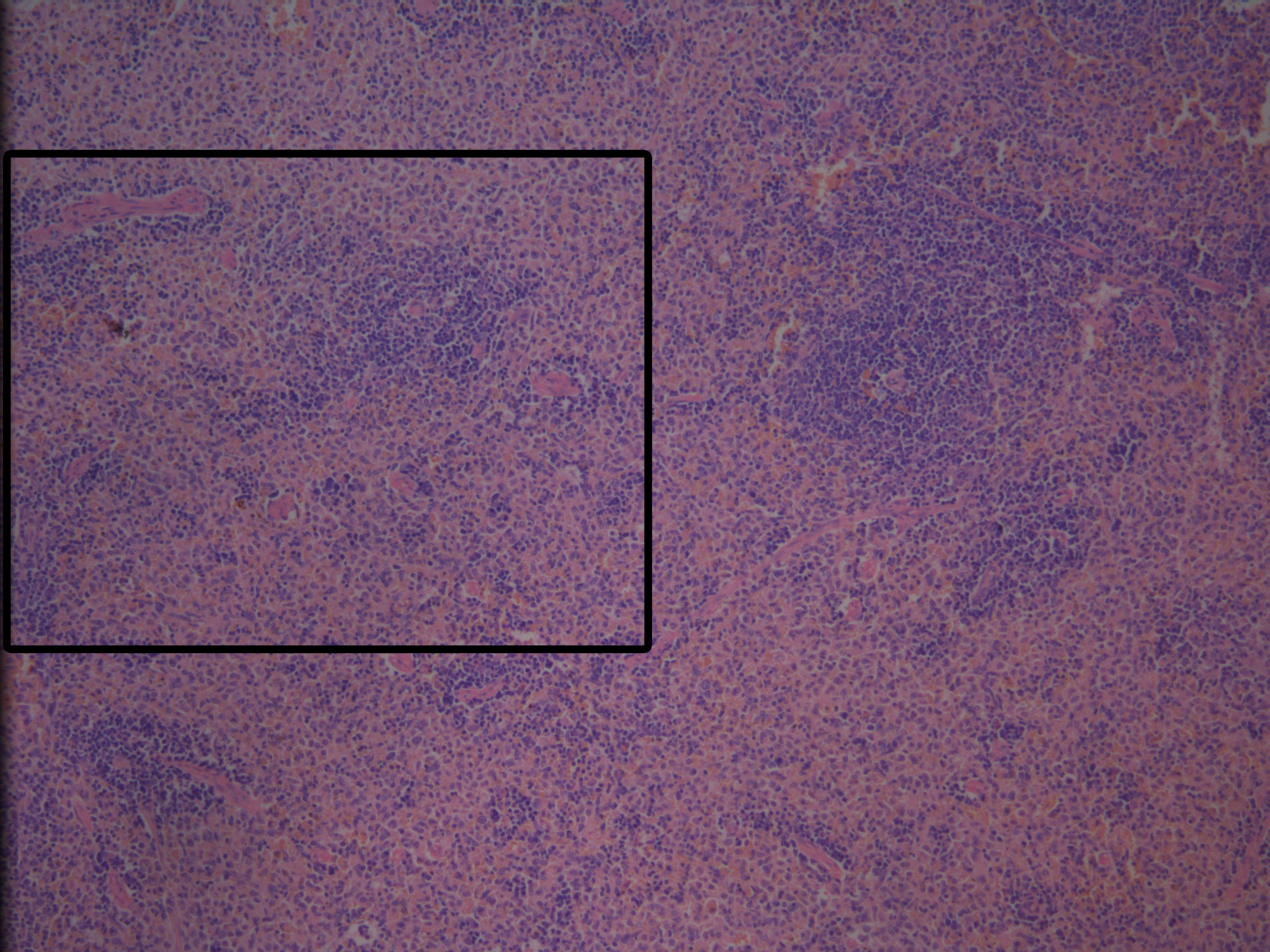

Supplement: S3 File — (ZIP) [file pone.0153540.s003.zip › S3 File/Fig.6E/SPLEEN/M8+GCV.jpg]

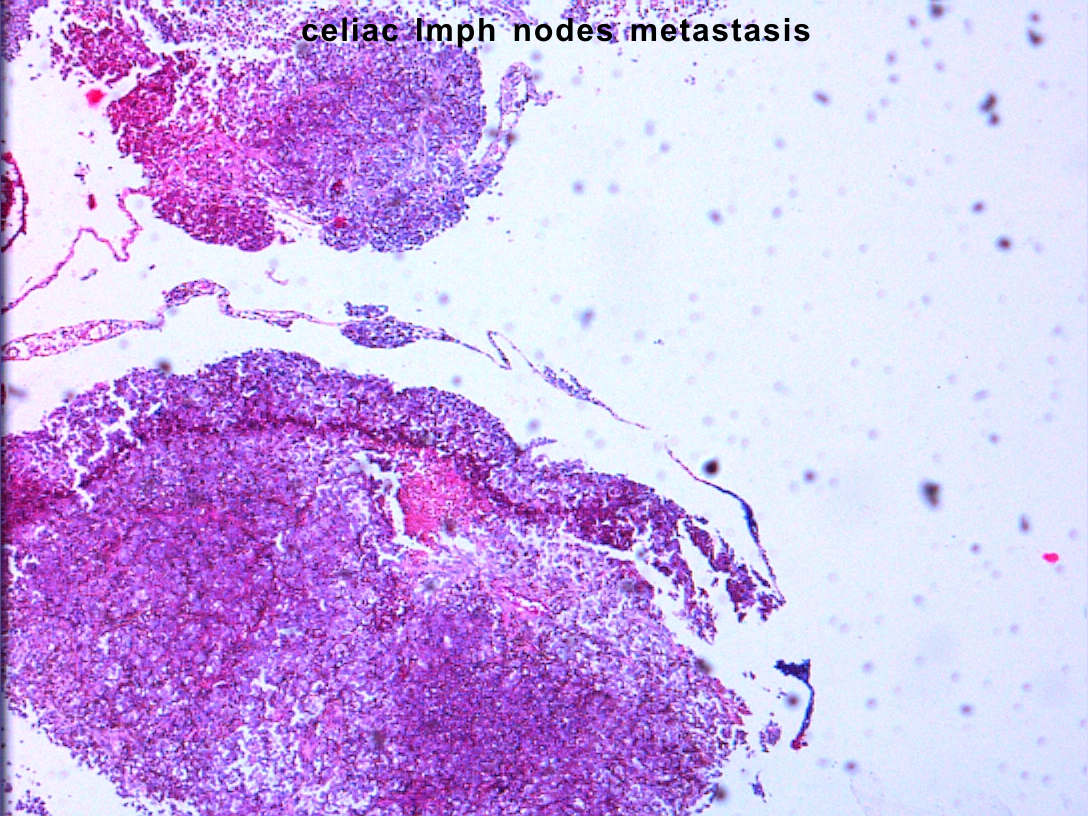

Supplement: S3 File — (ZIP) [file pone.0153540.s003.zip › S3 File/Supplementary Fig.8/S8A.tif]

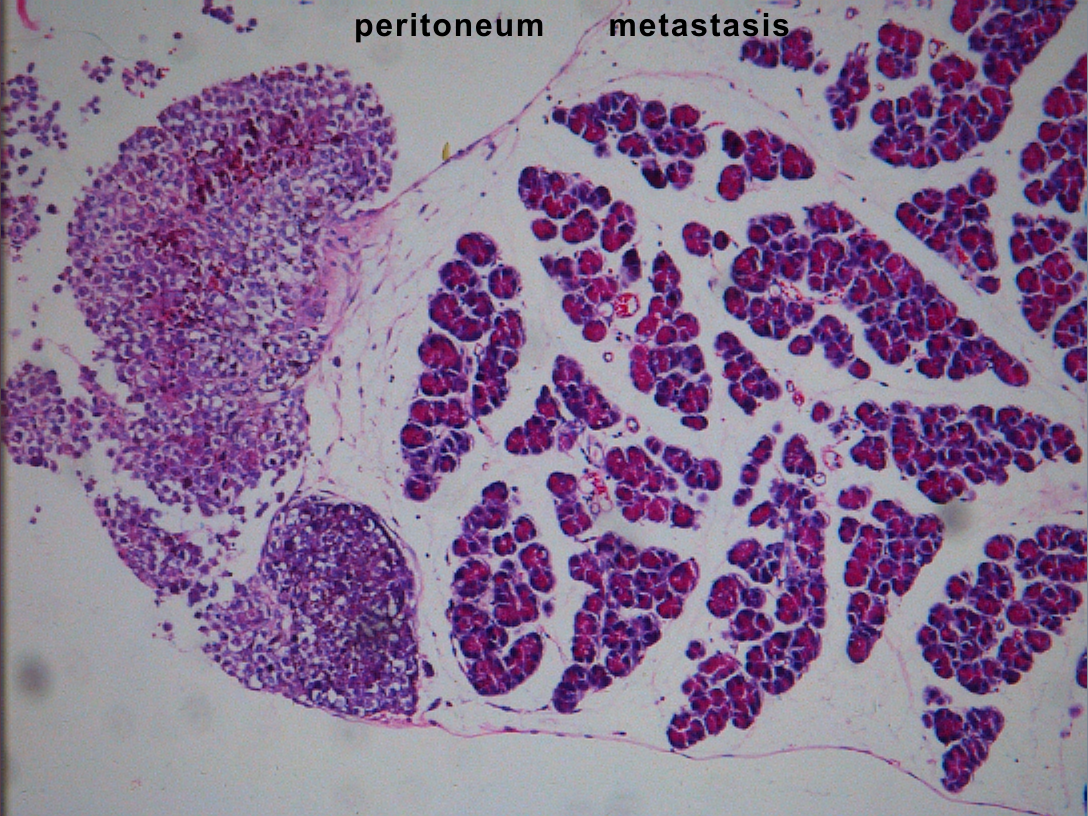

Supplement: S3 File — (ZIP) [file pone.0153540.s003.zip › S3 File/Supplementary Fig.8/S8B.tif]

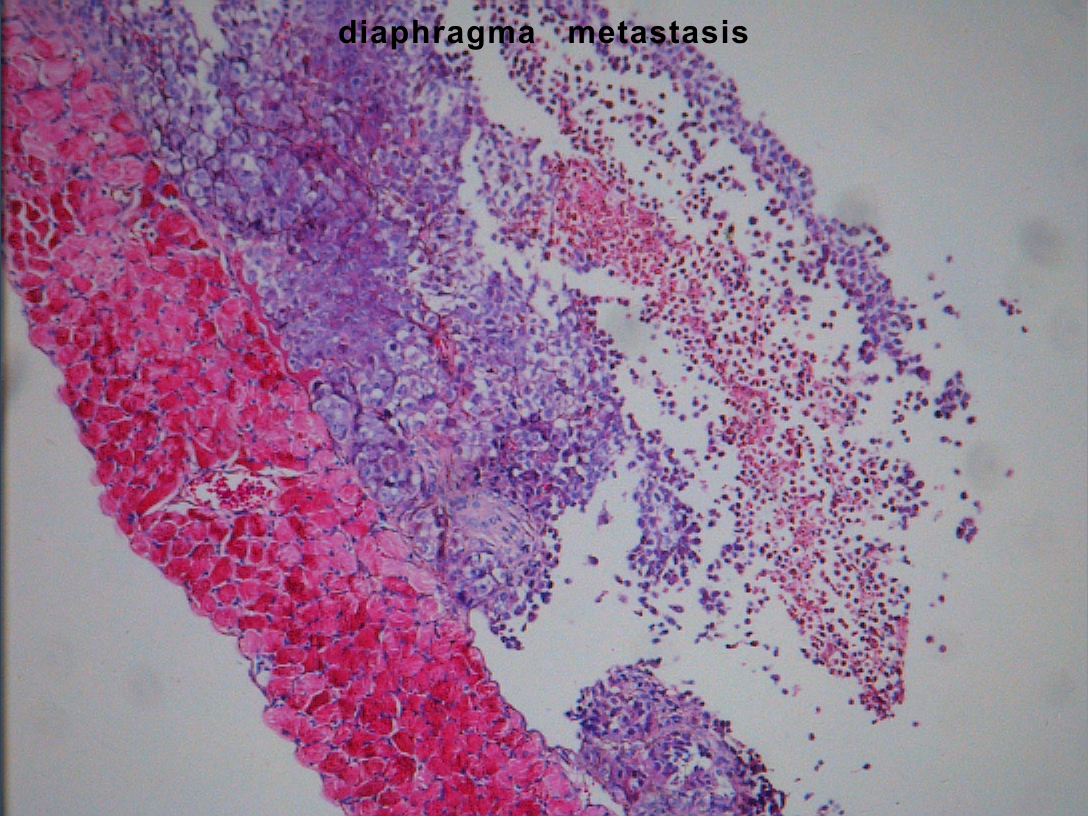

Supplement: S3 File — (ZIP) [file pone.0153540.s003.zip › S3 File/Supplementary Fig.8/S8C.tif]

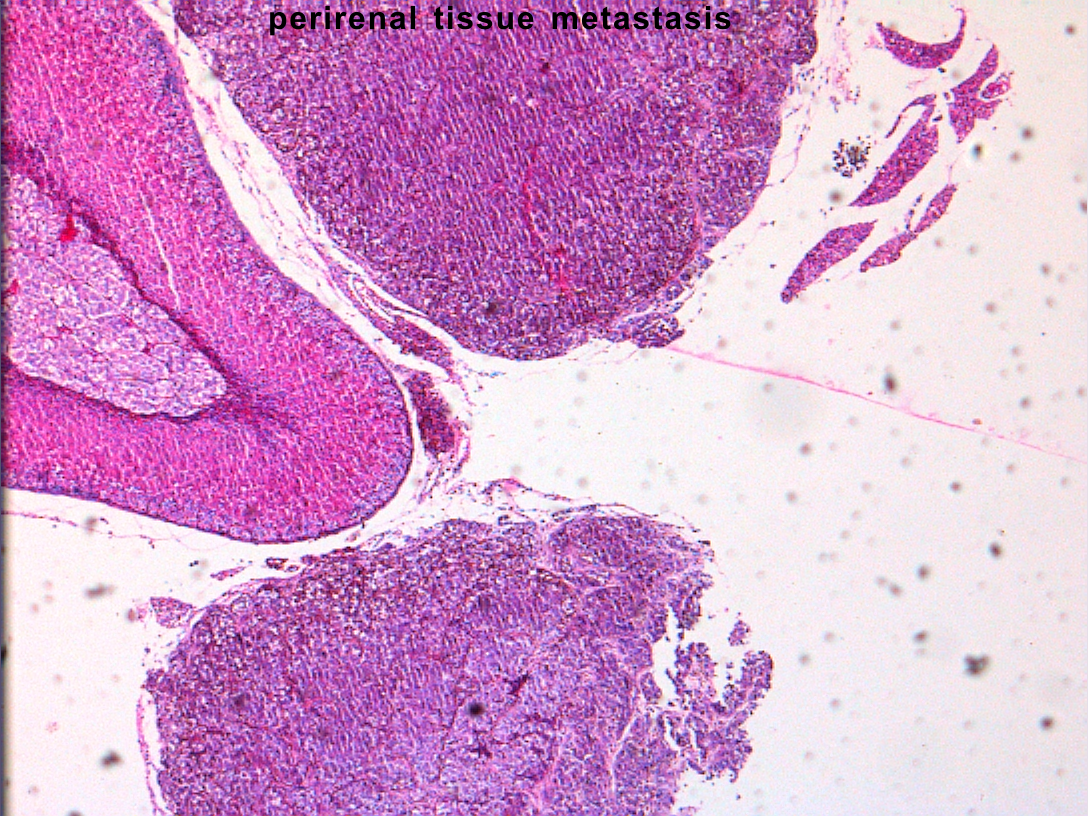

Supplement: S3 File — (ZIP) [file pone.0153540.s003.zip › S3 File/Supplementary Fig.8/S8D.tif]

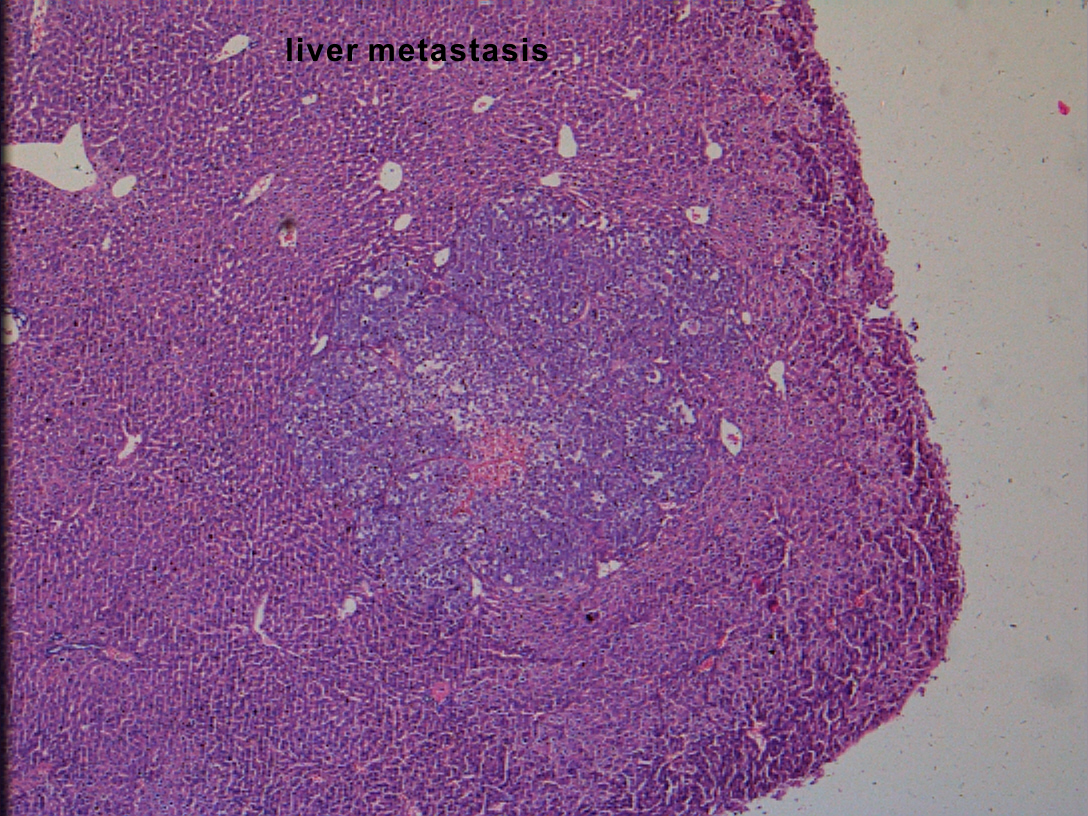

Supplement: S3 File — (ZIP) [file pone.0153540.s003.zip › S3 File/Supplementary Fig.8/S8E.tif]

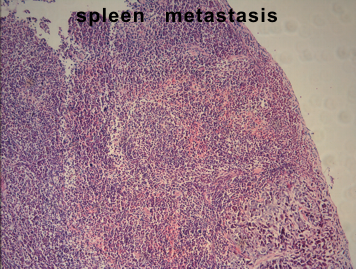

Supplement: S3 File — (ZIP) [file pone.0153540.s003.zip › S3 File/Supplementary Fig.8/S8F.tif]
