# Supplementary material for: Correction: A Systematic Comparison of the Anti-Tumoural Activity and Toxicity of the Three Adv-TKs
Source: PLoS One. 2016 Apr 7;11(4):e0153540. doi: 10.1371/journal.pone.0153540 (PMC4824467; doi:10.1371/journal.pone.0153540)
Supplement: S1 File — (ZIP) [file pone.0153540.s001.zip › S1 File/Fig.3/specimen 1.doc]

CONTROL+GCV

Ad5/dE1A/dgp19k+GCV

Ad5/dE1A/dADP+GCV

Adv-TK+GCV

M7+GCV

M8+GCV
